# From chromatogram to analyte to metabolite. How to pick horses for courses from the massive web resources for mass spectral plant metabolomics

**DOI:** 10.1093/gigascience/gix037

**Published:** 2017-05-17

**Authors:** Leonardo Perez de Souza, Thomas Naake, Takayuki Tohge, Alisdair R Fernie

**Affiliations:** Max-Planck-Institute of Molecular Plant Physiology, Am Mühlenberg 1, 14476 Potsdam-Golm, Germany

**Keywords:** Arabidopsis, bioinformatics, crop species, GC-MS, LC-MS, peak identification, peak annotation

## Abstract

The grand challenge currently facing metabolomics is the expansion of the coverage of the metabolome from a minor percentage of the metabolic complement of the cell toward the level of coverage afforded by other post-genomic technologies such as transcriptomics and proteomics. In plants, this problem is exacerbated by the sheer diversity of chemicals that constitute the metabolome, with the number of metabolites in the plant kingdom generally considered to be in excess of 200 000. In this review, we focus on web resources that can be exploited in order to improve analyte and ultimately metabolite identification and quantification. There is a wide range of available software that not only aids in this but also in the related area of peak alignment; however, for the uninitiated, choosing which program to use is a daunting task. For this reason, we provide an overview of the pros and cons of the software as well as comments regarding the level of programing skills required to effectively exploit their basic functions. In addition, the torrent of available genome and transcriptome sequences that followed the advent of next-generation sequencing has opened up further valuable resources for metabolite identification. All things considered, we posit that only via a continued communal sharing of information such as that deposited in the databases described within the article are we likely to be able to make significant headway toward improving our coverage of the plant metabolome.

## Background

Metabolomics emerged in the late 1990s, with the term coined in a review of Steven Oliver [1]. However, the 2000 paper by Fiehn and co-workers wherein gas chromatography (GC) coupled to mass spectrometry (MS) defined the chemical composition of a morphological and metabolic mutant of the model plant *Arabidopsis thaliana* [2]; in doing so, they were able to describe changes in the level of 326 analytes. This work thus greatly extended the early metabolite profiling study of Sauter et al. [3], which presented the technology as a means of putative classification of the mode-of-action of pesticides. Thus the advent of metabolomics in plants arguably preceded that in microbes and mammals although the approach was rapidly adopted in these communities also [2, 4–6]. During the next 2 decades, metabolomics had 1 considerable advantage over profiling technologies such as transcriptomics and proteomics in that it is not directly reliant on the genome sequence, and during this time the species scope of metabolomics rapidly expanded, such that it was no longer merely a tool for identifying biomarkers of cellular circumstance but additionally 1 of the cornerstones of systems biology and an approach that could provide mechanistic insight into metabolic regulation [7–11]. This advantage has subsequently disappeared following the widespread adoption of next-generation sequencing, and the lack of linear relationship between the genome and the metabolome now represents part of the problem in identification of unknown analytes [12]. This is nicely exemplified by the fact that computation of the size of the metabolome on genome information as attempted by Nobeli and co-workers in 2003 for the *Escherichia coli* metabolome [13] rendered values far smaller than the number of metabolites actually measured to date [14]. Whilst the size of the metabolome for prokaryotes has been estimated at a couple of thousand, that of the plant kingdom dwarves these numbers, with estimates ranging between 200 000 and 1 million metabolites [15]. Within the last 2 decades, metabolomics has been employed to address a wide range of important questions in plant biology, including pathway structure [15], the influence of metabolism on growth [8, 16], plant ecology [17], various aspects of plant genetics including evolution and the domestication syndrome [18–20], and detailed characterizations of the metabolic response to biotic and abiotic stressors [21, 22].

In this review, we discuss 2 topics. The first is the availability of tools to aid in chromatogram evaluation. Since we last reviewed this in 2009 [23], the number of resources has exploded, as has their diversity in type. In 2009, a number of pathway analytical standards, analytical samples, and literature databases were available. In the intervening period, additional sites providing information on experimental and *in silico* mass fragmentation, isotopic labeling, pathway predicted metabolites, integration of metabolomics with other platforms, and mass spectrometry imaging have become available. For each resource, we will briefly outline functionality and provide illustrative examples of their utility. The second is a review of the current status of the broad variety of plant metabolomics databases. In this respect, we list sources of archived data and their respective volumes of data. We also briefly discuss recent meta-analyses, which illustrate that despite current hurdles regarding comparability of data, there is great potential for cross-study comparisons on metabolite responses in determining common responses between either genetic or environmental perturbations of metabolism. Finally, we will provide an outlook as to how the grand challenge of comprehensitivity will best be met and how the power of archived plant metabolic responses will be best exploited in the future.

It is not the scope of this review to discuss the theoretical details of every procedure or to document the subtle differences between the many similar tools referred to here. We rather aim to provide a general idea of the importance and challenges of each step in the metabolomics workflow and to summarize the major functions of each tool while referring to the more comprehensive literature supporting them. We attempt to classify all the resources in a simple and logical manner in order to facilitate understanding of the main functionalities of each one. It is, however, important to mention that while few of the tools presented here provide a complete workflow, most of them are able to perform multiple complementary functions, somewhat blurring any initiative to accord their functions specific classifications. Other important information that we include here is how these tools can be accessed. This is usually performed either via command line or graphical user interface (GUI); the former provides flexibility and facilitates integration, automation, and development, while the latter was developed to be intuitive and friendly for unexperienced users. Finally, it is important to highlight that the active developments in the field result in frequently outdated and discontinued resources. While many groups keep releasing new upgraded versions of their tools, it is often the case that the projects are just discontinued and the tools are kept available online. We tried to represent this by including the most recent references as well as the last update dates for each of the resources in Supplementary Table 1. All these features considered allow the researcher to access the information required to choose the “winning horse” under the conditions or “course” in which they are racing. Finally, it is also important to highlight that these tools are constantly being updated, integrated, and discontinued, and while we ensured that all the links provided here were functioning at the time of writing, it is impossible to ensure that to be the case in the future.

## Sample Preparation and Data Acquisition

**Figure 1:**
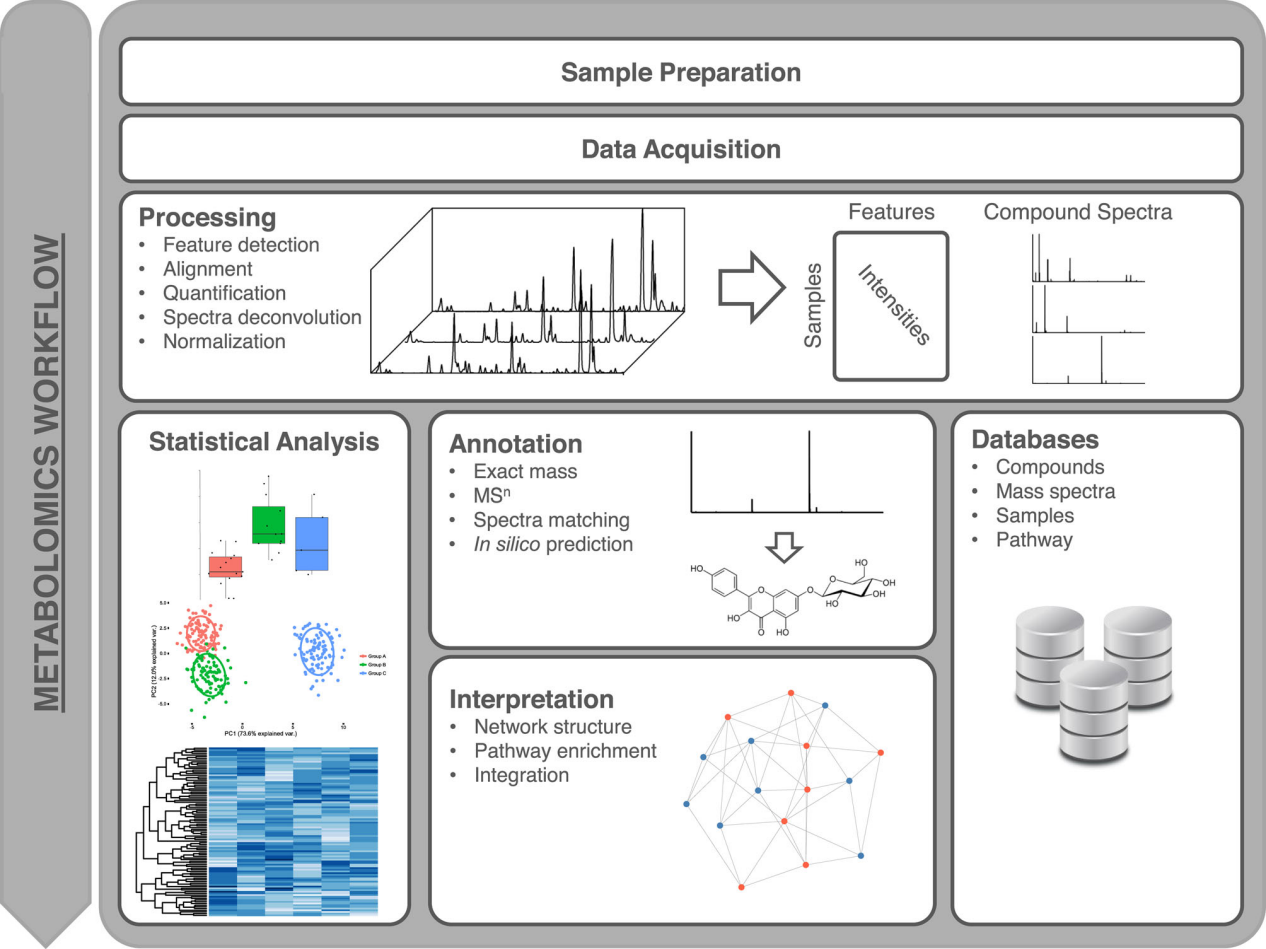
Typical mass spectrometry–based metabolomics workflow.

The metabolomics workflow (Fig. 1) starts with sample preparation including extraction and is often coupled to pre-treatment and chemical derivatization, followed by data acquisition, which will depend on the chromatographic system, ionization source, and analyzer. Optimization of sample preparation and data acquisition can considerably improve the analysis and is particularly interesting for plant metabolomics, where matrix complexity is very high; nevertheless, this step is often skipped over in favor of standardization and simplicity, which allow for greater sample throughput. Methods for chromatography mass spectrometry–based optimization are well developed and usually rely on statistical designs collectively known as design of experiments [24].

While some studies have detailed their application in plant metabolite extraction [25] and liquid chromatography (LC) analysis [26], very few software tools have been developed so far focusing on this kind of approach for metabolomics data. That said, a couple of interesting software packages have been published and appear to be highly promising: Multi-Platform Unbiased Optimization of Spectrometry via Closed-Loop Experimentation (MUSCLE) [27], a tool for the automated optimization of targeted LC-tandem mass spectrometry (MS/MS) analysis that was shown to significantly shorten analysis times and increase analytical sensitivities of targeted metabolite analysis, and FragPred [28], which uses experimental fragmentation from a database to select common fragmentation products that minimize uncertainty about metabolite identities in large-scale multiple reaction monitoring (MRM) experiments.

## Data Processing

Raw mass spectrometry chromatograms are 3-dimensional data consisting of a distribution of mass-to-charge ratio (m/z) intensities over the time. Processing this data requires filtering, detecting, and integrating relevant features, aligning signals across different samples, extracting compound mass spectra, and normalizing the data, all with the final goal of simplifying and hence facilitating data interpretation.

Feature detection and peak alignment are the initial steps for extracting information from raw data and correspond to the process in which relevant signals are identified and quantified across samples, having peak alignment as 1 of the big challenges to overcome, particularly for liquid chromatography mass spectrometry (LC-MS), where retention time is more prone to fluctuations in relation to gas chromatography mass spectrometry (GC-MS). The many different approaches available to perform these steps of data processing were recently reviewed by [29, 30], and some of the most popular algorithms for feature detection and peak alignment were compared in different works [31, 32]. Most software somehow integrates both steps in the same pipeline to generate a report of signal intensities over samples from raw data, and many of them also include some resources for data analysis and peak annotation, which will be discussed later in more detail. In the following section, we will detail the available tools for this step, adopting a similar approach in all subsequent sections also (the details of the programs are all given in Additional file 1). MetAlign [33] is a versatile tool that performs well with both LC-MS and GC-MS and allows direct conversion from and to vendor formats while most other tools need an extra software package for this step. It additionally provides a series of functionalities through other tools that are developed by the same group and integrates directly in the output of MetAlign. XCMS appears to be the most cited software for LC-MS data processing. It was developed for R and implements different algorithms for feature detection and alignment suitable for different kinds of data; while it can be argued that the software requires familiarity with programming and lacks resources for simple data inspection, its platform is, nevertheless, powerful and easily integrated with other tools, and its extensive community of users provides a great resource for troubleshooting. Moreover, a great number of other tools are built upon the functions of XCMS [34]. Amongst these, TracMass 2 [35], a MATLAB software that provides a GUI in a modular suite, was developed to provide immediate graphical feedback of every step of the processing pipeline. Its benchmark paper compared the complexity of different algorithms, highlighting the importance of low complexity when dealing with large data files and demonstrating it to be more efficient than MZmine 2 (see below for a discussion of this software) and comparable to XCMS, 2 of the most popular current data processing tools. The particularities of the TracMass algorithm make it more suitable for detecting mass traces in the low mass region that can be missed by other approaches. Intelligent Metabolomic Quantitation (iMet-Q) [36], a C# software with a GUI whose algorithm includes automatic detection of charge state and isotope ratio of detected peaks and that was developed to minimize the amount of necessary input parameters, significantly facilitates the pipeline for new users. GridMass [37] is a 2D feature detection algorithm implemented in MZmine 2 that is faster than other algorithms and potentially improves detection of low-intensity masses. Metabolomics Spectral Formatting, Alignment, and Conversion Tool (MSFACT) [38] was 1 of the first tools developed for peak alignment. It uses peak tables or raw data in the American Standard Code for Information Interchange (ASCII) format as input that is limited only to the chromatographic domain. This approach can, however, now be considered outdated when compared with many other resources currently available. Metabolomics Ion-Based Data Extraction Algorithm (MET-IDEA) [39] is a more recent and flexible tool, developed by the same group as MSFACT, for feature detection and alignment, with a friendly interface developed in the .NET platform. Its features include visualization of integrated peaks and manual integration and display of mass spectra, which can be very helpful for quick data inspection. EasyLCMS [40] is a web application tool with a focus on calibration and calculation of targeted metabolite concentration in terms of μmol using algorithms developed for MZmine 2. IDEOM [41] is a metabolomics pipeline using functions from XCMS and MZmatch from an Excel GUI. It also includes automated annotation based on an internal database of exact mass and retention time that can be updated by users according to the machine. Massifquant [42] is a feature detection algorithm integrated into XCMS based on a Kalman filter for the detection of isotope trace. This approach was shown to be particularly useful for low-intensity peaks. Metabolite Compound Feature Extraction and Annotatio (MET-COFEA) [43] is a C++ software accessed via a GUI that implements a novel mass trace-based extracted-ion chromatogram extraction that copes better with drifts in the mass trace. It additionally uses compound-associated peak clusters instead of individual features for alignment (this clustering process is an important step to extract metabolite information and simplify data, as will be discussed below). MET-Xalign [44] is an extension for MET-COFEA that can potentially align compounds of samples from different experiments, a hard task for metabolomics datasets that is not approached by most other tools. Adaptive Processing of High-Resolution LC-MS data (apLCMS) [45] is an R package for high mass accuracy LC-MS, which tries to be user friendly by providing a file-based operation and a wrapper function for a single command line batch process of LC-MS data, but still requires some computational knowledge to operate. xMSanalyzer [46] is an R package for improving feature detection that integrates with existing packages such as apLCMS and XCMS. It systematically re-extracts features with multiple parameter settings and merges data to optimize sensitivity and reliability. Yet Another Mass Spectrometry Software (yamss) [47] is a recently developed R package focused on providing high-quality differential analysis implementing a method based on bivariate approximate kernel density estimation for peak identification. In addition to the tools mentioned above, there are a few tools for data processing that exclusively perform peak detection or alignment, such as peak-grouping alignment [48], an approach where information from grouping peaks within samples improves alignment across samples, and parametric time warping [49], a fast alignment algorithm based on a variation of parametric time warping working on detected features rather than on complete profile data. In addition, combining single masses into quantities (cosmiq) [50] is a peak detection algorithm to improve detection of low abundant signals that can be easily integrated with XCMS. These algorithms represent an important effort in improving the existing approaches, but they are much less accessible since they need to be integrated with other tools that usually perform similar functions, and in some instances this requires quite advanced computational skill.

It is important to note the significant differences between GC-MS and LC-MS, which are intrinsic to the features of each system and can be summarized as a much higher efficiency and stability in GC over LC separation followed by a very stable fragmentation in traditional GC ion sources, in contrast with the typical atmospheric pressure ionization employed with LC. This significantly influences the processes of peak alignment and spectra annotation, and while most of the tools developed with a focus toward LC-MS can also be used for processing GC-MS data, there are many developed with a particular focus on processing GC-MS data, making use of different strategies for peak alignment and integrating metabolite annotation by matching spectra to libraries. Automated Mass Spectral Deconvolution and Identification System (AMDIS) [51], developed with the support of US Department of Defense, is 1 of the most popular GC-MS processing tools. It automatically extracts component mass spectra from GC-MS data and uses it to search mass spectral libraries. A disadvantage of this software is that the output requires extensive treatment to be used for further analysis. However, Metab [52], an R package based on functions of XCMS, was developed to automate the pipeline for analysis of GC-MS data processed by AMDIS facilitating the use of its results for further data analysis. MetaQuant [53] is a tool that uses a retention index to define metabolites, but it depends on other deconvolution software like AMDIS to extract mass spectra. Both MetaboliteDetector [54] and TagFinder [55] provide an efficient pipeline to perform deconvolution, peak detection, compound identification, and alignment based on Kovats retention index using alkane mix and quantification and provide an interactive user interface facilitating use by unexperienced users. They do, however, require several manual input and data check steps that are time consuming and negate truly high throughput. TargetSearch [56] uses similar approaches to process data and identify and quantify targeted metabolites based on retention time index and spectra-matching of multiple correlated masses, but they are highly automated and efficient, thus allowing the processing of large sample sets. PyMS [57] is an alternative to the previously mentioned interactive software, providing similar functions but being particularly suitable for scripting of customized processing pipelines and for data processing in batch mode working in Python. Metabolite Compound Feature Extraction and Identification (MET-COFEI) [58] uses reconstructed compound spectra instead of individual peaks to align signals across samples, which is expected to improve peak information for downstream analyses. It also matches the spectrum against a user-specific library. TNO-DECO [59] uses a segmentation approach to allow the performance of simultaneous deconvolution of multiple chromatographic MS files in a semi-automated fashion in MATLAB, thereby eliminating peak alignment. By contrast, MetaMS [60] is a pipeline for high-throughput GC-MS processing based on XCMS for peak detection and alignment and Collection of Algorithms for Metabolite Profile Annotation (CAMERA) for compound spectra extraction. Compound spectra is further annotated based on matching with a database. This tool may be convenient for users that already implement XCMS analysis of other data, but this kind of processing is not optimal for GC-MS when compared with other processing types. Maui-VIA [61] implements a graphical interface that facilitates visual inspection of identifications and alignments, providing faster interaction with the data. eRah [62] is an R tool that integrates a novel spectral deconvolution method using multivariate techniques based on blind source separation, alignment of spectra across samples without the need of internal standards for calculating retention indexes, quantification, and automated identification of metabolites by spectral library matching; in a fully automated pipeline, even though internal standards are not necessary, they are still recommended to increase reliability in metabolite identification. The software Automated Data Analysis Pipeline for Untargeted Metabolomics (ADAP-GC) 3.0 [63] uses a deconvolution algorithm based on hierarchical clustering of fragment ions. The updated version is incorporated into the MZmine 2 platform and addresses issues from the first version such as fragment ions that are produced by more than 1 co-eluting components, as well as improved sensitivity and robustness. Finally, MetPP [64] is a processing tool that includes normalization and statistical analysis but is directed toward data emanating from the GC × GC–time of flight MS system.

Extracting compound mass spectra is another important step of data processing that reduces data complexity by many orders of magnitude by identifying m/z signals that belong to the same compound and providing essential information for further metabolite annotation through the reconstructing of mass spectra. While this process is usually integrated in GC-MS tools for feature detection, alignment, and annotation, as mentioned above, there are many approaches to deal with LC-MS data, such as the ones employed by CAMERA [65], a package developed in R to extract compound spectra, annotate isotopes and adducts, and propose compound mass as an extension to XCMS. It is easy to use in combination with this software and provides a significant reduction on data complexity. AStream [66] is another R package very similar to CAMERA but using a simpler algorithm for grouping the peaks. ALLocator [67] is a web-based workflow that applies centwave from XCMS for feature detection, followed by spectra deconvolution either by CAMERA or by the ALLocatorSD algorithm, which is optimized for dealing with the particularities of ^13^C labeled data by grouping mirrored isotopes (lighter isotopologues from the feeding experiment). MSClust [68] has the same general features as the others, but it was developed in the C++ language and it is optimized to work with the output files of MetAlign. RAMClustR [69] was developed in MATLAB and implemented in R, accepting directly the output of XCMS. The authors suggest the use of a workflow consisting of data acquisition under both low and high collision energy as a way to improve the quality of the spectra generated by feature clustering and provide a data format that can be submitted directly to the MassBank Database and NIST MSSearch program. By contrast, Ratio Analysis of Mass Spectrometry (RAMSY) [70] uses average peak ratios and their standard deviations rather than correlation to allow the recovery of compound spectra. The performance of this approach is typically better than the results from correlation methods. Furthermore, the script for MATLAB is available, or it can be run from a web interface with a .csv table as input.

The last step of data processing, data normalization, is essential for further data analysis in order to remove bias introduced by sample preparation from meaningful biological variation. Most methodologies rely either on the use of internal standards or statistical means for normalization. Most data normalization procedures are usually integrated in data analysis tools, but there are few examples of more specialized tools such as MetTailor [71] that use a dynamic block summarization method for correcting misalignments, reducing missing data, and apply a retention time–based local normalization procedure, or Normalyzer [72], that uses 12 different well-known normalization methods and compares the results based on different parameters. IntCor [73] corrects for peak intensity drift effects based on variance analysis, MetNormalizer [74] allows for normalization and integration of multiple batches in large-scale experiments using support vector regression, and EigenMS [75] detects biased trends in the data and eliminates them using single-value decomposition. All of these software packages are highly useful and are implemented in R; however, with the exception of Normalyzer, which can be also used in a web interface, they all require considerable familiarity with this programing language. A couple of other tools that help to extract specific information previous to data analysis include the program SpectConnect [76], which identifies conserved metabolites in GC-MS datasets, and the Mathematica package for Differential Analysis of Metabolite Profiles (MathDAMP) [77], which highlights differences within raw LC-MS and GC-MS datasets.

A common feature of mass spectrometry data is the presence of multiple peaks for individual fragments resulting from the distribution of natural isotopes, which are particularly interesting and explored in stable isotope labeling experiments. There are a few tools for correcting and extracting label enrichment from processed data, such as Corrector [78], IsoCor [79], and Isotope Correction Toolbox (ICT) [80]. These tools are very similar, all being based on the same matrix calculation. Corrector was developed to work on the output of TagFinder, but data processed with most other tools can be easily arranged in a similar table format. IsoCor provides a GUI with a few different options, including corrections for the label input, whereas ICT includes features to process data from tandem MS. Nevertheless, most data processing pipelines available are not particularly efficient for dealing with this kind of experiment. To fill this gap, there are some specialized tools like mzMatch–ISO [81], integrated in the mzMatch pipeline. This software is capable of targeted and untargeted processing of labeled datasets, and the output includes a set of plots summarizing the pattern of labeling observed per peak, allowing users to quickly explore data. MetExtract [82] relies on a mixture of cultures from the same organism under natural and labeled media to select signals that show a clear pattern of isotopic enrichment. However, the approach requires the labeled fraction to be fully labeled and the tracer to be highly pure to get the proper isotopic distributions. X13CMS [83] and geoRge [84], both run on the R platform using GC-MS output. The former algorithm iterates over MS signals in each mass spectra using the mass difference due to the label, while the latter uses statistical testing to distinguish spectral peaks originating from labeled metabolites, resulting in significantly less false positives. The Mass Isotopolome Analyzer (MIA) program [85] detects isotopic enrichment in GC-MS datasets in a non-targeted manner, providing an easy GUI to visualize mass isotopomer distributions (MID) of the detected fragments as barplots, including confidence intervals and quality measures, tools for differential analysis of relative mass isotopomer abundance across samples and network assembly based on pairwise similarity of MID that can reveal related metabolites.

Another important feature of many mass spectrometry systems is their capability of performing tandem mass spectrometry. While this can significantly improve data in many ways, it adds another level of complexity for data processing. A very common use of tandem MS is to increase selectivity and accuracy in targeted analysis, and MRMAnalyzer [86], Metabolite Mass Spectrometry Analysis Tool (MMSAT) [87], and Multiple Reaction Monitoring–Based Probabilistic System (MRMPROBS) [88] are useful tools developed for processing data from multiple reaction monitoring experiments. MMSAT [87] is a web tool that takes mzXML files as the input. It is able to automatically quantify MRM peaks but lacks metabolite identification capability. By contrast, MRMPROBS [88] detects and identifies metabolites automatically, providing a user-friendly GUI for data analysis. The algorithm has 1 limitation, that it needs at least 2 transitions per metabolite in order to discriminate the target metabolite form isomeric metabolites and background noise. Similarly, MRMAnalyzer [86] is an R tool that allows for processing, alignment, metabolite identification, quality control check, and statistical analysis of large datasets and transforms data in “pseudo” accurate m/z in order to use the centwave algorithm from XCMS for peak detection. Untargeted metabolomics analysis can also take advantage of tandem MS, particularly for compound annotation, and there are a few resources for dealing with the complexity of such experiments, such as decoMS2 [89], an R package for deconvoluting MS2 spectra, eliminating contaminating fragments without the need of sacrificing sensitivity in favor of sensibility by narrowing the window of isolation for collision-induced dissociation (CID) during data acquisition. This approach requires MS2 data to be acquired under low and high collision energies to solve the mathematical equations, potentially reducing the sensitivity of the method. Mass Spectrometry–Data Independent Analysis (MS-DIAL) [90] and MetDIA [91] both deal with data-independent acquisition (DIA) data, an interesting approach for untargeted metabolomics that acquire MS2 spectra for all precursor ions simultaneously, with the complication that it uses larger isolation windows, hence increasing the probability of contamination in the MS2, and it loses the relation between precursor and fragment ions. MS-DIAL addresses these problems by a mathematical deconvolution based on GC-MS processing tools in a fully untargeted manner, whilst achieving the metabolite identification through a spectrum-centric library matching. MS-DIAL is applicable to both data-independent and data-dependent MS/MS fragmentation methods in LC-MS and GC-MS. By contrast, MetDIA [91] uses algorithms from XCMS for peak detection and alignment, combined with a targeted approach based on matching metabolites in a library to the detected peaks, thus achieving higher sensitivity and specificity on metabolite identification and wider metabolite coverage.

A trade-off for most of the more flexible and powerful resources presented here is that they have multiple parameters that need to be optimized, and recently a number of tools have tried to assist in evaluating and automatizing this process. In this context, Isotopologue Parameter Optimization (IPO) [92] was developed to perform automatic optimization of XCMS parameters based on design of experiment. Credentialing features [93] optimize detection based on regular and 13C-enriched. MetaboQC [94] is a quality control approach that evaluates alignment and suggests optimal parameters for feature detection based on discrepancies between replicate samples, and SIMAT [95] allows the selection of the optimal set of fragments and retention time windows for target analytes in GC–single ion monitoring–MS-based analysis.

## Data Analysis

Metabolomics datasets are usually characterized by high dimensionality, heteroscedasticity (i.e., the variance in errors is not constant across the dataset), and differences of orders of magnitude across metabolite concentrations and fold changes, making it challenging to extract and visualize useful information from processed data. There are numerous approaches for data scaling, reduction, visualization, and statistical analysis that are particularly useful for analyzing metabolomics data, many of them very well established, such as analysis of variance (ANOVA), hierarchical cluster analysis (HCS), principal component analysis (PCA), and partial least squares discriminant analysis (PLS-DA) to mention just a few. There are many general statistical software packages capable of performing most of these functions, but also a variety of software tools exist that combine procedures relevant to metabolomics in a single pipeline, thus facilitating the workflow, such as DeviumWeb [96], BioStatFlow [97], MetaboLyzer [98], metaP-Server [99], Fusion [100], Pathomx [101], MSPrep [102], MixOmics [103], and Covariance Inverse (COVAIN) [104].

Other interesting and somehow more specialized tools include RepExplore [105], which exploits information from technical replicate variance to improve statistics of differential expression and abundance of omics datasets, and Kernel Machine Approach for Differential Expression Analysis of Mass Spectrometry–Based Metabolomics Data (KMMDA) [106] and Metabomxtr [107], which deal with the troublesome issue of missing metabolite values, the former through a kernel-based score test and the latter through mixed-model analysis. Similarly, PeakANOVA [108] identifies peaks that are likely to be associated with 1 compound and uses them to improve accuracy of quantification, a particularly useful approach for experiments with limited sample size. Selective Paired Ion Contrast (SPICA) [109] is a tool that aims at extracting relevant information from noisy datasets by analyzing ion pairs instead of individual ions. MetabR [110] normalizes data using linear mixed models and tests for treatment effects with ANOVA. By contrast, Model Population Analysis–Random Forests (MPA-RF) [111] combines random forests with model population analysis for selecting informative metabolites. Qcscreen [112] helps to verify data consistency, measurement precision, and stability of large-scale biological experiments.

## Metabolite Annotation

Metabolite annotation is often considered the most challenging step and as such represents a major bottleneck for metabolomics studies. Even though the gold standard for structural characterization remains NMR characterization of the pure compound [113, 114], MS-based metabolomics offers many advantages, including lower cost, higher sensitivity and throughput, and it can be easily hyphenated with chromatography while still providing considerable structural information. As a consequence, great efforts have been made to improve mass spectrometry–based metabolite annotation, and a battery of interesting tools have been developed with this goal in mind. The great interest from metabolomics and mass spectrometry communities even culminated with the creation of the “Critical Assessment of Small Molecule Identification” (CASMI) contest. The idea of the contest is to challenge multiple approaches and rank their performance over a series of categories [115, 116]. Structural information is normally extracted from mass of molecular ion in high-resolution MS (HRMS), which can provide the molecular formula and fragmentation pattern. It is important to note that most strategies for metabolite annotation rely heavily on information retrieved from databases of molecular formulas, spectra, and pathways, which will be discussed in more detail below.

The most common tools are based on matching spectra or exact masses from unknown compounds against spectral data deposited in some database. One example using this approach is MetaboSearch [117], which accepts either a list of m/z or the output of CAMERA as input and searches against 4 major metabolite databases, the Human Metabolome DataBase (HMDB), Madison Metabolomics Consortium Database (MMCD), Metlin, and LipidMaps. Similarly, PUTMEDID-LCMS [118], developed in the Taverna Workflow Management System, also integrates a step of compound mass spectra extraction to define a molecular formula from high-resolution m/z that is then matched against a predefined list of molecular formulas to annotate compounds. MetAssign [119] is integrated in mzMatch, and it considers the uncertainty related with metabolite annotation using a Bayesian clustering approach to assign peak groups. This approach has the advantage of providing a quantitative value for uncertainty/confidence in the outputs that can be used in further analysis. The program Sum Formula Identification by Ranking Isotope Patterns Using Mass Spectrometry (SIRIUS) [120] is a Java-based software that combines high-accuracy mass with isotopic pattern analysis to distinguish even molecular formulas in higher-mass regions. Furthermore, it also analyses the fragmentation pattern of a compound using fragmentation trees that can be directly uploaded to compound structure identification: FingerID (CSI: FingerID; described below) via a web service. Molecular Formula Searcher (MFSearcher) [121] is a tool that efficiently searches high-accuracy masses against a database of pre-calculated molecular formulas with fixed kinds and numbers of atoms that are further queried against different databases. HR3 [122] is a similar tool for molecular formula calculation and query in external databases. It uses different sets of rules for heuristic filtering of candidate formulas instead of a pre-calculated database, which makes it slightly slower than MFSearcher, but HR3 includes compounds with atoms that are not present in MFSeacher's list, as well as considering matches to the isotopic pattern within its annotations. MS-FINDER [123] is a C# program with a GUI providing a constraint-based filtering method for selecting structure candidates. The workflow begins with molecular formulas from precursor ions being determined from accurate mass, isotope ratio, and product ion information. Next, structures of predicted formulas are retrieved from databases, MS/MS fragmentations are predicted, and the structures are ranked considering bond dissociation energies, mass accuracies, fragment linkages, and, most importantly, 9 hydrogen dissociation rules. MS-FINDER provides an interesting theoretical background from which to interpret MS/MS spectra and their comparison to database matches. Additionally, it was shown to be able to predict with 91.8% accuracy over 80% of the manually annotated metabolites in test samples [123]. MS2Analyzer [124] is a Java software for identifying neutral losses, precursor ions, product ions, and m/z differences from MS2 spectra based on a list of predefined transitions. These features are essential for structure elucidation using mass spectrometry, and the software provides a fast and high-throughput platform for extracting this data. MS2LDA [125] is based on latent Dirichlet allocation (LDA), an algorithm originally used for text mining that was adapted to generate a list with blocks of co-occurring fragments and losses, providing results similar to MS2Analyzer but without the need of user-specified precursor/product transitions.

Another level of biologically relevant information is added by many tools that incorporate pathway information to assist annotation and interpretation of results, such as Metabolome searcher [126], a web-based application to directly search genome-constructed metabolic databases, which includes MetaCyc with data on plant metabolism. MassTRIX [127] is a web interface that takes a mass peak list from HRMS as input and matches it against a Kyoto Encyclopedia of Genes and Genomes (KEGG) compounds database, returning a pathway map with the matches. Organisms can be selected, and the output represents organism-specific and extra-organism items, differentially colored to assist interpretation. MetabNet [128] is an R package to perform targeted metabolome-wide association study of specific metabolites. This approach uses the correlation of all mass signals with the targeted metabolite across samples to build networks that can be visualized in PDF or exported to Cytoscape. This can be a very useful approach to identify related compounds and associate them to metabolic pathways. Similarly, ProbMetab [129] is an R package for probabilistic annotation of compounds based on the method developed by Rogers et al. (2009) [130] that incorporates information on possible biochemical reactions between the candidate structures to assign higher probabilities to compounds that form substrate/product pairs within the same sample. Metabolite Identification Package (MI-Pack) [131], implemented in python, calculates differences in mass between all molecular formulas annotated from HRMS and compares them to known substrate/product pairs from KEGG, but matches are considered based on the error between experimental and theoretical masses compared to a threshold defined by a calculated mass error surface. Plant Metabolite Annotation Toolbox (PlantMAT) [132] is a particularly interesting tool designed specifically for the investigation of plant specialized metabolism, which uses an approach based on common metabolic building blocks to predict combinatorial possibilities of phytochemical structures used for annotation and as such is a highly effective way to search the chemical space surrounding a (set of) metabolite(s).

Another more recent and promising approach made possible by the huge amount of data available uses algorithms, mostly based on machine learning, to predict molecular properties of unknown compounds from their tandem mass spectra. All the tools listed below provide similar web interfaces for putative metabolite identification, differing mainly on the algorithms used to perform the identification and the overall performance. MetFrag [133] retrieves candidate structures either from databases based on exact mass or from user-specified structure-data files, a data format based on MDL Molfile, with a focus on caring structural information. Candidate structures are fragmented using a bond dissociation approach, and fragments are compared with the input spectra, scoring matches based on a series of rules. The candidates can also be filtered to facilitate the analysis based on relevant factors such as metabolite origin, composition, LC retention time, and metadata from the databases. Besides the Java web interface, a command line version and an R package are provided, which are more suitable for batch processing and integration with other tools. In a very similar approach, MolFind [134] retrieves candidates from databases based on exact mass, filters them by comparing an experimentally measured retention index, ECOM50 (the energy in eV required to fragment 50% of a selected precursor ion), and drift time (for ion mobility MS) with predicted ones, and analyzes CID of the best candidates using MetFrag. Competitive Fragmentation Modeling for Metabolite Identification (CFM-ID) [135] is based on competitive fragmentation modeling, a probabilistic generative model that uses machine learning to learn its parameters from data. It can be used to predict spectra of known chemical structures, to annotate peaks in the spectra of a known compound, or to predict candidate structures for an unknown compound by ranking candidates in terms of how closely the predicted spectra match the input. MS Annotation Based on In Silico Generated Metabolites (MAGMa) [136] extends prediction based on substructure assignment by creating hierarchical trees of predicted substructures capable of explaining MS^n^ data, where each level takes into account the restrictions imposed by the assignment of precursor and subsequent fragmentation. FingerId [137] developed a model based on a large dataset of tandem MS from MassBank and uses a support vector machine to predict the molecular fingerprint of the unknown spectra and compare this with the fingerprint of compounds in a large molecular database. CSI: FingerID [138] is a more recent tool based on fingerID that includes computation of a fragmentation tree, achieving 1 of the best search performances. Besides the web interface, it can be also queried directly through Sirius, but it currently does not support batch mode. CSI: IOKR was the last CASMI winner approach for the category “Best Automatic Structural Identification—*In Silico* Fragmentation Only” [116]. It is based on the integration of CSI: FingerID with an Input Output Kernel Regression (IOKR) machine learning approach to predict the candidate scores [139]. CSI: IOKR outperforms other approaches in metabolite identification rate while considerably shortening running time; nevertheless, it is still not available as an implemented workflow. Finally, MetFusion [140] is a Java web tool that combines spectra database matching against MassBank with the prediction-based annotation provided by MetFrag.

## Data Interpretation

Interpretation of omics data is usually complicated by the amount and complexity of data. There are many tools to assist metabolomics data interpretation, particularly for its visualization by mapping metabolites into pathways and providing biological context, and for the integration with data from different platforms (e.g., transcriptomics, proteomics; see Tohge et al. (2015) [15] for details). As for metabolite annotation, these tools usually rely upon knowledge stored in metabolite and pathway databases, and many of them include some kind of statistical analysis such as pathway enrichment and correlation analysis.

Visualization tools provide a simple means of representing and mapping metabolic changes in tools like PATHOS [141], PathWhiz [142], and Interactive Pathways Explorer (iPath) [143]. They can often provide some kind of pathway structure analysis such as PathVisio [144], Functional Enrichment Analysis Tool (FunRich) [145], BiNChE [146], and Metabolite Pathway Enrichment Analysis (MPEA) [147] that uses pathway enrichment analysis and pathway activity profiling [148] that calculates pathway activity scores to represent the potential metabolic pathway activities and performs statistical analysis to investigate differences in activity between conditions. Tools like Integrated Analysis of Cross-Platform Microarray and Pathway Data (InCroMAP) [149], Integrated Interactome System (IIS) [150], Kazusa Plant Pathway Viewer (KaPPA-View4) [151], MapMan [152], ProMeTra (which is integrated with MeltDB 2.0) [153], Paintomics [154], Visualization and Analysis of Networks Containing Experimental Data (VANTED) [155], MBROLE [156], and Integrated Molecular Pathway Level Analysis (IMPaLA) [157] go 1 step further and integrate metabolomics processed data with other omics platforms, particularly transcriptomics, providing analysis and visualization of large integrated datasets to assist data interpretation.

Few tools try to actually use mass spectra features to build the networks, which can also improve annotation of unknown compounds. MetaNetter [158] uses raw high-resolution data and a list of potential biochemical transformations to infer metabolic networks. MetaMapR [159] builds chemical and spectral similarity networks based on annotated and unknown compounds. ChemTreeMap [160] uses annotated structures and a computational approach to produce hierarchical trees based on compound similarity to assist visualization of chemical overlap between molecular datasets and the extraction of structure–activity relationships. MetFamily [161] groups metabolites into families based on an integrated analysis of MS1 abundances, with MS/MS facilitating further data interpretation. MetCirc [162] is an R tool that is particularly useful for comparative analysis from cross-species and cross-tissue experiments through computation of similarity between individual MS/MS spectra and visualization of similarity based on interactive graphical tools, and TrackSM [163] is a Java tool that uses molecular structure similarities to assign newly identified biochemical compounds to known metabolic pathways.

## Databases

It must be clear from previous sections that mass spectrometry–based metabolomics, particularly metabolite annotation and data interpretation, relies heavily upon data from characterized mass spectra, molecular properties of analytes, and metabolic pathways. While all the different techniques offer a lot of flexibility, metabolomics struggles with standardization, and a great volume of metadata when compared with other omics techniques and still lags behind most of them in terms of public repositories of published data. Nonetheless, there is a wealth of databases with useful information for mass spectrometry–based plant metabolomics, and we try to summarize some of the most relevant and the structure and functionalities of the resources available.

Chemspider [164], PubChem [165], Chemical Entities of Biological Interest (ChEBI) [166], ChEMBL [167], ChemBank [168], HMDB [169], MMCD [170], and MMsINC [171] are all large databases of small molecules with information such as chemical structure, molecular formula, and molecular/exact mass. Many of these databases complement each other, and data exchange between them is very common. Nevertheless, it is important to be aware of the sources of data in each 1 of them and to which extent these data are curated. Chemspider, for instance, has more than 58 million structures automatically retrieved from over 450 different sources, with only a fraction of this being manually curated by registered users while the majority of data only went through some sort of automatic curation and elimination of redundant entries. Overall, such huge databases are particularly useful for looking for physico-chemical properties of identified metabolites and checking for possible candidates based solely on their mass.

There are a few plant-specific databases with curated information on chemical composition and distribution across different plant species as well, namely KNApSAcK [172], with information on more than 50 000 metabolites and chemical composition of over 22 000 species, the Universal Natural Products Database (UNPD) [173], with a Flavonoid viewer of 229 358 metabolite structures [174] with 6902 molecular structures of flavonoids from 1687 plant species, Dr. Duke's Phytochemical and Ethnobotanical Databases [175], with information on 29 585 chemicals of 3686 medicinal plants, BioPhytMol [176], a resource on anti-mycobacterial phytomolecules and plant extracts holding 2582 entries including 188 plant families, comprised of 692 genera and 808 species and 633 active compounds and plant extracts identified against 25 target mycobacteria, and the Essential Oil Database (EssOilDB) [177], with 123 041 essential oil records from 92 plant families. These are very interesting resources for screening chemical composition of specific species and analyzing chemical distribution species-wide, and all of the data in these databases are manually curated. Of all these resources, KNApSAcK is particularly useful, not only for the large amount of data but also for providing an easy platform to access and extract information quickly.

Databases that provide mass spectra of pure compounds under controlled conditions developed to allow searching for common spectra features for the identification of unknown compounds are an essential resource for MS-based identification of metabolites. As previously mentioned, the great stability and reproducibility of GC-MS generates reliable fragmentation patterns and relative retention indexes that are very efficient for metabolite annotation by spectra matching. NIST is a very popular commercial library for GC-MS annotation that also provide free access to some data through NIST Chem WebBook [178], containing mass spectra of 33 000 compounds. Spectral Database for Organic Compounds (SDBS) [179], with 25 000 mass spectra, is the database of the National Institute of Advanced Industrial Science and Technology (AIST) from Japan. Both are limited in the fact that they do not offer an interface for spectra matching and the users have limited access to data, so they are only useful for checking the spectra of targeted compounds. Some more interesting freely accessible plant-specific GC-MS libraries include the Golm Metabolome Database [180], with a total of 26 590 spectra and 4663 analytes at the time this article was written, and the VocBinBase [181], which included 1537 unique mass spectra at the time this article was written. Both of these databases can be downloaded and integrated to processing tools for metabolite annotation based on spectra matching. Also worth mentioning is fiehnLib [182]; however, access to the spectral data is highly limited for this resource.

One of the greatest efforts in the field of metabolomics has been directed to the development of databases of mass spectra obtained from LC-MS analysis. The higher flexibility of this technique compared to GC-MS in terms of the chemical space that it can analyze comes with the drawback of a high sensitivity to multiple factors that can influence mass spectra quality and reproducibility. LC-MS databases are usually characterized by the greatest volume of metadata that accompany the analytical data and a more complex structure for search based on spectra features when compared to GC-MS databases. Some large general LC-MS databases include MassBank [183], a public repository of mass spectra with 41 092 spectra of 15 828 compounds obtained by 26 different systems (at the time of writing). This database is very accessible, allowing search by submitted spectra or simply by typing in spectral features, mass, or targeted compound name. It furthermore allows users to directly extract spectra during data processing through many tools like RAMClustR, RMassBank, and Mass^++^. Metabolite Link (METLIN) [184] currently contains 961 829 molecules, from which 200 000 have *in silico* MS/MS data. Additionally, over 14 000 metabolites were analyzed, and mass spectra at multiple collision energies in positive and negative ionization mode obtained. METLIN also integrates isoMETLIN [185], which allows the search of isotopologues for all METLIN metabolites based on m/z and isotopes of interest, and includes experimental data on hundreds of isotopic labeled metabolites that can be used to obtain information of precursor atoms in the fragments. Both databases can be accessed after free registration, and searching by mass is fast and easy, with the advantage that it allows the user to select possible adducts and spectra conditions and search directly the mass observed in the spectra. Toxin and Toxin Target Database (T3DB) [186] is a database for toxin data, many of which are plant secondary metabolites, with MS, MS-MS, and GC-MS spectra of 3600 common toxic substances (at the time of writing). mzCloud is a new database with a more complex organizing structure that can improve and facilitate data interpretation, currently with 6255 compounds analyzed in different conditions, totaling 1 913 621 spectra arranged in 9896 tree structures. It allows the user to easily navigate through different spectra of a single compound through its tree structure and includes visualization of the predicted molecular formula of the fragments in the spectra [187]. Finally, the recently developed MassBank of North America (MoNA) [188] is intended to be a centralized, collaborative database of metabolite mass spectra and metadata, currently containing over 200 000 mass spectral records from experimental and *in silico* libraries from different sources. The search is limited to name, compound class, molecular formula, or exact mass of the metabolite. It can be filtered by type of spectra, and the results are presented as a single list of individual interactive spectra next to the metadata, making it easy to navigate through different spectra. The great diversity of phytochemicals observed in plants represents an important portion of all these numbers, and a few plant-specific databases are available, such as Spektraris [189], an LC-MS of about 500 plant natural products that integrates accurate mass–time tag to incorporate retention time relative to an internal standard in a similar fashion, as is usually done for GC-MS-based annotation; therefore, in order to use this feature, it is necessary to analyze samples with the addition of the same internal standard used when developing the database entries. It is important to highlight that this kind of approach is much less effective for LC-MS, where relative retention time is prone to larger variation. MS-MS Fragment Viewer [190] is a very small and not very frequently updated database containing Fourier transform (FT)–MS, ion trap– (IT-), and FT-MS/MS spectral data on 116 flavonoids. RIKEN MSn Spectral Database for Phytochemicals (ReSpect) [191] is a collection of MSn spectra data from 9017 phytochemicals from the literature and standards with searching functionalities very similar to MassBank and WEIZMASS [192], a metabolite spectral library of high-resolution MS data from 3540 plant metabolites that uses a probabilistic approach to match library and experimental data with the MatchWeiz software. WEIZMASS is available for implementation in R as a pipeline for metabolite identification, which can be easily integrated with data processing. While this is a much less accessible tool for general use compared with other web-based databases, the results obtained are far more considerable and the effort required in its use is, therefore, more than compensation for the gains that it affords.

A very common issue encountered in data from mass spectrometry is the presence of a variety of contaminants from sample preparation and analysis that can be challenging for data interpretation. Mass Spectrometry Contaminant Database (MaConDa) [193] provides a very useful database of common contaminants and adducts in mass spectrometry, containing over 200 contaminant records with origin of the contaminant, its mass, and the adducts formed. MaConDa can be downloaded in different formats or accessed via a web browser.

Compound spectra databases are essential for identification of metabolites by mass spectrometry, but a significant effort has also been directed toward the development of repositories of experimental data on specific samples to facilitate dereplication studies and data analysis. These databases are often restricted to specific species, as is the case for AtMetExpress [194], an LC-MS database of Arabidopsis with data on 20 different ecotypes and 36 developmental stages that allows users to download raw and processed data as well as query using mass chromatogram features in the web platform and visualize annotation and distribution of selected features. Metabolite Profiling Database for Knock-Out Mutants in Arabidopsis (MeKO) [195] is a GC-MS database of 50 Arabidopsis KO mutants. All raw data can be downloaded as net common data format (CDF) files, and results from data analysis can be visualized in a very informative summary in the web browser that shows plant phenotypes, differentially accumulated metabolites indicated in a pathway map, and log fold changes for most significantly changed metabolites. MoTo DB [196] is an LC-MS database of *Solanum lycopersicum* with information on annotated metabolites where the user can search for specific masses or a range of masses. The database is based on accurate mass, and the user therefore does not have access to raw data and chromatograms. Nicotiana attenuata Data Hub (NaDH) [197], a platform for the integration and visualization of different omics datasets of *Nicotiana attenuata* including LC-MS data on 14 different tissues, allows searching for spectra based on name and m/z and provides some interesting tools for data interpretation that are easily accessible directly from the metabolite entry, including metabolite-metabolite and metabolite-gene coexpression analysis and visualization of metabolite expression across different tissues in a bar chart or eFP browser interface. Optimas-DW software [198] is a data collection for maize data of 15 different experiments. The interface for metabolites allows easy browsing through all the metabolites and visualization of values for individual experiments in a table format but no access to raw data. Soybean Metabolome Database (SoyMetDB) [199], a metabolomics database for soybeans, with GC-MS and LC-MS data of 4 different tissues under 2 different conditions, has a simple interface that provides search by metabolite name or browsing through the whole dataset. Metabolite entries provide m/z and retention time as well as an apparent defunct link to a pathway viewer. Similar databases with relative broader spectra include the plant-specific KOMIC Market [200], currently warehousing LC-MS data on 74 samples from 17 species, in which the user can search for peaks and browse through samples and the interface shows retention times, m/z, and annotation details classifying the annotation based on a grading system. MS/MS spectral tag (MS2T) [201] is an MSMS library created using a function for automatic Tandem MS acquisition from over 150 samples from 10 different plant species. The web platform allows search by retention time, m/z, and spectra similarity. Plant/Eukaryotic and Microbial Systems Resource (PMR) [202] is a database for plants and eukaryotic microorganisms that includes the earlier database of medicinal plants Medicinal Plant Metabolomic Resources (MPMR) [203] and currently comprises GC-MS and LC-MS data on 24 species from different sources and experiments including different tissues and developmental stages. It has an easy and clear interface, with a summary of all the experiments once an individual species is selected including metadata and annotated metabolites. It additionally allows the download of all the results in csv format in the form of peak tables, and it has some basic tools for comparative analysis where volcano plots can be generated comparing different experiments. By contrast, in the more general database Bio-MassBank [204], a repository of LC-MS and GC-MS data from biological samples, in contrast with the original MassBank in this database, most of the data are tagged as “Unknown” or are just putative metabolites. Searching functions are similar to the original database, but they include a samples section where it is possible to access all the experiments available. MassBase [205] is a large repository providing raw and processed mass chromatograms on 46 398 samples of over 40 species, including several plants, analyzed by LC-MS, GC-MS, and CE-MS. Metabolomics Workbench [206] is a repository of a variety of metabolomics experiments containing over 60 000 entries, including raw and processed MS data, a section with detailed protocols for the experiments, and web tools for analysis and interpretation that can be used with any uploaded data. Similarly, Metabolights [207] is a cross-species repository containing data from 190 mass spectrometry–based metabolomics studies that is currently recommended as repository of experimental data by many journals. All experimental data can be downloaded from a file transfer protocol server, and data submission is powered by the use of ISA software, which assists in the reporting and management of metadata. MetabolomeXchange [208] is a data aggregation system that allows users to efficiently explore experimental metabolomics data from different databases including MetaboLights and Metabolomics Workbench, providing a rich site summary feeding service to allow users to get updates over the datasets available. Similarly, Global Natural Products Social Molecular Networking (GNPS) [209], a plant natural product knowledge base for community-wide organization and sharing of raw, processed, or identified tandem mass spectrometry data, is currently comprised of 221 083 MS/MS spectra from 18 163 unique compounds. The platform allows users to upload data and provides a series of tools for analysis and interpretation based on the data from the database.

As previously mentioned, many resources that are particularly useful for data interpretation organize the data in pathways based on literature data, and often also provide tools for data visualization and interpretation. Many of these databases contain either generic pathways or combine different organisms. One example is KEGG [210], which includes 504 pathway maps with 17 891 compounds and 10 419 reactions for 4607 different organisms, representing data in an interactive interface that links the entries to a great amount of external resources, and being 1 of the most popular sources of information on metabolic pathways. One of the greatest issues of KEGG leading many users to misinterpreting their data is that it displays all genes in generic pathway maps, of which some are characterized only by similarity, resulting in pathways that are not present in the analyzed organism being represented. By contrast, WikiPathways [211] is a wiki-style website with 2471 community-curated pathways of 28 different organisms. Its interactive interface is similar to KEGG, providing links with external resources for metabolites and enzymes. Similarly, Khaos Metabolic Pathways (kpath) [212] is a database that integrates information related to metabolic pathways with 74 180 pathways, 13 153 reactions, and 37 029 metabolites providing tools for pathway visualization, editing, and relationship search. BioCyc [213] is a collection of 9387 pathway/genome databases, and MetaCyc [213] is the largest curated database of experimentally elucidated metabolic pathways, containing 2491 pathways from 2816 different organisms. KBase [214], meanwhile, is a data platform with data on plants and microbes that allows users to upload their own data and integrates data and tools for systems biology including 1470 metabolic pathways with 33 773 reactions and 27 838 compounds, genome data on 60 different plant species, and tools for assembly, annotation, metabolic modeling, comparative analysis, phylogenetic analysis, and expression analysis. There is also a significant amount of plant-specific data organized in databases like KaPPA-View4 [151], containing 153 pathways with 1427 compounds and 1434 reaction from 10 species, allowing users to upload their own data. It is able to represent gene-to-gene and metabolite-to-metabolite relationships as curves on metabolic pathway maps to help in data interpretation. PlantCyc [215] provides access to manually curated or reviewed information about metabolic pathways in over 800 pathways of 350 plant species. Usefully, the platform provides “evidence codes” to clearly indicate the type of support associated with each database item. MetaCrop [216] is a pathway database containing information about 7 major crop plants and 2 model plants that allows integration of experimental data into metabolic pathways, as well as the automatic export of information for the creation of detailed metabolic models. Similarly, Metabolic Network Exchange Database (MetNetDB) [217] contains integrative information on metabolic and regulatory networks of Arabidopsis and soybeans with metabolism, signaling, and transcriptional pathways being fully integrated into a single network, and manually curated subcellular localization is represented in the pathway maps. The network information can be exported to other applications for network analysis, such as exploRase and Cytoscape/FCM. Like MetNetDB, Gramene [218] is an integrated data resource for comparative functional genomics in crops and model plants that hosts pathway databases for rice, maize, Brachypodium, and sorghum, as well as providing mirrors for MetaCyc and PlantCyc data. It is worth mentioning a few resources that are focused on the reactions within the pathways offering detailed curated metabolic reactions, namely BioMeta [219], whose contents are based on the KEGG Ligand database with a large number of chemical structures corrected with respect to constitution and reactions’ stereochemistry being correctly balanced. BRENDA-KEGG-MetaCyc reactions (BKM-react) [220] is a non-redundant biochemical reaction database containing 18 172 unique biochemical reactions retrieved from BRENDA, KEGG, and MetaCyc databases that were matched and integrated by aligning substrates and products. Similar to this, MetRxn [221] also integrates information from BRENDA, KEGG, and MetaCyc, combining also Reactome.org and 44 metabolic models in a standardized description of metabolites and reactions where all metabolites have matched synonyms, resolved protonation states, and are linked to unique structures, and all reactions are balanced.

Together with the development of many prediction tools previously mentioned, we watched in the last years the development of some interesting *in silico* databases that are extremely useful for *de novo* metabolite identification, such as Metabolic *In Silico* Network Expansion Databases (MINE) [222], a database developed by the integration of an algorithm called the Biochemical Network Integrated Computational Explorer (BNICE), and expert-curated reaction rules to predict chemical structures’ product of enzyme promiscuity, Metabolite Collision Cross-Section Predictor (MetCCS) [223], a database and algorithm for prediction of collision cross-section values for metabolites in ion mobility mass spectrometry, a technique increasingly used to assist metabolite elucidation based on the drift speed of the ion that is proportional to its cross-section, and the plant-specific *In Silico* MS/MS Database (ISDB) [224], an *in silico* database of natural products generated using CFM-ID [135] with input from the commercial Dictionary of Natural Products.

## Other Programs of Interest

The complexity of metabolomics data experiments, particularly in terms of sample number and metadata, pushed the development of many tools for experiment and metadata management, and while many of these functions are integrated in some of the databases previously discussed, there are a few specialized tools such as QTREDS [225] and MASTR-MS [226] that are Laboratory Information Management System (LIMS)–based software for assisting in organizing experimental design, metadata management, and sample data acquisition. MetaDB [227] is a web application for metabolomics metadata management with interface to the MetaMS data processing tool, and Metabolonote [228] is a metadata database/management system.

The enormous amount of data available for metabolomics raises many questions regarding how to easily access and unify all this data, taking into account the vast chemical space explored in these experiments. Many tools have been developed with the purpose of facilitating access to chemical data spread in the literature, from the development of identifiers to reduce duplication of information such as Spectral Hash [229], designed for the MoNA database, to tools like Metmask [230] for managing different identifiers, Chemical Translation Service (CTS) [231] for translation of chemical identifiers, PhenoMeter [232] for querying databases based on metabolic phenotype, and Metab2MeSH [233] for a more efficient literature search that automatically annotates compounds with the concepts defined in MeSH, providing a fast link between the compound and the literature.

Different vendors usually export their data in proprietary formats, which complicates data transfer across different platforms. Most proprietary software packages are able to convert files to .cdf format, but some tools, the most popular being msConverter from Proteowizard [234], can handle conversion from/to different formats including mzXML. mzTab is another format proposed by the Proteomics Standards Initiative targeting researchers outside of proteomics. It is supposed to contain the minimal information required to evaluate the results of a proteomics experiment, making it more accessible to non-experts. jmzTab [235] is a Java application that provides reading and writing capabilities and conversion of files to mzTab. The PeakML [236] file format is an initiative developed by the creators of mzMatch to enable the exchange of data between analysis software by representing peak and meta-information from each step in an analysis pipeline; as a proof of concept, the R-package “mzmatch.R” was developed to extend XCMS functionalities for storing and reading data in PeakML format.

All equipment for mass spectrometry comes with its own software for data visualization and some basic analysis, but those are usually not designed to deal with the complexities of metabolomics datasets. There are some interesting open source alternatives such as BatMass [237] and Mass^++^ [238] for data visualization, and for generating images from raw data like SpeckTackle [239], which provides several pre-defined chart types that are easy to integrate into web-facing resources, and RMassBank [240], capable of automatically generating MassBank records from raw MS and MS/MS data.

Mass spectrometry imaging is a relatively young technique that has being growing fast in importance, providing high-resolution special distribution of small molecules in molecular histology [241]. Few tools have been developed so far, namely Exploring Imaging Mass Spectrometry Data (EXIMS) [242] for data processing and analysis and Open Mass Spectrometry Imaging (OpenMSI) [243], a web-based visualization, analysis, and management tool.

Lipidomics data require a very specialized pipeline, and therefore many tools were developed exclusively for this kind of analysis; however, we will only briefly summarize these here. Analysis of Lipid Experiments (ALEX) [244], Multiple Reaction Monitoring–Based Differential Analysis (MRM-DIFF) [245], LICRE [246], LipidXplorer [247], Lipid Mass Spectrum Analysis (LIMSA) [248], Visualization and Phospholipid Identification (VaLID) [249], Lipid and Oxylipin Biomarker Screening Through Adduct Hierarchy Sequences (LOBSTAHS) [250], Lipid-Pro [251], lipid data analyzer (LDA) [252], and LipidQA [253] are all tools for processing, annotating, and analyzing lipidomics data. Lipids databases include LIPID MAPS [254], LIPIDBANK [255], LipidBlast [256], and *in silico* generated lipids databases LipidHome [257], SwissLipids [258] ,and ARALIP [259].

## Future Perspectives

Many of the resources presented here were fruit of the efforts of setting the theoretical background for each step in the data processing and analysis workflow. However, more recent efforts are moving toward the development of integrated tools, which are often developed by the integration of already well-established tools into a single pipeline in an attempt to accelerate the process and in a few cases providing an easier interface. XCMS online, for example, is a web platform providing most of the function from XCMS with additional capabilities for interactive exploratory data visualization and analysis in a much easier interface than the original software [260]. HayStack [261] is a web platform that uses XCMS to process data and automatically generates total ion current chromatograms and base peak chromatograms as well as offering an easy way of plotting extracted ion chromatograms (EIC) and some basic statistical tools such as PCA scores plot, volcano plots, and dendrograms for group comparisons. Statistical Metabolomics Analysis–An R Tool (SMART) [262] is an R package that combines different tools such as XCMS and CAMERA with a series of common statistical approaches to provide an integrated pipeline for data processing, visualization, and analysis. MZmine 2 [263] is another very popular tool, with over 1000 citations. It was originally developed for LC-MS data processing, but it became 1 of the most popular platforms for development of integrated tools in Java, providing a user-friendly, flexible, and extendable software constantly updated and with a set of modules covering most steps of LC-MS processing and data analysis workflow, including several option of visualization tools. MetSign [264] is a MATLAB package providing tools for spectra deconvolution, metabolite putative assignment by matching m/z, and peak isotopic distribution against its own database, peak list alignment, a series of normalization algorithms, statistical significance tests, unsupervised clustering, and time course analysis, all in a modular and interactive design presented with a wizard to facilitate the analysis workflow. MultiAlign [265] is a software developed in the .NET platform using C++ and C# that was originally for proteomics but that can also be used for metabolomics comparative analysis. Its functionalities include feature detection, alignment, several plotting options, normalization, and basic statistical comparisons, Metabolome Express [266] works as a web server to process, interpret, and share GC/MS metabolomics datasets, whilst Metabolite Automatic Identification Toolkit (MAIT) [267] is an R package aimed at providing an end-to-end programmable metabolomics pipeline with an emphasis on metabolite annotation and statistics. It uses XCMS for peak detection, an approach based on CAMERA combined with a user-defined table of biotransformations, followed by database search for metabolite annotation and a series of statistical tests to identify statistically significant features containing the highest amount of class-related information. By contrast, Metabolomic Analysis and Visualization Engine (MAVEN) [268] is a software for data processing, analysis, and visualization with some interesting features for pathway-based visualization of isotope-labeling data that can be helpful for the interpretation of this kind of experiment. MeltDB [269] is a Java web-based platform that integrates different algorithms for data processing and compound identification by spectra matching statistical analysis, data visualization, and integration with transcriptomics and proteomics datasets via the ProMeTra software. It provides a tool for saving peaks of reference compounds directly in the MeltDB database and allows storage and sharing of projects within the web server. MetaboAnalyst [270] is another Java web platform with data processing and a comprehensive set of data analysis tools. It includes most common approaches for statistical analysis as well as modules for functional enrichment analysis, metabolic pathway analysis, time series and two-factor data analysis, biomarker analysis, sample size and power analysis, integrated pathway analysis, and image and report generation. The program mzMatch [236] is a popular Java toolkit for processing, filtering, and annotation, with a particular focus on integration of processed data across different platforms and providing a customizable modular pipeline to facilitate the development and integration of different tools. It includes many other tools previously described here like mzmatchISO and metAssign, and it is based entirely in the PeakML file format. The Marker Visualization Suite (MarVis-Suite) [271] is a software for the interactive ranking, filtering, combination, clustering, visualization, and functional analysis of transcriptomics and metabolomics datasets. The clustering algorithm is based on 1-dimensional self-organizing maps, and the software additionally provides functions for metabolite annotation and pathway reconstruction. MetMSLine [272] is an R package that works with processed data providing a series of statistical analysis steps focusing on biomarker discovery combined with metabolite annotation based on exact mass matching against a target list of metabolites, and MassCascade [273] is a Java library that takes advantage of the KINIME workflow environment, facilitating integration with other tools and making the tool user-friendly. The core library contains a collection of data processing algorithms, a visualization framework, and metabolite annotation functions, while the plug-in for KNIME allows easy integration with other statistical workflows. MASSyPup [274] does not actually integrate different procedures, but it does provide an easy platform for accessing many different tools in the form of a Linux distribution that can be run directly from different media without installation.

It is clear from this review the infinity of choices for performing a variety of functions and the fast pace by which they change and get outdated; hence it is an arduous task to keep updated on all of them. Some research groups, engaged in the development of metabolomics tools, have their own repositories like KOMICS [275], MetaOpen [276], and Platform for RIKEN Metabolomics (PRIMe) [277], while OMICtools [278], NAR online Molecular Biology Database Collection, and the Bioinformatics Links Directory provide unified repositories that cover only a small portion of all the resources available. Tools developed for R have the advantage of counting with some well-established platforms such as Biocunductor [279] or Comprehensive R Archive Network (CRAN). Nevertheless, with the rapid development of new tools, it is of great interest for the metabolomics community to develop classification systems and repositories to catalog and provide a platform for submission, curation, and feedback, facilitating users’ access to the most appropriate and updated resources for each aim. Another clear observation that can be made from the proceeding sections is that the number of tools for analysis by far exceeds that of the number of data repositories whilst metabolomics is clearly difficult to fully standardize. This is still a great shame. There are many clear reporting standards that should aid in this respect [280]; furthermore, both the existing databases and carefully compared meta-analyses [22, 281], demonstrate that such approaches are indeed highly powerful in the enhancement of biological understanding. As such, we feel that it is an urgent priority to focus efforts on the improvement of this feature of computational metabolomics since it will aid not only in the expansion of our coverage of the metabolite complement of the plant cell but also in the equally important task of interpreting the biological function of the individual metabolites themselves.

### Additional file

Additional file 1.xls: summary of resources for mass spectrometry–based metabolomics.

### Abbreviations

ADAP: Automated Data Analysis Pipeline for Untargeted Metabolomics; AIST: National Institute of Advanced Industrial Science and Technology; ALEX: Analysis of Lipid Experiments; AMDIS: Automated Mass Spectral Deconvolution And Identification System; ANOVA: analysis of variance; apLCMS: Adaptive Processing of High-Resolution LC-MS data; ARALIP: Arabidopsis acyl-lipid metabolism; ASCII: American Standard Code for Information Interchange; BKM-react: BRENDA-KEGG-MetaCyc reactions; BNICE: Biochemical Network Integrated Computational Explorer; CAMERA: Collection of Algorithms for Metabolite Profile Annotation; CDF: common data format; CFM-ID: Competitive Fragmentation Modeling for Metabolite Identification; ChEBI: Chemical Entities of Biological Interest; CID: collision-induced dissociation; cosmiq: combining single masses into quantities; COVAIN: Covariance Inverse; CRAN: Comprehensive R Archive Network; CSI: FingerID: compound structure identification: FingerID; CTS: Chemical Translation Service; DIA: data-independent acquisition; EIC: extracted ion chromatogram; EssOilDB: Essential Oil Database; EXIMS: Exploring Imaging Mass Spectrometry Data; FT: Fourier transform; FunRich: Functional Enrichment Analysis Tool; GC: gas chromatography; GNPS: Global Natural Products Social Molecular Networking; GUI: graphical user interface; HCS: hierarchical cluster analysis; HMDB: Human Metabolome Database; HRMS: high-resolution mass spectrometry; ICT: Isotope Correction Toolbox; IIS: Integrated Interactome System; iMet-Q: Intelligent Metabolomic Quantitation; IMPaLA: Integrated Molecular Pathway Level Analysis; InCroMAP: Integrated Analysis of Cross-Platform Microarray and Pathway Data; IOKR: Input Output Kernel Regression; iPATH: Interactive Pathways Explorer; IPO: Isotopologue Parameter Optimization; ISDB: *In Silico* MS/MS Database; KaPPA–view: Kazusa Plant Pathway Viewer; KEGG: Kyoto Encyclopedia of Genes and Genomes; KMMDA: Kernel Machine Approach for Differential Expression Analysis of Mass Spectrometry–Based Metabolomics Data; Komic Market: Kazusa Omics Data Market; kpath: Khaos Metabolic Pathways; LC: liquid chromatography; LDA: latent Dirichlet allocation; LDA: lipid data analyzer; LIMS: Laboratory Information Management System; LIMSA: Lipid Mass Spectrum Analysis; LOBSTAHS: Lipid and Oxylipin Biomarker Screening Through Adduct Hierarchy Sequences; m/z: mass-to-charge ratio; MaConDa: Mass Spectrometry Contaminant Database; MAGMa: MS Annotation Based on *In Silico* Generated Metabolites; MAIT: Metabolite Automatic Identification Toolkit; MarVis-Suite: Marker Visualization Suite; MathDAMP: Mathematica Package for Differential Analysis of Metabolite Profiles; MAVEN: Metabolomic Analysis and Visualization Engine; MeKO: Metabolite Profiling Database for Knock-Out Mutants in Arabidopsis; MetCCS: Metabolite Collision Cross-Section Predictor; MET-COFEA: Metabolite Compound Feature Extraction and Annotation; MET-COFEI: Metabolite Compound Feature Extraction and Identification; MET-IDEA: Metabolomics Ion-Based Data Extraction Algorithm; METLIN: Metabolite Link; MetNetDB: Metabolic Network Exchange Database; MFSearcher: Molecular Formula Searcher; MIA: Mass Isotopolome Analyzer; MID: mass isotopomer distributions; MINE: Metabolic *In Silico* Network Expansion Databases; MI-Pack: Metabolite Identification Package; MMCD: Madison Metabolomics Consortium Database; MMSAT: Metabolite Mass Spectrometry Analysis Tool; MoNA: MassBank of North America; MPA-RF: Model Population Analysis–Random Forests; MPEA: Metabolite Pathway Enrichment Analysis; MPMR: Medicinal Plant Metabolomic Resources; MRM: multiple reaction monitoring; MRM-DIFF: Multiple Reaction Monitoring–Based Differential Analysis; MRMPROBS: Multiple Reaction Monitoring–Based Probabilistic System; MS: mass spectrometry; MS/MS: tandem mass spectrometry; MS2T: MS/MS spectral tag; MS-DIAL: Mass Spectrometry–Data Independent Analysis; MSFACT: Metabolomics Spectral Formatting, Alignment, and Conversion Tool; MUSCLE: Multi-Platform Unbiased Optimization of Spectrometry via Closed-Loop Experimentation; NaDH: Nicotiana attenuata Data Hub; NIST: National Institute of Standards and Technology; OpenMSI: Open Mass Spectrometry Imaging; PCA: principal component analysis; PlantMAT: Plant Metabolite Annotation Toolbox; PLS-DA: partial least squares discriminant analysis; PMR: Plant/Eukaryotic and Microbial Systems Resource; PRIMe: Platform for RIKEN Metabolomics; RAMSY: Ratio Analysis of Mass Spectrometry; ReSpect: RIKEN MSn Spectral Database for Phytochemicals; SDBS: Spectral Database for Organic Compounds; SIRIUS: Sum Formula Identification by Ranking Isotope Patterns Using Mass Spectrometry; SMART: Statistical Metabolomics Analysis–An R Tool; SoyMetDB: Soybean Metabolome Database; SPICA: Selective Paired Ion Contrast; SPLASH: Spectral Hash; T3DB: Toxin and Toxin Target Database; UNPD: Universal Natural Product Database; VaLID: Visualization and Phospholipid Identification; VANTED: Visualization and Analysis of Networks Containing Experimental Data; yamss: Yet Another Mass Spectrometry Software.

## Supplementary Material

GIGA-D-17-00039_Original_Submission.pdf

GIGA-D-17-00039_Revision-1.pdf

GIGA-D-17-00039_Revision-2.pdf

Response_to_Reviewer_Comments_Original-Submission.pdf

Reviewer_1_Report_(Original-Submission).pdf

Reviewer_1_Report_(Revision-1).pdf

Reviewer_2_Report_(Original_Submission).pdf

Additional File

## References

[1] Oliver SG, Winson MK, Kell DB Systematic functional analysis of the yeast genome. Trends Biotechnol 1998;16(9):373–8. 9744112 10.1016/s0167-7799(98)01214-1

[2] Fiehn O, Kopka J, Dormann P Metabolite profiling for plant functional genomics. Nat Biotechnol 2000;18(11):1157–61. 11062433 10.1038/81137

[3] Sauter H, Lauer M, Fritsch H Metabolic profiling of plants - a new diagnostic-technique. Abstr Pap Am Chem Soc 1988;195:129-AGRO.

[4] Dorr JR, Yu Y, Milanovic M Synthetic lethal metabolic targeting of cellular senescence in cancer therapy. Nature 2013;501(7467):421–5. 23945590 10.1038/nature12437

[5] Kell DB Metabolomics and systems biology: making sense of the soup. Curr Opin Microbiol 2004;7(3):296–307. 15196499 10.1016/j.mib.2004.04.012

[6] Nicholson JK, Wilson ID Understanding ‘global’ systems biology: metabonomics and the continuum of metabolism. Nat Rev Drug Discov 2003;2(8):668–76. 12904817 10.1038/nrd1157

[7] Fernie AR, Schauer N Metabolomics-assisted breeding: a viable option for crop improvement? Trends Genet 2009;25(1):39–48. 19027981 10.1016/j.tig.2008.10.010

[8] Meyer RC, Steinfath M, Lisec J The metabolic signature related to high plant growth rate in Arabidopsis thaliana. Proc Natl Acad Sci U S A 2007;104(11):4759–64. 17360597 10.1073/pnas.0609709104 PMC1810331

[9] Roessner U, Willmitzer L, Fernie AR Metabolic profiling and biochemical phenotyping of plant systems. Plant Cell Rep 2002;21(3):189–96.

[10] Schauer N, Fernie AR Plant metabolomics: towards biological function and mechanism. Trends Plant Sci 2006;11(10):508–16. 16949327 10.1016/j.tplants.2006.08.007

[11] Weckwerth W Metabolomics in systems biology. Annu Rev Plant Biol 2003;54:669–89. 14503007 10.1146/annurev.arplant.54.031902.135014

[12] Fernie AR, Stitt M On the discordance of metabolomics with proteomics and transcriptomics: coping with increasing complexity in logic, chemistry, and network interactions. Plant Physiol 2012;158(3):1139–45. 22253257 10.1104/pp.112.193235 PMC3291261

[13] Nobeli I, Ponstingl H, Krissinel EB A structure-based anatomy of the E-coli metabolome. J Mol Biol 2003;334(4):697–719. 14636597 10.1016/j.jmb.2003.10.008

[14] van der Werf MJ, Overkamp KM, Muilwijk B Microbial metabolomics: toward a platform with full metabolome coverage. Anal Biochem 2007;370(1):17–25. 17765195 10.1016/j.ab.2007.07.022

[15] Tohge T, Scossa F, Fernie AR Integrative approaches to enhance understanding of plant metabolic pathway structure and regulation. Plant Physiol 2015;169(3):1499–511. 26371234 10.1104/pp.15.01006 PMC4634077

[16] Sulpice R, Pyl E-T, Ishihara H Starch as a major integrator in the regulation of plant growth. Proc Natl Acad Sci U S A 2009;106(25):10348–53. 19506259 10.1073/pnas.0903478106 PMC2693182

[17] Davey MP, Burrell MM, Woodward FI Population-specific metabolic phenotypes of Arabidopsis lyrata ssp. petraea. New Phytologist 2008;177(2):380–8. 18028292 10.1111/j.1469-8137.2007.02282.x

[18] Beleggia R, Rau D, Laidò G Evolutionary metabolomics reveals domestication-associated changes in tetraploid wheat kernels. Mol Biol Evol 2016;33(7):1740–53. 27189559 10.1093/molbev/msw050 PMC4915355

[19] Kliebenstein D Advancing genetic theory and application by metabolic quantitative trait loci analysis. Plant Cell 2009;21(6):1637–46. 19525414 10.1105/tpc.109.067611 PMC2714920

[20] Luo J Metabolite-based genome-wide association studies in plants. Curr Opin Plant Biol 2015;24:31–8. 25637954 10.1016/j.pbi.2015.01.006

[21] Brotman Y, Landau U, Pnini S The LysM receptor-like kinase LysM RLK1 is required to activate defense and abiotic-stress responses induced by overexpression of fungal chitinases in Arabidopsis plants. Mol Plant 2012;5(5):1113–24. 22461667 10.1093/mp/sss021

[22] Obata T, Fernie AR The use of metabolomics to dissect plant responses to abiotic stresses. Cell Mol Life Sci 2012;69(19):3225–43. 22885821 10.1007/s00018-012-1091-5 PMC3437017

[23] Tohge T, Fernie AR Web-based resources for mass-spectrometry-based metabolomics: a user's guide. Phytochemistry 2009;70(4):450–6. 19285697 10.1016/j.phytochem.2009.02.004

[24] Hibbert DB Experimental design in chromatography: a tutorial review. J Chromatogr B Analyt Technol Biomed Life Sci. 2012;910:2–13. 10.1016/j.jchromb.2012.01.020 22333438

[25] Gullberg J, Jonsson P, Nordström A Design of experiments: an efficient strategy to identify factors influencing extraction and derivatization of Arabidopsis thaliana samples in metabolomic studies with gas chromatography/mass spectrometry. Anal Biochem 2004;331(2):283–95. 15265734 10.1016/j.ab.2004.04.037

[26] Nistor I, Cao M, Debrus B Application of a new optimization strategy for the separation of tertiary alkaloids extracted from Strychnos usambarensis leaves. J Pharmaceut Biomed Anal 2011;56(1):30–7. 10.1016/j.jpba.2011.04.027 21628087

[27] Bradbury J, Genta-Jouve G, Allwood JW MUSCLE: automated multi-objective evolutionary optimization of targeted LC-MS/MS analysis. Bioinformatics 2015;31(6):975–7. 25388146 10.1093/bioinformatics/btu740 PMC4380028

[28] Nikolskiy I, Siuzdak G, Patti GJ Discriminating precursors of common fragments for large-scale metabolite profiling by triple quadrupole mass spectrometry. Bioinformatics 2015;31(12):2017–23. 25691443 10.1093/bioinformatics/btv085 PMC4481697

[29] Katajamaa M, Orešič M Data processing for mass spectrometry-based metabolomics. J Chromatography A 2007;1158(1–2):318–28. 10.1016/j.chroma.2007.04.021 17466315

[30] Sugimoto M, Kawakami M, Robert M Bioinformatics tools for mass spectroscopy-based metabolomic data processing and analysis. Curr Bioinformatics 2012;7(1):96–108. 10.2174/157489312799304431 PMC3299976 22438836

[31] Lange E, Tautenhahn R, Neumann S Critical assessment of alignment procedures for LC-MS proteomics and metabolomics measurements. BMC Bioinformatics 2008;9:375. 18793413 10.1186/1471-2105-9-375 PMC2570366

[32] Tautenhahn R, Böttcher C, Neumann S Highly sensitive feature detection for high resolution LC/MS. BMC Bioinformatics 2008;9(1):504. 19040729 10.1186/1471-2105-9-504 PMC2639432

[33] Lommen A MetAlign: interface-driven, versatile metabolomics tool for hyphenated full-scan mass spectrometry data preprocessing. Anal Chem 2009;81(8):3079–86. 19301908 10.1021/ac900036d

[34] Smith CA, Want EJ, O’Maille G XCMS: processing mass spectrometry data for metabolite profiling using nonlinear peak alignment, matching, and identification. Anal Chem 2006;78. 10.1021/ac051437y 16448051

[35] Tengstrand E, Lindberg J, Åberg KM TracMass 2: a modular suite of tools for processing chromatography-full scan mass spectrometry data. Anal Chem 2014;86(7):3435–42. 24611572 10.1021/ac403905h

[36] Chang H-Y, Chen C-T, Lih TM iMet-Q: a user-friendly tool for label-free metabolomics quantitation using dynamic peak-width determination. PLoS One 2016;11(1):e0146112. 26784691 10.1371/journal.pone.0146112 PMC4718670

[37] Treviño V, Yañez-Garza IL, Rodriguez-López CE GridMass: a fast two-dimensional feature detection method for LC/MS. J Mass Spectrom 2015;50(1):165–74. 25601689 10.1002/jms.3512

[38] Duran AL, Yang J, Wang L Metabolomics spectral formatting, alignment and conversion tools (MSFACTs). Bioinformatics 2003;19(17):2283–93. 14630657 10.1093/bioinformatics/btg315

[39] Broeckling CD, Reddy IR, Duran AL MET-IDEA: data extraction tool for mass spectrometry-based metabolomics. Anal Chem 2006;78(13):4334–41. 16808440 10.1021/ac0521596

[40] Fructuoso S, Sevilla Á, Bernal C EasyLCMS: an asynchronous web application for the automated quantification of LC-MS data. BMC Res Notes 2012;5(1):428. 22884039 10.1186/1756-0500-5-428 PMC3494514

[41] Creek DJ, Jankevics A, Burgess KE IDEOM: an Excel interface for analysis of LC–MS-based metabolomics data. Bioinformatics 2012;28(7):1048–9. 22308147 10.1093/bioinformatics/bts069

[42] Conley CJ, Smith R, Torgrip RJ Massifquant: open-source Kalman filter-based XC-MS isotope trace feature detection. Bioinformatics 2014;30(18):2636–43. 24872423 10.1093/bioinformatics/btu359

[43] Zhang W, Chang J, Lei Z MET-COFEA: a liquid chromatography/mass spectrometry data processing platform for metabolite compound feature extraction and annotation. Anal Chem 2014;86(13):6245–53. 24856452 10.1021/ac501162k

[44] Zhang W, Lei Z, Huhman D MET-XAlign: a metabolite cross-alignment tool for LC/MS-based comparative metabolomics. Anal Chem 2015;87(18):9114–9. 26247233 10.1021/acs.analchem.5b01324

[45] Yu T, Park Y, Johnson JM apLCMS—adaptive processing of high-resolution LC/MS data. Bioinformatics 2009;25(15):1930–6. 19414529 10.1093/bioinformatics/btp291 PMC2712336

[46] Uppal K, Soltow QA, Strobel FH xMSanalyzer: automated pipeline for improved feature detection and downstream analysis of large-scale, non-targeted metabolomics data. BMC Bioinformatics 2013;14(1):15. 23323971 10.1186/1471-2105-14-15 PMC3562220

[47] Myint L, Kleensang A, Zhao L Joint bounding of peaks across samples improves differential analysis in mass spectrometry-based metabolomics. Anal Chem 2017; DOI: 10.1021/acs.analchem.6b04719. PMC5362739 28221771

[48] Wandy J, Daly R, Breitling R Incorporating peak grouping information for alignment of multiple liquid chromatography-mass spectrometry datasets. Bioinformatics 2015;31(12):1999–2006. 25649621 10.1093/bioinformatics/btv072 PMC4760236

[49] Wehrens R, Bloemberg TG, Eilers PH Fast parametric time warping of peak lists. Bioinformatics 2015;31(18):3063–5. 25971741 10.1093/bioinformatics/btv299

[50] http://www.bioconductor.org/packages/devel/bioc/html/cosmiq.html (15 June 2017, date last accessed).

[51] Stein SE. An integrated method for spectrum extraction and compound identification from gas chromatography/mass spectrometry data. J Am Soc Mass Spectrom 1999;10(8):770–81.

[52] Aggio R, Villas SG, Ruggiero K Metab: an R package for high-throughput analysis of metabolomics data generated by GC-MS. Bioinformatics 2011;27(16):2316–8. 21697128 10.1093/bioinformatics/btr379

[53] Bunk B, Kucklick M, Jonas R MetaQuant: a tool for the automatic quantification of GC/MS-based metabolome data. Bioinformatics 2006;22(23):2962–5. 17046977 10.1093/bioinformatics/btl526

[54] Hiller K, Hangebrauk J, Jäger C MetaboliteDetector: comprehensive analysis tool for targeted and nontargeted GC/MS based metabolome analysis. Anal Chem 2009;81(9):3429–39. 19358599 10.1021/ac802689c

[55] Luedemann A, Strassburg K, Erban A TagFinder for the quantitative analysis of gas chromatography—mass spectrometry (GC-MS)-based metabolite profiling experiments. Bioinformatics 2008;24(5):732–7. 18204057 10.1093/bioinformatics/btn023

[56] Cuadros-Inostroza Á, Caldana C, Redestig H TargetSearch-a Bioconductor package for the efficient preprocessing of GC-MS metabolite profiling data. BMC Bioinformatics 2009;10(1):428. 20015393 10.1186/1471-2105-10-428 PMC3087348

[57] O’Callaghan S, De Souza DP, Isaac A PyMS: a Python toolkit for processing of gas chromatography-mass spectrometry (GC-MS) data. Application and comparative study of selected tools. BMC Bioinformatics 2012;13(1):115. 22647087 10.1186/1471-2105-13-115 PMC3533878

[58] http://bioinfo.noble.org/manuscript-support/met-cofei/ (15 June 2017, date last accessed).

[59] Jellema RH, Krishnan S, Hendriks MM Deconvolution using signal segmentation. Chemom Intell Lab Syst 2010;104(1):132–9.

[60] Wehrens R, Weingart G, Mattivi F metaMS: an open-source pipeline for GC–MS-based untargeted metabolomics. J Chromatogr B Analyt Technol Life Sci 2014;966:109–16. 10.1016/j.jchromb.2014.02.051 24656939

[61] Kuich PHJ, Hoffmann N, Kempa S Maui-VIA: a user-friendly software for visual identification, alignment, correction, and quantification of gas chromatography–mass spectrometry data. Front Bioeng Biotechnol 2014;2. 10.3389/fbioe.2014.00084 PMC4301187 25654076

[62] Domingo-Almenara X, Brezmes J, Vinaixa M eRah: a computational tool integrating spectral deconvolution and alignment with quantification and identification of metabolites in GC/MS-based metabolomics. Anal Chem 2016;88(19):9821–9. 27584001 10.1021/acs.analchem.6b02927

[63] Ni Y, Su M, Qiu Y ADAP-GC 3.0: improved peak detection and deconvolution of co-eluting metabolites from GC/TOF-MS data for metabolomics studies. Anal Chem 2016;88(17):8802–11. 27461032 10.1021/acs.analchem.6b02222 PMC5544921

[64] Wei X, Shi X, Koo I MetPP: a computational platform for comprehensive two-dimensional gas chromatography time-of-flight mass spectrometry-based metabolomics. Bioinformatics 2013;29(14):1786–92. 23665844 10.1093/bioinformatics/btt275 PMC3702250

[65] Kuhl C, Tautenhahn R, Böttcher C CAMERA: an integrated strategy for compound spectra extraction and annotation of liquid chromatography/mass spectrometry data sets. Anal Chem 2012;84(1):283–9. 22111785 10.1021/ac202450g PMC3658281

[66] Alonso A, Julià A, Beltran A AStream: an R package for annotating LC/MS metabolomic data. Bioinformatics 2011;27(9):1339–40. 21414990 10.1093/bioinformatics/btr138

[67] Kessler N, Walter F, Persicke M Allocator: an interactive web platform for the analysis of metabolomic LC-ESI-MS datasets, enabling semi-automated, user-revised compound annotation and mass isotopomer ratio analysis. PLoS One 2014;9(11):e113909. 25426929 10.1371/journal.pone.0113909 PMC4245236

[68] Tikunov Y, Laptenok S, Hall R MSClust: a tool for unsupervised mass spectra extraction of chromatography-mass spectrometry ion-wise aligned data. Metabolomics 2012;8(4):714–8. 22833709 10.1007/s11306-011-0368-2 PMC3397229

[69] Broeckling CD, Afsar F, Neumann S RAMClust: a novel feature clustering method enables spectral-matching-based annotation for metabolomics data. Anal Chem 2014;86(14):6812–7. 24927477 10.1021/ac501530d

[70] Gu H, Gowda GN, Neto FC RAMSY: ratio analysis of mass spectrometry to improve compound identification. Anal Chem 2013;85(22):10771–9. 24168717 10.1021/ac4019268 PMC3867450

[71] Chen G, Cui L, Teo GS MetTailor: dynamic block summary and intensity normalization for robust analysis of mass spectrometry data in metabolomics. Bioinformatics 2015;31(22):3645–52. 26220962 10.1093/bioinformatics/btv434

[72] Chawade A, Alexandersson E, Levander F Normalyzer: a tool for rapid evaluation of normalization methods for omics data sets. J Proteome Res 2014;13(6):3114–20. 24766612 10.1021/pr401264n PMC4053077

[73] Fernández-Albert F, Llorach R, Garcia-Aloy M Intensity drift removal in LC/MS metabolomics by common variance compensation. Bioinformatics 2014;30(20):2899–905. 24990606 10.1093/bioinformatics/btu423

[74] Shen X, Gong X, Cai Y Normalization and integration of large-scale metabolomics data using support vector regression. Metabolomics 2016;12(5):1–12.

[75] Karpievitch YV, Nikolic SB, Wilson R Metabolomics data normalization with EigenMS. PLoS One 2015;9(12):e116221. 10.1371/journal.pone.0116221 PMC4280143 25549083

[76] Styczynski MP, Moxley JF, Tong LV Systematic identification of conserved metabolites in GC/MS data for metabolomics and biomarker discovery. Anal Chem 2007;79(3):966–73. 17263323 10.1021/ac0614846

[77] Baran R, Kochi H, Saito N MathDAMP: a package for differential analysis of metabolite profiles. BMC Bioinformatics 2006;7(1):530. 17166258 10.1186/1471-2105-7-530 PMC1764210

[78] Huege J, Goetze J, Dethloff F Quantification of stable isotope label in metabolites via mass spectrometry. Methods Mol Biol 2014:213–23. 10.1007/978-1-62703-592-7_20 24306876

[79] Millard P, Letisse F, Sokol S IsoCor: correcting MS data in isotope labeling experiments. Bioinformatics 2012;28(9):1294–6. 22419781 10.1093/bioinformatics/bts127

[80] Jungreuthmayer C, Neubauer S, Mairinger T ICT: isotope correction toolbox. Bioinformatics 2016;32(1):154–6. 26382193 10.1093/bioinformatics/btv514

[81] Chokkathukalam A, Jankevics A, Creek DJ mzMatch–ISO: an R tool for the annotation and relative quantification of isotope-labelled mass spectrometry data. Bioinformatics 2013;29(2):281–3. 23162054 10.1093/bioinformatics/bts674 PMC3546800

[82] Bueschl C, Kluger B, Berthiller F MetExtract: a new software tool for the automated comprehensive extraction of metabolite-derived LC/MS signals in metabolomics research. Bioinformatics 2012;28(5):736–8. 22238263 10.1093/bioinformatics/bts012 PMC3289915

[83] Huang X, Chen Y-J, Cho K X13CMS: global tracking of isotopic labels in untargeted metabolomics. Anal Chem 2014;86(3):1632–9. 24397582 10.1021/ac403384n PMC3982964

[84] Capellades J, Navarro M, Samino S geoRge: a computational tool to detect the presence of stable isotope labeling in LC/MS-based untargeted metabolomics. Anal Chem 2015;88(1):621–8. 26639619 10.1021/acs.analchem.5b03628

[85] Weindl D, Wegner A, Hiller K MIA: non-targeted mass isotopolome analysis. Bioinformatics 2016;32(18):2875–6. 27273671 10.1093/bioinformatics/btw317 PMC5018370

[86] Cai Y, Weng K, Guo Y An integrated targeted metabolomic platform for high-throughput metabolite profiling and automated data processing. Metabolomics 2015;11(6):1575–86.

[87] Wong JW, Abuhusain HJ, McDonald KL MMSAT: automated quantification of metabolites in selected reaction monitoring experiments. Anal Chem 2011;84(1):470–4. 22111688 10.1021/ac2026578

[88] Tsugawa H, Arita M, Kanazawa M MRMPROBS: a data assessment and metabolite identification tool for large-scale multiple reaction monitoring based widely targeted metabolomics. Anal Chem 2013;85(10):5191–9. 23581547 10.1021/ac400515s

[89] Nikolskiy I, Mahieu NG, Chen Y-J An untargeted metabolomic workflow to improve structural characterization of metabolites. Anal Chem 2013;85(16):7713–9. 23829391 10.1021/ac400751j PMC3983953

[90] Tsugawa H, Cajka T, Kind T MS-DIAL: data-independent MS/MS deconvolution for comprehensive metabolome analysis. Nat Methods 2015;12(6):523–6. 25938372 10.1038/nmeth.3393 PMC4449330

[91] Li H, Cai Y, Guo Y MetDIA: targeted metabolite extraction of multiplexed MS/MS spectra generated by data-independent acquisition. Anal Chem 2016;88(17):8757–64. 27462997 10.1021/acs.analchem.6b02122

[92] Libiseller G, Dvorzak M, Kleb U IPO: a tool for automated optimization of XCMS parameters. BMC Bioinformatics 2015;16(1):118. 25888443 10.1186/s12859-015-0562-8 PMC4404568

[93] Mahieu NG, Huang X, Chen Y-J Credentialing features: a platform to benchmark and optimize untargeted metabolomic methods. Anal Chem 2014;86(19):9583–9. 25160088 10.1021/ac503092d PMC4188275

[94] Brodsky L, Moussaieff A, Shahaf N Evaluation of peak picking quality in LC−MS metabolomics data. Anal Chem 2010;82(22):9177–87. 20977194 10.1021/ac101216e

[95] Ranjbar MRN, Di Poto C, Wang Y SIMAT: GC-SIM-MS data analysis tool. BMC Bioinformatics 2015;16(1):259. 26283310 10.1186/s12859-015-0681-2 PMC4539696

[96] https://github.com/dgrapov/DeviumWeb (15 June 2017, date last accessed).

[97] http://biostatflow.org/ (15 June 2017, date last accessed).

[98] Mak TD, Laiakis EC, Goudarzi M Metabolyzer: a novel statistical workflow for analyzing postprocessed LC–MS metabolomics data. Anal Chem 2013;86(1):506–13. 24266674 10.1021/ac402477z PMC3973431

[99] Kastenmüller G, Römisch-Margl W, Wägele B metaP-server: a web-based metabolomics data analysis tool. BioMed Res Int 2010; DOI: 10.1155/2011/839862. PMC2946609 20936179

[100] https://fusion.cebitec.uni-bielefeld.de/Fusion/login (15 June 2017, date last accessed).

[101] Fitzpatrick MA, McGrath CM, Young SP Pathomx: an interactive workflow-based tool for the analysis of metabolomic data. BMC Bioinformatics 2014;15(1):396. 25490956 10.1186/s12859-014-0396-9 PMC4271363

[102] Hughes G, Cruickshank-Quinn C, Reisdorph R MSPrep—summarization, normalization and diagnostics for processing of mass spectrometry–based metabolomic data. Bioinformatics 2014;30(1):133–4. 24174567 10.1093/bioinformatics/btt589 PMC3866554

[103] http://mixomics.org/ (15 June 2017, date last accessed).

[104] Sun X, Weckwerth W COVAIN: a toolbox for uni-and multivariate statistics, time-series and correlation network analysis and inverse estimation of the differential Jacobian from metabolomics covariance data. Metabolomics 2012;8(1):81–93.

[105] Glaab E, Schneider R RepExplore: addressing technical replicate variance in proteomics and metabolomics data analysis. Bioinformatics 2015;31(13):2235–7. 25717197 10.1093/bioinformatics/btv127 PMC4481852

[106] Zhan X, Patterson AD, Ghosh D Kernel approaches for differential expression analysis of mass spectrometry-based metabolomics data. BMC Bioinformatics 2015;16(1):77. 25887233 10.1186/s12859-015-0506-3 PMC4359587

[107] Nodzenski M, Muehlbauer MJ, Bain JR Metabomxtr: an R package for mixture-model analysis of non-targeted metabolomics data. Bioinformatics 2014;30(22):3287–8. 25075114 10.1093/bioinformatics/btu509 PMC4221120

[108] Suvitaival T, Rogers S, Kaski S Stronger findings from mass spectral data through multi-peak modeling. BMC Bioinformatics 2014;15(1):208. 24947013 10.1186/1471-2105-15-208 PMC4080774

[109] Mak TD, Laiakis EC, Goudarzi M Selective paired ion contrast analysis: a novel algorithm for analyzing postprocessed LC-MS metabolomics data possessing high experimental noise. Anal Chem 2015;87(6):3177–86. 25683158 10.1021/ac504012a PMC4519008

[110] Ernest B, Gooding JR, Campagna SR MetabR: an R script for linear model analysis of quantitative metabolomic data. BMC Res Notes 2012;5(1):596. 23111096 10.1186/1756-0500-5-596 PMC3532230

[111] Huang J-H, Yan J, Wu Q-H Selective of informative metabolites using random forests based on model population analysis. Talanta 2013;117:549–55. 24209380 10.1016/j.talanta.2013.07.070

[112] Simader AM, Kluger B, Neumann NKN QCScreen: a software tool for data quality control in LC-HRMS based metabolomics. BMC Bioinformatics 2015;16(1):341. 26498454 10.1186/s12859-015-0783-x PMC4619325

[113] Fernie AR The future of metabolic phytochemistry: larger numbers of metabolites, higher resolution, greater understanding. Phytochemistry 2007;68(22–24):2861–80. 17804028 10.1016/j.phytochem.2007.07.010

[114] Tohge T, Wendenburg R, Ishihara H Characterization of a recently evolved flavonol-phenylacyltransferase gene provides signatures of natural light selection in Brassicaceae. Nat Commun 2016;7. 10.1038/ncomms12399 PMC4996938 27545969

[115] Schymanski E, Neumann S CASMI: and the winner is. Metabolites 2013;3(2):412. 24957999 10.3390/metabo3020412 PMC3901266

[116] Schymanski EL, Ruttkies C, Krauss M Critical assessment of small molecule identification 2016: automated methods. J Cheminformatics 2017;9(1):22. 10.1186/s13321-017-0207-1 PMC5368104 29086042

[117] Zhou B, Wang J, Ressom HW MetaboSearch: tool for mass-based metabolite identification using multiple databases. PLoS One 2012;7(6):e40096. 22768229 10.1371/journal.pone.0040096 PMC3387018

[118] Brown M, Wedge DC, Goodacre R Automated workflows for accurate mass-based putative metabolite identification in LC/MS-derived metabolomic datasets. Bioinformatics 2011;27(8):1108–12. 21325300 10.1093/bioinformatics/btr079 PMC3709197

[119] Daly R, Rogers S, Wandy J MetAssign: probabilistic annotation of metabolites from LC–MS data using a Bayesian clustering approach. Bioinformatics 2014;30(19):2764–71. 24916385 10.1093/bioinformatics/btu370 PMC4173012

[120] Böcker S, Letzel MC, Lipták Z SIRIUS: decomposing isotope patterns for metabolite identification. Bioinformatics 2009;25(2):218–24. 19015140 10.1093/bioinformatics/btn603 PMC2639009

[121] Sakurai N, Ara T, Kanaya S An application of a relational database system for high-throughput prediction of elemental compositions from accurate mass values. Bioinformatics 2013;29(2):290–1. 23162084 10.1093/bioinformatics/bts660

[122] Lommen A Ultrafast PubChem searching combined with improved filtering rules for elemental composition analysis. Anal Chem 2014;86(11):5463–9. 24820703 10.1021/ac500667h

[123] Tsugawa H, Kind T, Nakabayashi R Hydrogen rearrangement rules: computational MS/MS fragmentation and structure elucidation using MS-FINDER software. Anal Chem 2016;88(16):7946–58. 27419259 10.1021/acs.analchem.6b00770 PMC7063832

[124] Ma Y, Kind T, Yang D MS2Analyzer: a software for small molecule substructure annotations from accurate tandem mass spectra. Anal Chem 2014;86(21):10724–31. 25263576 10.1021/ac502818e PMC4222628

[125] van der Hooft JJJ, Wandy J, Barrett MP Topic modeling for untargeted substructure exploration in metabolomics. Proc Natl Acad Sci U S A 2016;113(48):13738–43. 27856765 10.1073/pnas.1608041113 PMC5137707

[126] Dhanasekaran AR, Pearson JL, Ganesan B Metabolome searcher: a high throughput tool for metabolite identification and metabolic pathway mapping directly from mass spectrometry and using genome restriction. BMC Bioinformatics 2015;16(1):62. 25887958 10.1186/s12859-015-0462-y PMC4347650

[127] Suhre K, Schmitt-Kopplin P MassTRIX: mass translator into pathways. Nucleic Acids Res 2008;36(suppl 2):W481–4. 18442993 10.1093/nar/gkn194 PMC2447776

[128] Uppal K, Soltow QA, Promislow DE MetabNet: an R package for metabolic association analysis of high-resolution metabolomics data. Front Bioeng Biotechnol 2015;3:87. 26125020 10.3389/fbioe.2015.00087 PMC4464066

[129] Silva RR, Jourdan F, Salvanha DM ProbMetab: an R package for Bayesian probabilistic annotation of LC–MS-based metabolomics. Bioinformatics 2014;30(9):1336–7. 24443383 10.1093/bioinformatics/btu019 PMC3998140

[130] Rogers S, Scheltema RA, Girolami M Probabilistic assignment of formulas to mass peaks in metabolomics experiments. Bioinformatics 2009;25(4):512–8. 19095699 10.1093/bioinformatics/btn642

[131] Weber RJ, Viant MR MI-Pack: increased confidence of metabolite identification in mass spectra by integrating accurate masses and metabolic pathways. Chemom Intell Lab Syst 2010;104(1):75–82.

[132] Qiu F, Fine DD, Wherritt DJ PlantMAT: a metabolomics tool for predicting the specialized metabolic potential of a system and for large-scale metabolite identifications. Anal Chem 2016;88(23):11373–83. 27934098 10.1021/acs.analchem.6b00906

[133] Ruttkies C, Schymanski EL, Wolf S MetFrag relaunched: incorporating strategies beyond in silico fragmentation. J Cheminformatics 2016;8(1):3. 10.1186/s13321-016-0115-9 PMC4732001 26834843

[134] Menikarachchi LC, Cawley S, Hill DW MolFind: a software package enabling HPLC/MS-based identification of unknown chemical structures. Anal Chem 2012;84(21):9388–94. 23039714 10.1021/ac302048x PMC3523192

[135] Allen F, Pon A, Wilson M CFM-ID: a web server for annotation, spectrum prediction and metabolite identification from tandem mass spectra. Nucleic Acids Res 2014;42(W1):W94–9. 24895432 10.1093/nar/gku436 PMC4086103

[136] Ridder L, van der Hooft JJ, Verhoeven S Automatic compound annotation from mass spectrometry data using MAGMa. Mass Spectrometry 2014;3(Special_Issue_2):S0033. 26819876 10.5702/massspectrometry.S0033 PMC4321337

[137] Heinonen M, Shen H, Zamboni N Metabolite identification and molecular fingerprint prediction through machine learning. Bioinformatics 2012;28(18):2333–41. 22815355 10.1093/bioinformatics/bts437

[138] Dührkop K, Shen H, Meusel M Searching molecular structure databases with tandem mass spectra using CSI: FingerID. Proc Natl Acad Sci U S A 2015;112(41):12580–5. 26392543 10.1073/pnas.1509788112 PMC4611636

[139] Brouard C, Shen H, Dührkop K Fast metabolite identification with input output kernel regression. Bioinformatics 2016;32(12):i28–36. 27307628 10.1093/bioinformatics/btw246 PMC4908330

[140] Gerlich M, Neumann S MetFusion: integration of compound identification strategies. J Mass Spectrom 2013;48(3):291–8. 23494783 10.1002/jms.3123

[141] Leader DP, Burgess K, Creek D Pathos: a web facility that uses metabolic maps to display experimental changes in metabolites identified by mass spectrometry. Rapid Commun Mass Spectrom 2011;25(22):3422–6. 22002696 10.1002/rcm.5245 PMC3509215

[142] Pon A, Jewison T, Su Y Pathways with PathWhiz. Nucleic Acids Res 2015;43(W1):W552–9. 25934797 10.1093/nar/gkv399 PMC4489271

[143] Yamada T, Letunic I, Okuda S iPath2. 0: Interactive Pathway Explorer. Nucleic Acids Res 2011;39(suppl 2):W412–5. 21546551 10.1093/nar/gkr313 PMC3125749

[144] Kutmon M, van Iersel MP, Bohler A PathVisio 3: an extendable pathway analysis toolbox. PLoS Comput Biol 2015;11(2):e1004085. 25706687 10.1371/journal.pcbi.1004085 PMC4338111

[145] Pathan M, Keerthikumar S, Ang CS FunRich: an open access standalone functional enrichment and interaction network analysis tool. Proteomics 2015;15(15):2597–601. 25921073 10.1002/pmic.201400515

[146] Moreno P, Beisken S, Harsha B BiNChE: a web tool and library for chemical enrichment analysis based on the ChEBI ontology. BMC Bioinformatics 2015;16(1):56. 25879798 10.1186/s12859-015-0486-3 PMC4349482

[147] Kankainen M, Gopalacharyulu P, Holm L MPEA—metabolite pathway enrichment analysis. Bioinformatics 2011;27(13):1878–9. 21551139 10.1093/bioinformatics/btr278

[148] Aggio RB, Ruggiero K, Villas-Bôas SG Pathway activity profiling (PAPi): from the metabolite profile to the metabolic pathway activity. Bioinformatics 2010;26(23):2969–76. 20929912 10.1093/bioinformatics/btq567

[149] Eichner J, Rosenbaum L, Wrzodek C Integrated enrichment analysis and pathway-centered visualization of metabolomics, proteomics, transcriptomics, and genomics data by using the InCroMAP software. J Chromatogr B 2014;966:77–82. 10.1016/j.jchromb.2014.04.030 24811976

[150] Carazzolle MF, de Carvalho LM, Slepicka HH IIS–Integrated Interactome System: a web-based platform for the annotation, analysis and visualization of protein-metabolite-gene-drug interactions by integrating a variety of data sources and tools. PLoS One 2014;9(6):e100385. 24949626 10.1371/journal.pone.0100385 PMC4065059

[151] Sakurai N, Ara T, Ogata Y KaPPA-View4: a metabolic pathway database for representation and analysis of correlation networks of gene co-expression and metabolite co-accumulation and omics data. Nucleic Acids Res 2011;39(suppl 1):D677–84. 21097783 10.1093/nar/gkq989 PMC3013809

[152] Usadel B, Poree F, Nagel A A guide to using MapMan to visualize and compare omics data in plants: a case study in the crop species, Maize. Plant Cell Environ 2009;32(9):1211–29. 19389052 10.1111/j.1365-3040.2009.01978.x

[153] Neuweger H, Persicke M, Albaum SP Visualizing post genomics data-sets on customized pathway maps by ProMeTra–aeration-dependent gene expression and metabolism of Corynebacterium glutamicum as an example. BMC Syst Biol 2009;3(1):82. 19698148 10.1186/1752-0509-3-82 PMC2744654

[154] García-Alcalde F, García-López F, Dopazo J Paintomics: a web based tool for the joint visualization of transcriptomics and metabolomics data. Bioinformatics 2011;27(1):137–9. 21098431 10.1093/bioinformatics/btq594 PMC3008637

[155] Rohn H, Junker A, Hartmann A VANTED v2: a framework for systems biology applications. BMC Syst Biol 2012;6(1):139. 23140568 10.1186/1752-0509-6-139 PMC3610154

[156] López-Ibáñez J, Pazos F, Chagoyen M MBROLE 2.0—functional enrichment of chemical compounds. Nucleic Acids Res 2016;44(W1):W201–4. 27084944 10.1093/nar/gkw253 PMC4987872

[157] Kamburov A, Cavill R, Ebbels TM Integrated pathway-level analysis of transcriptomics and metabolomics data with IMPaLA. Bioinformatics 2011;27(20):2917–8. 21893519 10.1093/bioinformatics/btr499

[158] Jourdan F, Breitling R, Barrett MP MetaNetter: inference and visualization of high-resolution metabolomic networks. Bioinformatics 2008;24(1):143–5. 18003642 10.1093/bioinformatics/btm536

[159] Grapov D, Wanichthanarak K, Fiehn O MetaMapR: pathway independent metabolomic network analysis incorporating unknowns. Bioinformatics 2016;31(16):2757–60. 10.1093/bioinformatics/btv194 PMC4528626 25847005

[160] Lu J, Carlson HA ChemTreeMap: an interactive map of biochemical similarity in molecular datasets. Bioinformatics 2016;32(23):3584–92. 27515740 10.1093/bioinformatics/btw523 PMC5181537

[161] Treutler H, Tsugawa H, Porzel A Discovering regulated metabolite families in untargeted metabolomics studies. Anal Chem 2016;88(16):8082–90. 27452369 10.1021/acs.analchem.6b01569

[162] Naake T, Gaquerel E MetCirc: navigating mass spectral similarity in high-resolution MS/MS metabolomics data. Bioinformatics 2017. 10.1093/bioinformatics/btx159 28402393

[163] Hamdalla MA, Rajasekaran S, Grant DF Metabolic pathway predictions for metabolomics: a molecular structure matching approach. J Chem Inf Model 2015;55(3):709–18. 25668446 10.1021/ci500517v PMC8386145

[164] Pence HE, Williams A ChemSpider: an online chemical information resource. J Chem Educ 2010; DOI: 10.1021/ed100697w.

[165] Kim S, Thiessen PA, Bolton EE PubChem substance and compound databases. Nucleic Acids Res 2015; DOI: 10.1093/nar/gkv951. PMC4702940 26400175

[166] Hastings J, Owen G, Dekker A ChEBI in 2016: improved services and an expanding collection of metabolites. Nucleic Acids Res 2015; DOI: 10.1093/nar/gkv1031. PMC4702775 26467479

[167] Gaulton A, Bellis LJ, Bento AP ChEMBL: a large-scale bioactivity database for drug discovery. Nucleic Acids Res 2012;40(D1):D1100–7. 21948594 10.1093/nar/gkr777 PMC3245175

[168] Seiler KP, George GA, Happ MP ChemBank: a small-molecule screening and cheminformatics resource database. Nucleic Acids Res 2008;36(suppl 1):D351–9. 17947324 10.1093/nar/gkm843 PMC2238881

[169] Wishart DS, Jewison T, Guo AC HMDB 3.0—the human metabolome database in 2013. Nucleic Acids Res 2012; DOI: 10.1093/nar/gks1065. PMC3531200 23161693

[170] Cui Q, Lewis IA, Hegeman AD Metabolite identification via the madison metabolomics consortium database. Nat Biotech 2008;26(2):162–4. 10.1038/nbt0208-162 18259166

[171] Masciocchi J, Frau G, Fanton M MMsINC: a large-scale chemoinformatics database. Nucleic Acids Res 2009;37(suppl 1):D284–90. 18931373 10.1093/nar/gkn727 PMC2686567

[172] Afendi FM, Okada T, Yamazaki M KNApSAcK family databases: integrated metabolite–plant species databases for multifaceted plant research. Plant Cell Physiol 2012;53(2):e1. 22123792 10.1093/pcp/pcr165

[173] Gu J, Gui Y, Chen L Use of natural products as chemical library for drug discovery and network pharmacology. PLoS One 2013;8(4):e62839. 23638153 10.1371/journal.pone.0062839 PMC3636197

[174] Arita M, Suwa K Search extension transforms Wiki into a relational system: a case for flavonoid metabolite database. BioData Mining 2008;1(1):7. 18822113 10.1186/1756-0381-1-7 PMC2556319

[175] https://phytochem.nal.usda.gov/phytochem/search (15 June 2017, date last accessed).

[176] Sharma A, Dutta P, Sharma M BioPhytMol: a drug discovery community resource on anti-mycobacterial phytomolecules and plant extracts. J Cheminformatics 2014;6(1):46. 10.1186/s13321-014-0046-2 PMC4206768 25360160

[177] Kumari S, Pundhir S, Priya P EssOilDB: a database of essential oils reflecting terpene composition and variability in the plant kingdom. Database (Oxford) 2014;2014: DOI: 10.1093/database/bau120. PMC4273207 25534749

[178] http://webbook.nist.gov/chemistry/ (15 June 2017, date last accessed).

[179] http://sdbs.db.aist.go.jp/sdbs/cgi-bin/cre_index.cgi (15 June 2017, date last accessed).

[180] Hummel J, Selbig J, Walther D The golm metabolome database: a database for GC-MS based metabolite profiling. Metabolomics 2007;18:75–95.

[181] Skogerson K, Wohlgemuth G, Barupal DK The volatile compound BinBase mass spectral database. BMC Bioinformatics 2011;12(1):321. 21816034 10.1186/1471-2105-12-321 PMC3199763

[182] Tobias K, Wohlgemuth G, Lee DY FiehnLib: mass spectral and retention index libraries for metabolomics based on quadrupole and time-of-flight gas chromatography/mass spectrometry. Anal Chem 2009;81(24):10038–48. 19928838 10.1021/ac9019522 PMC2805091

[183] Horai H, Arita M, Kanaya S MassBank: a public repository for sharing mass spectral data for life sciences. J Mass Spectrom 2010;45(7):703–14. 20623627 10.1002/jms.1777

[184] Smith CA, O’Maille G, Want EJ METLIN: a metabolite mass spectral database. Ther Drug Monit 2005;27(6):747–51. 16404815 10.1097/01.ftd.0000179845.53213.39

[185] Cho K, Mahieu N, Ivanisevic J isoMETLIN: a database for isotope-based metabolomics. Anal Chem 2014;86(19):9358–61. 25166490 10.1021/ac5029177 PMC5729911

[186] Wishart D, Arndt D, Pon A T3DB: the toxic exposome database. Nucleic Acids Res 2015;43(D1):D928–34. 25378312 10.1093/nar/gku1004 PMC4383875

[187] https://www.mzcloud.org/ (15 June 2017, date last accessed).

[188] http://mona.fiehnlab.ucdavis.edu/ (15 June 2017, date last accessed).

[189] Cuthbertson DJ, Johnson SR, Piljac-Žegarac J Accurate mass–time tag library for LC/MS-based metabolite profiling of medicinal plants. Phytochemistry 2013;91:187–97. 23597491 10.1016/j.phytochem.2013.02.018 PMC3697863

[190] http://webs2.kazusa.or.jp/msmsfragmentviewer/ (15 June 2017, date last accessed).

[191] Sawada Y, Nakabayashi R, Yamada Y RIKEN tandem mass spectral database (ReSpect) for phytochemicals: a plant-specific MS/MS-based data resource and database. Phytochemistry 2012;82:38–45. 22867903 10.1016/j.phytochem.2012.07.007

[192] Shahaf N, Rogachev I, Heinig U The WEIZMASS spectral library for high-confidence metabolite identification. Nat Commun 2016;7. 10.1038/ncomms12423 PMC5013563 27571918

[193] Weber RJM, Li E, Bruty J MaConDa: a publicly accessible mass spectrometry contaminants database. Bioinformatics 2012;28(21):2856–7. 22954629 10.1093/bioinformatics/bts527

[194] Matsuda F, Hirai MY, Sasaki E AtMetExpress development: a phytochemical atlas of Arabidopsis development. Plant Physiol 2010;152(2):566–78. 20023150 10.1104/pp.109.148031 PMC2815869

[195] Fukushima A, Kusano M, Mejia RF Metabolomic characterization of knockout mutants in Arabidopsis: development of a metabolite profiling database for knockout mutants in Arabidopsis. Plant Physiol 2014;165(3):948–61. 24828308 10.1104/pp.114.240986 PMC4081348

[196] Moco S, Bino RJ, Vorst O A liquid chromatography-mass spectrometry-based metabolome database for tomato. Plant Physiol 2006;141(4):1205–18. 16896233 10.1104/pp.106.078428 PMC1533921

[197] Brockmöller T, Ling Z, Li D Nicotiana attenuata Data Hub (Na DH): an integrative platform for exploring genomic, transcriptomic and metabolomic data in wild tobacco. BMC Genomics 2017;18(1):79. 28086860 10.1186/s12864-016-3465-9 PMC5237228

[198] Colmsee C, Mascher M, Czauderna T OPTIMAS-DW: a comprehensive transcriptomics, metabolomics, ionomics, proteomics and phenomics data resource for maize. BMC Plant Biol 2012;12(1):245. 23272737 10.1186/1471-2229-12-245 PMC3577462

[199] Joshi T, Yao Q, Levi DF SoyMetDB: the soybean metabolome database. In: International Conference on Bioinformatics and Biomedicine, BIBM 2010. pp. 203–8. Hong Kong, China: IEEE; 2010; DOI: 10.1109/BIBM.2010.5706563.

[200] Iijima Y, Nakamura Y, Ogata Y Metabolite annotations based on the integration of mass spectral information. Plant J 2008;54(5):949–62. 18266924 10.1111/j.1365-313X.2008.03434.x PMC2440531

[201] Matsuda F, Yonekura-Sakakibara K, Niida R MS/MS spectral tag-based annotation of non-targeted profile of plant secondary metabolites. Plant J 2009;57(3):555–77. 18939963 10.1111/j.1365-313X.2008.03705.x PMC2667644

[202] Hur M, Campbell AA, Almeida-de-Macedo M A global approach to analysis and interpretation of metabolic data for plant natural product discovery. Nat Prod Rep 2013;30(4):565–83. 23447050 10.1039/c3np20111b PMC3629923

[203] Wurtele ES, Chappell J, Jones AD Medicinal plants: a public resource for metabolomics and hypothesis development. Metabolites 2012;2(4):1031–59. 24957774 10.3390/metabo2041031 PMC3901233

[204] http://bio.massbank.jp/ (15 June 2017, date last accessed).

[205] http://webs2.kazusa.or.jp/massbase/ (15 June 2017, date last accessed).

[206] Sud M, Fahy E, Cotter D Metabolomics Workbench: an international repository for metabolomics data and metadata, metabolite standards, protocols, tutorials and training, and analysis tools. Nucleic Acids Res 2015; DOI: 10.1093/nar/gkv1042. PMC4702780 26467476

[207] Haug K, Salek RM, Conesa P MetaboLights—an open-access general-purpose repository for metabolomics studies and associated meta-data. Nucleic Acids Res 2012; DOI: 10.1093/nar/gks1004. PMC3531110 23109552

[208] Cook CE, Bergman MT, Finn RD The European Bioinformatics Institute in 2016: data growth and integration. Nucleic Acids Res 2016;44(D1):D20–6. 26673705 10.1093/nar/gkv1352 PMC4702932

[209] Wang M, Carver JJ, Phelan VV Sharing and community curation of mass spectrometry data with Global Natural Products Social Molecular Networking. Nat Biotechnol 2016;34(8):828–37. 27504778 10.1038/nbt.3597 PMC5321674

[210] Kanehisa M, Goto S KEGG: Kyoto Encyclopedia of Genes and Genomes. Nucleic Acids Res. 2000;28(1):27–30. 10592173 10.1093/nar/28.1.27 PMC102409

[211] Kelder T, Pico AR, Hanspers K Mining biological pathways using WikiPathways web services. PLoS One 2009;4(7):e6447. 19649250 10.1371/journal.pone.0006447 PMC2714472

[212] Navas-Delgado I, García-Godoy MJ, López-Camacho E kpath: integration of metabolic pathway linked data. Database (Oxford) 2015 https://doi.org/10.1093/database/bav053 (15 June 2017, date last accessed). 10.1093/database/bav053 PMC4460419 26055101

[213] Caspi R, Foerster H, Fulcher CA The MetaCyc Database of metabolic pathways and enzymes and the BioCyc collection of pathway/genome databases. Nucleic Acids Res 2008;36 (suppl 1):D623–31. 17965431 10.1093/nar/gkm900 PMC2238876

[214] Arkin AP, Stevens RL, Cottingham RW The DOE systems biology knowledgebase (KBase). bioRxiv 2016:096354.

[215] http://www.plantcyc.org/ (15 June 2017, date last accessed).

[216] Schreiber F, Colmsee C, Czauderna T MetaCrop 2.0: managing and exploring information about crop plant metabolism. Nucleic Acids Res 2011; DOI: 10.1093/nar/gkr1004. PMC3245004 22086948

[217] Sucaet Y, Wang Y, Li J MetNet online: a novel integrated resource for plant systems biology. BMC Bioinformatics 2012;13(1):267. 23066841 10.1186/1471-2105-13-267 PMC3483157

[218] Tello-Ruiz MK, Stein J, Wei S Gramene 2016: comparative plant genomics and pathway resources. Nucleic Acids Res 2015; DOI: 10.1093/nar/gkv1179. PMC4702844 26553803

[219] Ott MA, Vriend G Correcting ligands, metabolites, and pathways. BMC Bioinformatics 2006;7(1):517. 17132165 10.1186/1471-2105-7-517 PMC1686944

[220] Lang M, Stelzer M, Schomburg D BKM-react, an integrated biochemical reaction database. BMC Biochem 2011;12(1):42. 21824409 10.1186/1471-2091-12-42 PMC3167764

[221] Kumar A, Suthers PF, Maranas CD MetRxn: a knowledgebase of metabolites and reactions spanning metabolic models and databases. BMC Bioinformatics 2012;13(1):6. 22233419 10.1186/1471-2105-13-6 PMC3277463

[222] Jeffryes JG, Colastani RL, Elbadawi-Sidhu M MINEs: open access databases of computationally predicted enzyme promiscuity products for untargeted metabolomics. J Cheminformatics. 2015;7(1):44. 10.1186/s13321-015-0087-1 PMC4550642 26322134

[223] Zhou Z, Shen X, Tu J Large-scale prediction of collision cross-section values for metabolites in ion mobility-mass spectrometry. Anal Chem 2016;88(22):11084–91. 27768289 10.1021/acs.analchem.6b03091

[224] Allard P-M, Péresse T, Bisson J Integration of molecular networking and in-silico MS/MS fragmentation for natural products dereplication. Anal Chem 2016;88(6):3317–23. 26882108 10.1021/acs.analchem.5b04804

[225] Palla P, Frau G, Vargiu L QTREDS: a Ruby on Rails-based platform for omics laboratories. BMC Bioinformatics 2014;15(1):S13. 10.1186/1471-2105-15-S1-S13 PMC4015218 24564791

[226] Hunter A, Dayalan S, De Souza D MASTR-MS: a web-based collaborative laboratory information management system (LIMS) for metabolomics. Metabolomics 2017;13(2):14. 28090199 10.1007/s11306-016-1142-2 PMC5192047

[227] Franceschi P, Mylonas R, Shahaf N MetaDB a data processing workflow in untargeted MS-based metabolomics experiments. Front Bioen Biotechnol 2014;(2):72. 10.3389/fbioe.2014.00072 PMC4267269 25566535

[228] Ara T, Enomoto M, Arita M Metabolonote: a wiki-based database for managing hierarchical metadata of metabolome analyses. Front Bioeng Biotechnol 2015;(3):38. 10.3389/fbioe.2015.00038 PMC4388006 25905099

[229] Wohlgemuth G, Mehta SS, Mejia RF SPLASH, a hashed identifier for mass spectra. Nat Biotechnol 2016;34(11):1099–101. 27824832 10.1038/nbt.3689 PMC5515539

[230] Redestig H, Kusano M, Fukushima A Consolidating metabolite identifiers to enable contextual and multi-platform metabolomics data analysis. BMC Bioinformatics 2010;11(1):214. 20426876 10.1186/1471-2105-11-214 PMC2879285

[231] Wohlgemuth G, Haldiya PK, Willighagen E The Chemical Translation Service—a web-based tool to improve standardization of metabolomic reports. Bioinformatics 2010;26(20):2647–8. 20829444 10.1093/bioinformatics/btq476 PMC2951090

[232] Carroll AJ, Zhang P, Whitehead L PhenoMeter: a metabolome database search tool using statistical similarity matching of metabolic phenotypes for high-confidence detection of functional links. Front Bioeng Biotechnol 2015;3. 10.3389/fbioe.2015.00106 PMC4518198 26284240

[233] Sartor MA, Ade A, Wright Z Metab2MeSH: annotating compounds with medical subject headings. Bioinformatics 2012;28(10):1408–10. 22492643 10.1093/bioinformatics/bts156 PMC3348562

[234] Chambers MC, MacLean B, Burke R A cross-platform toolkit for mass spectrometry and proteomics. Nat Biotechnol 2012;30:918–20. 23051804 10.1038/nbt.2377 PMC3471674

[235] Xu QW, Griss J, Wang R jmzTab: a Java interface to the mzTab data standard. Proteomics 2014;14(11):1328–32. 24659499 10.1002/pmic.201300560 PMC4230411

[236] Scheltema RA, Jankevics A, Jansen RC PeakML/mzMatch: a file format, Java library, R library, and tool-chain for mass spectrometry data analysis. Anal Chem 2011;83(7):2786–93. 21401061 10.1021/ac2000994

[237] Avtonomov DM, Raskind A, Nesvizhskii AI BatMass: a Java software platform for LC–MS data visualization in proteomics and metabolomics. J Proteome Res 2016;15(8):2500–9. 27306858 10.1021/acs.jproteome.6b00021 PMC5583644

[238] Tanaka S, Fujita Y, Parry HE Mass^++^: a visualization and analysis tool for mass spectrometry. J Proteome Res 2014;13(8):3846–53. 10.1021/pr500155z 24965016

[239] Beisken S, Conesa P, Haug K SpeckTackle: JavaScript charts for spectroscopy. J Cheminformatics 2015;7(1):17. 10.1186/s13321-015-0065-7 PMC4432097 25984241

[240] Stravs MA, Schymanski EL, Singer HP Automatic recalibration and processing of tandem mass spectra using formula annotation. J Mass Spectrom 2013;48(1):89–99. 23303751 10.1002/jms.3131

[241] Dong Y, Li B, Aharoni A More than pictures: when MS imaging meets histology. Trends Plant Sci 2016;21(8):686–98. 27155743 10.1016/j.tplants.2016.04.007

[242] Wijetunge CD, Saeed I, Boughton BA EXIMS: an improved data analysis pipeline based on a new peak picking method for EXploring imaging mass spectrometry data. Bioinformatics 2015;31(19):3198–206. 26063840 10.1093/bioinformatics/btv356

[243] Rübel O, Greiner A, Cholia S OpenMSI: a high-performance web-based platform for mass spectrometry imaging. Anal Chem 2013;85(21):10354–61. 24087878 10.1021/ac402540a

[244] Husen P, Tarasov K, Katafiasz M Analysis of lipid experiments (ALEX): a software framework for analysis of high-resolution shotgun lipidomics data. PLoS One 2013;8(11):e79736. 24244551 10.1371/journal.pone.0079736 PMC3820610

[245] Tsugawa H, Ohta E, Izumi Y MRM-DIFF: data processing strategy for differential analysis in large scale MRM-based lipidomics studies. Front Genet 2014;5. 10.3389/fgene.2014.00471 PMC4311682 25688256

[246] Wong G, Chan J, Kingwell BA LICRE: unsupervised feature correlation reduction for lipidomics. Bioinformatics 2014; DOI: 10.1093/bioinformatics/btu381. PMC4173018 24930143

[247] Herzog R, Schuhmann K, Schwudke D LipidXplorer: a software for consensual cross-platform lipidomics. PLoS One 2012;7(1):e29851. 22272252 10.1371/journal.pone.0029851 PMC3260173

[248] Haimi P, Uphoff A, Hermansson M Software tools for analysis of mass spectrometric lipidome data. Anal Chem 2006;78(24):8324–31. 17165823 10.1021/ac061390w

[249] Blanchard AP, McDowell GS, Valenzuela N Visualization and Phospholipid Identification (VaLID): online integrated search engine capable of identifying and visualizing glycerophospholipids with given mass. Bioinformatics 2013;29(2):284–5. 23162086 10.1093/bioinformatics/bts662 PMC3546797

[250] Collins JR, Edwards BR, Fredricks HF LOBSTAHS: an adduct-based lipidomics strategy for discovery and identification of oxidative stress biomarkers. Anal Chem 2016;88(14):7154–62. 27322848 10.1021/acs.analchem.6b01260

[251] Ahmed Z, Mayr M, Zeeshan S Lipid-Pro: a computational lipid identification solution for untargeted lipidomics on data-independent acquisition tandem mass spectrometry platforms. Bioinformatics 2015;31(7):1150–3. 25433698 10.1093/bioinformatics/btu796

[252] Hartler J, Trötzmüller M, Chitraju C Lipid Data Analyzer: unattended identification and quantitation of lipids in LC-MS data. Bioinformatics 2011;27(4):572–7. 21169379 10.1093/bioinformatics/btq699

[253] Song H, Hsu F-F, Ladenson J Algorithm for processing raw mass spectrometric data to identify and quantitate complex lipid molecular species in mixtures by data-dependent scanning and fragment ion database searching. J Am Soc Mass Spectrom 2007;18(10):1848–58. 17720531 10.1016/j.jasms.2007.07.023 PMC2044497

[254] Sud M, Fahy E, Cotter D LMSD: Lipid Maps Structure Database. Nucleic Acids Res 2007;35(suppl 1):D527–32. 17098933 10.1093/nar/gkl838 PMC1669719

[255] Watanabe K, Yasugi E, Oshima M How to search the glycolipid data in “LIPIDBANK for Web”, the newly developed lipid database in Japan. Trends Glycosci Glycotechnol 2000;12(65):175–84.

[256] Kind T, Liu K-H, Lee DY LipidBlast in silico tandem mass spectrometry database for lipid identification. Nat Methods 2013;10(8):755–8. 23817071 10.1038/nmeth.2551 PMC3731409

[257] Foster JM, Moreno P, Fabregat A LipidHome: a database of theoretical lipids optimized for high throughput mass spectrometry lipidomics. PLoS One 2013;8(5):e61951. 23667450 10.1371/journal.pone.0061951 PMC3646891

[258] Aimo L, Liechti R, Nouspikel N The SwissLipids knowledgebase for lipid biology. Bioinformatics 2015; DOI: 10.1093/bioinformatics/btv285. PMC4547616 25943471

[259] Li-Beisson Y, Shorrosh B, Beisson F Acyl-Lipid Metabolism in The Arabidopsis Book, Rockville, MD: American Society of Plant Biologists. 2013;11:e0161, https://doi.org/10.1199/tab.0161 (29 June 2017, date last accessed). 10.1199/tab.0161 PMC3563272 23505340

[260] Tautenhahn R, Patti GJ, Rinehart D XCMS Online: a web-based platform to process untargeted metabolomic data. Anal Chem 2012;84(11):5035–9. 22533540 10.1021/ac300698c PMC3703953

[261] Grace SC, Embry S, Luo H Haystack, a web-based tool for metabolomics research. BMC Bioinformatics 2014;15(11):S12. 10.1186/1471-2105-15-S11-S12 PMC4251040 25350247

[262] Liang Y-J, Lin Y-T, Chen C-W SMART: statistical metabolomics analysis an R tool. Anal Chem 2016;88(12):6334–41. 27248514 10.1021/acs.analchem.6b00603

[263] Pluskal T, Castillo S, Villar-Briones A MZmine 2: modular framework for processing, visualizing, and analyzing mass spectrometry-based molecular profile data. BMC Bioinformatics 2010;11(1):395. 20650010 10.1186/1471-2105-11-395 PMC2918584

[264] Wei X, Sun W, Shi X MetSign: a computational platform for high-resolution mass spectrometry-based metabolomics. Anal Chem 2011;83(20):7668–75. 21932828 10.1021/ac2017025 PMC3196362

[265] LaMarche BL, Crowell KL, Jaitly N MultiAlign: a multiple LC-MS analysis tool for targeted omics analysis. BMC Bioinformatics 2013;14(1):49. 23398735 10.1186/1471-2105-14-49 PMC3599190

[266] Carroll AJ, Badger MR, Millar AH The MetabolomeExpress Project: enabling web-based processing, analysis and transparent dissemination of GC/MS metabolomics datasets. BMC Bioinformatics 2010;11(1):376. 20626915 10.1186/1471-2105-11-376 PMC2912306

[267] Fernández-Albert F, Llorach R, Andrés-Lacueva C An R package to analyse LC/MS metabolomic data: MAIT (Metabolite Automatic Identification Toolkit). Bioinformatics 2014;30(13):1937–9. 24642061 10.1093/bioinformatics/btu136 PMC4071204

[268] Melamud E, Vastag L, Rabinowitz JD Metabolomic analysis and visualization engine for LC−MS data. Anal Chem 2010;82(23):9818–26. 21049934 10.1021/ac1021166 PMC5748896

[269] Neuweger H, Albaum SP, Dondrup M MeltDB: a software platform for the analysis and integration of metabolomics experiment data. Bioinformatics 2008;24(23):2726–32. 18765459 10.1093/bioinformatics/btn452

[270] Xia J, Sinelnikov IV, Han B MetaboAnalyst 3.0—making metabolomics more meaningful. Nucleic Acids Res 2015;43(W1):W2517. 25897128 10.1093/nar/gkv380 PMC4489235

[271] Kaever A, Landesfeind M, Feussner K MarVis-Pathway: integrative and exploratory pathway analysis of non-targeted metabolomics data. Metabolomics 2015;11(3):764–77. 25972773 10.1007/s11306-014-0734-y PMC4419191

[272] Edmands WM, Barupal DK, Scalbert A MetMSLine: an automated and fully integrated pipeline for rapid processing of high-resolution LC-MS metabolomic datasets. Bioinformatics 2014; DOI: 10.1093/bioinformatics/btu705. PMC4341062 25348215

[273] Beisken S, Earll M, Portwood D MassCascade: visual programming for LC-MS data processing in metabolomics. Mol Inf 2014;33(4):307–10. 10.1002/minf.201400016 PMC4524413 26279687

[274] Winkler R MASSyPup—an ‘Out of the Box'solution for the analysis of mass spectrometry data. J Mass Spectrom 2014;49(1):37–42. 24446261 10.1002/jms.3314

[275] Sakurai N, Ara T, Enomoto M Tools and databases of the KOMICS web portal for preprocessing, mining, and dissemination of metabolomics data. BioMed Res Int 2014; DOI: 10.1155/2014/194812. PMC4052814 24949426

[276] http://metaopen.sourceforge.net/ (15 June 2017, date last accessed).

[277] Sakurai T, Yamada Y, Sawada Y PRIMe update: innovative content for plant metabolomics and integration of gene expression and metabolite accumulation. Plant Cell Physiol 2013;54(2):e5. 23292601 10.1093/pcp/pcs184 PMC3583026

[278] Henry VJ, Bandrowski AE, Pepin A-S OMICtools: an informative directory for multi-omic data analysis. Database Oxford 2014 https://doi.org/10.1093/database/bau069 (15 June 2017, date last accessed). 10.1093/database/bau069 PMC4095679 25024350

[279] Gentleman RC, Carey VJ, Bates DM Bioconductor: open software development for computational biology and bioinformatics. Genome Biol 2004;5(10):R80. 15461798 10.1186/gb-2004-5-10-r80 PMC545600

[280] Sumner LW, Amberg A, Barrett D Proposed minimum reporting standards for chemical analysis. Metabolomics 2007;3(3):211–21. 24039616 10.1007/s11306-007-0082-2 PMC3772505

[281] Gago J, Daloso DdM, Figueroa CM Relationships of leaf net photosynthesis, stomatal conductance, and mesophyll conductance to primary metabolism: a multispecies meta-analysis approach. Plant Physiol 2016;171(1):265–79. 26977088 10.1104/pp.15.01660 PMC4854675

