# Supplementary material for: From chromatogram to analyte to metabolite. How to pick horses for courses from the massive web resources for mass spectral plant metabolomics
Source: Gigascience. 2017 May 17;6(7):1–20. doi: 10.1093/gigascience/gix037 (PMC5499862; doi:10.1093/gigascience/gix037)
Supplement: GIGA-D-17-00039_Original_Submission.pdf [file gix037_giga-d-17-00039_original_submission.pdf]

GigaScience

From chromatogram to analyte to metabolite. How to pick horses for courses from the massive web-resources for mass spectral plant metabolomics

--Manuscript Draft--

**Manuscript Number:**	GIGA-D-17-00039
**Full Title:**	From chromatogram to analyte to metabolite. How to pick horses for courses from the massive web-resources for mass spectral plant metabolomics
**Article Type:**	Review
**Funding Information:**	Conselho Nacional de Desenvolvimento Científico e Tecnológico (246605/2012-0) — Mr. Leonardo Perez de Souza<br>Max-Planck-Gesellschaft — Not applicable
**Abstract:**	The grand challenge currently facing metabolomics is the expansion of the coverage of the metabolome from a minor percentage of the metabolic complement of the cell towards the level of coverage afforded by other post-genomic technologies such as transcriptomics and proteomics. In plants this problem is exacerbated by the sheer diversity of chemicals that constitute the metabolome with the number of metabolites in the plant kingdom generally being considered to be in excess of 200 000. In this review we focus on web-resources that can be exploited in order to improve analyte and ultimately metabolite identification and quantification. There is a wide range of available software that not only aids in this but also in the related area of peak alignment, however, for the uninitiated choosing which program to use is a daunting task. For this reason we provide an overview of the pros and cons of the software as well as comments regarding the level of programing skills required to effectively exploit their basic functions. In addition the torrent of available genome and transcriptome sequences that followed the advent of next-generation sequencing has opened up further valuable resources for metabolite identification. All things considered, we posit that only via a continued communal sharing of information such as that deposited in the databases described within the article are we likely to be able to make significant headway towards improving our coverage of the plant metabolome.
**Corresponding Author:**	Alisdair Robert Fernie<br><br>GERMANY
**Corresponding Author Secondary Information:**	
**Corresponding Author's Institution:**	
**Corresponding Author's Secondary Institution:**	
**First Author:**	Leonardo Perez de Souza
**First Author Secondary Information:**	
**Order of Authors:**	Leonardo Perez de Souza<br>Thomas Naake<br>Takayuki Tohge<br>Alisdair Robert Fernie
**Order of Authors Secondary Information:**	
**Opposed Reviewers:**	
**Additional Information:**	
**Question**	**Response**
Are you submitting this manuscript to a	Yes

Powered by Editorial Manager® and ProduXion Manager® from Aries Systems Corporation

special series or article collection?	
Please select an option from the menu:<br>as follow-up to "Are you submitting this manuscript to a special series or article collection?"	Functional Metagenomics
**Experimental design and statistics**<br><br>Full details of the experimental design and statistical methods used should be given in the Methods section, as detailed in our Minimum Standards Reporting Checklist. Information essential to interpreting the data presented should be made available in the figure legends.<br><br>Have you included all the information requested in your manuscript?	No
If not, please give reasons for any omissions below.<br><br>as follow-up to "**Experimental design and statistics**<br><br>Full details of the experimental design and statistical methods used should be given in the Methods section, as detailed in our Minimum Standards Reporting Checklist. Information essential to interpreting the data presented should be made available in the figure legends.<br><br>Have you included all the information requested in your manuscript?<br><br>"	Review article
**Resources**<br><br>A description of all resources used, including antibodies, cell lines, animals and software tools, with enough information to allow them to be uniquely identified, should be included in the Methods section. Authors are strongly encouraged to cite Research Resource Identifiers (RRIDs) for antibodies, model organisms and tools, where possible.<br><br>Have you included the information requested as detailed in our Minimum Standards Reporting Checklist?	No

Powered by Editorial Manager® and ProduXion Manager® from Aries Systems Corporation

If not, please give reasons for any omissions below.<br><br>as follow-up to "**Resources**<br><br>A description of all resources used, including antibodies, cell lines, animals and software tools, with enough information to allow them to be uniquely identified, should be included in the Methods section. Authors are strongly encouraged to cite Research Resource Identifiers (RRIDs) for antibodies, model organisms and tools, where possible.<br><br>Have you included the information requested as detailed in our Minimum Standards Reporting Checklist?<br><br>"	Review article
**Availability of data and materials**<br><br>All datasets and code on which the conclusions of the paper rely must be either included in your submission or deposited in publicly available repositories (where available and ethically appropriate), referencing such data using a unique identifier in the references and in the "Availability of Data and Materials" section of your manuscript.<br><br>Have you have met the above requirement as detailed in our Minimum Standards Reporting Checklist?	No
If not, please give reasons for any omissions below.<br><br>as follow-up to "**Availability of data and materials**<br><br>All datasets and code on which the conclusions of the paper rely must be either included in your submission or deposited in publicly available repositories (where available and ethically appropriate), referencing such data using a unique identifier in the references and in the "Availability of Data and Materials" section of your manuscript.<br><br>Have you have met the above requirement as detailed in our Minimum	Review article

Standards Reporting Checklist?<br><br>"	

Powered by Editorial Manager® and ProduXion Manager® from Aries Systems Corporation

Manuscript

Click here to download Manuscript GigaScienceReview_Submit.docx

Review:

From chromatogram to analyte to metabolite. How to pick horses for courses from the massive web-resources for mass spectral plant metabolomics

Leonardo Perez de Souza[1*], Thomas Naake[1], Takayuki Tohge[1,] and Alisdair R. Fernie[1*]

[1] Max-Planck-Institute of Molecular Plant Physiology, Am Mühlenberg 1, 14476 Potsdam-Golm, Germany

* Correspondence: LPerez@mpimp-golm.mpg.de; fernie@mpimp-golm.mpg.de

Abstract

The grand challenge currently facing metabolomics is the expansion of the coverage of the metabolome from a minor percentage of the metabolic complement of the cell towards the level of coverage afforded by other post-genomic technologies such as transcriptomics and proteomics. In plants this problem is exacerbated by the sheer diversity of chemicals that constitute the metabolome with the number of metabolites in the plant kingdom generally being considered to be in excess of 200 000. In this review we focus on web-resources that can be exploited in order to improve analyte and ultimately metabolite identification and quantification. There is a wide range of available software that not only aids in this but also in the related area of peak alignment, however, for the uninitiated choosing which program to use is a daunting task. For this reason we provide an overview of the pros and cons of the software as well as comments regarding the level of programing skills required to effectively exploit their basic functions. In addition the torrent of available genome and transcriptome sequences that followed the advent of next-generation sequencing has opened up further valuable resources for metabolite identification. All things considered, we posit that only via a continued communal sharing of information such as that deposited in the databases described within the article are we likely to be able to make significant headway towards improving our coverage of the plant metabolome.

Keywords: Arabidopsis, bioinformatics, crop species, GC-MS, LC-MS, peak identification, peak annotation.

Background

Metabolomics emerged in the late 1990s with the term coined in a review of Steven Oliver [1]. However, the 2000 paper by Fiehn and co-workers wherein gas chromatography (GC) coupled to mass spectrometry (MS) defined the chemical composition of a morphological and metabolic mutant of the model plant *Arabidopsis thaliana* [2] in doing so they were able to describe changes in the level of 326 analytes. This work thus greatly extended on the early metabolite profiling study of Sauter et al. [3] , which presented the technology as a means of putative classification of mode-of-action of pesticides. Thus the advent of metabolomics in plants arguably preceded that in microbes and mammals although the approach was rapidly adopted in these communities also [2, 4-6]. During the next two decades metabolomics had one considerable advantage over profiling technologies such as transcriptomics and proteomics in that it is not directly reliant on the genome sequence and during this time the species scope of metabolomics rapidly expanded such that it was no longer merely a tool for identifying biomarkers of cellular circumstance but additionally one of the cornerstones of systems biology and an approach which could provide mechanistic insight into metabolic regulation [7-11] . This advantage has subsequently disappeared following the widespread adoption of next-generation sequencing and the lack of linear relationship between the genome and the metabolome now represents part of the problem in identification of unknown analytes [12] . This is nicely exemplified by the fact that computation of the size of the metabolome on genome information as attempted by Nobeli and co-workers in 2003 for the E. coli metabolome and [13] rendered values far smaller than the number of metabolites actually measured to date [14]. Whilst the size of the metabolome for prokaryotes has been estimated at a couple of thousand, that of the plant kingdom dwarves these numbers with estimates ranging between 200 000 and 1 million metabolites [15]. Within the last two decades metabolomics has been employed to address a wide range of important questions in plant biology including pathway structure [15], the influence of metabolism on growth [8, 16], plant ecology [17], various aspects of plant genetics including evolution and the domestication syndrome [18-20] as well as detailed characterizations of the metabolic response to biotic and abiotic stressors [21, 22].

In this review, we discuss two topics. The first is the availability of tools to aid in chromatogram evaluation. Since we last reviewed this in 2009 [23], the number of resources has exploded as has their diversity in type. In 2009 a number of pathway, analytical standards, analytical samples and literature databases were available. In the intervening period additional sites providing information on experimental and *in silico* mass fragmentation, isotopic labeling, pathway predicted metabolites, integration of metabolomics with other platforms and mass spectrometry imaging have become available. For each resource we will briefly outline functionality and provide illustrative examples of their utility. The second is to review the current status of the broad variety of plant metabolomics databases. In this respect we list sources of archived data and their respective volumes of data. We also briefly discuss recent meta-analysis which illustrate

that despite current hurdles regarding comparability of data there is great potential for cross-study comparisons on metabolite responses in determining common responses between either genetic or environmental perturbations of metabolism. Finally, we will provide an outlook as to how the grand challenge of comprehensitivity will best be met and how the power of archived plant metabolic responses best exploited in the future.

It is not the scope of this review to discuss the theoretical details of every procedure or to document the subtle differences between the many similar tools referred to here. We rather aim to provide a general idea of the importance and challenges of each step in the metabolomics workflow and to summarize the major functions of each tool while referring to the more comprehensive literature supporting them. We attempt to classify all the resources in a simple and logical manner in order to facilitate understanding of the main functionalities of each one. It is, however, important to mention that while few of the tools presented here provide a complete workflow, most of them are able to perform multiple complementary functions somewhat blurring any initiative to accord their functions specific classifications. Other important information that we include here is how these tools can be accessed. This is usually performed either via command-line-or graphical-user-interface (GUI), the former provides flexibility and facilitating integration, automation and development, while the latter was developed to be intuitive and friendly for unexperienced users. All these features considered allow the researcher to access the information required to choose the “winning horse” under the conditions or “course” in which they are racing. Finally it is also important to highlight that these tools are constantly being update, integrated and discontinued, and while we ensured that all the links provided here were functioning at the time of writing, it is impossible to ensure that to be the case in the future.

Sample preparation and data acquisition

The metabolomics workflow (Figure 1) starts with sample preparation including extraction and often coupled to pre-treatment and chemical derivatization, followed by data acquisition which will depend on the chromatographic system, ionization source and analyzer. Optimization of sample preparation and data acquisition can considerably improve the analysis and is particularly interesting for plant metabolomics where matrix complexity is very high; nevertheless this step is often skipped over in favor of standardization and simplicity which allow for greater sample throughput. Methods for chromatography mass spectrometry based optimization are well developed and usually rely on statistical designs collectively known as Design of Experiments (DoE) [24].

While some studies have detailed its application in plant metabolite extraction [25] and liquid chromatography (LC) analysis [26], very few software tools were developed so far focusing on this kind of approach for metabolomics data. That said a couple of interesting software are MUSCLE [27], a tool for the automated optimization of targeted LC-MS/MS analysis that was shown to significantly shorten analysis times and increase analytical sensitivities of targeted metabolite analysis, and FragPred [28], which uses experimental

fragmentation from a database to select common fragmentation products that minimize uncertainty about metabolite identities in large-scale MRM experiments, have been published and appear to be highly promising.

Data processing

Raw mass spectrometry chromatograms are three dimensional data consisting of a distribution of m/z intensities over the time. Processing this data requires filtering, detecting and integrating relevant features, aligning signals across different samples, extracting compound mass spectra and normalizing the data, all with the final goal of simplifying and hence facilitating data interpretation.

Feature detection and peak alignment are the initial steps for extracting information from raw data and corresponds to the process in which relevant signals are identified and quantified across samples, having peak alignment as one of the big challenges to overcome, particularly for LC-MS where retention time is more prone to fluctuations in relation to GC-MS. The many different approaches available to perform these steps of data processing were recently reviewed by [29, 30], and some of the most popular algorithms for feature detection and peak alignment were compared in different works [31, 32]. Most software somehow integrate both steps in the same pipeline to generate a report of signal intensities over samples from raw data, and many of them also include some resource for data analysis and peak annotation that will be discussed later in more detail. In the following section we will detail the available tools for this step, adopting a similar approach in all subsequent sections also (the details of the programs are all given in additional file 1). MetAlign [33] is a versatile tool that performs well with both LC-MS and GC-MS and allows direct conversion from and to vendor formats while most other tools need an extra software for this step. It additionally provides a series of functionalities through other tools that are developed by the same group and integrate directly in the output of MetAlign. XCMS appears to be the most cited software for LC-MS data processing, it was developed for R and implements different algorithms for feature detection and alignment suitable for different kinds of data, while it can be argued that the software requires familiarity with programming and lacks resources for simple data inspection, its platform is, nevertheless, powerful and easily integrated with other tools and its extensive community of users provide a great resource for troubleshooting Moreover, a great number of other tools are built upon the functions of XCMS [34]. Amongst these, TracMass 2 [35], a MATLAB software which provides a GUI in a modular suite, was developed to provide immediate graphical feedback of every step of the processing pipeline, its benchmark paper compared the complexity of different algorithms highlighting the importance of low complexity when dealing with large data files and demonstrating it to be more efficient than MZmine 2 (see below for discussion of this software) and comparable to XCMS, two of the most popular current data processing tools. The particularities of TracMass algorithm makes it more suitable for detecting mass traces in

the low mass region that can be missed by other approaches, iMet-Q [36], a C# software with a GUI whose algorithm includes automatic detection of charge state and isotope ratio of detected peaks and was developed to minimize the amount of necessary input parameters significantly facilitates the pipeline for new users. GridMass [37] is a 2D feature detection algorithm implemented in MZmine 2 that is faster than other algorithms and potentially improves detection of low-intensity masses. MSFACTs [38], was one of the first tools developed for peak alignment, it uses peak tables or raw data in the ASCII format as input being limited only to the chromatographic domain, this approach can, however, now be considered outdated when compared with many other resources currently available. MET-IDEA [39] is a more recent and flexible tool, developed by the same group as MSFACTs, for feature detection and alignment with a friendly interface developed in .NET platform. Its features include visualization of integrated peaks and manual integration and display of mass spectra, which can be very helpful for quick data inspection. EasyLCMS [40] is a web application tool with focus on calibration and calculation of targeted metabolite concentration in terms of µmol using algorithms developed for MZmine 2. IDEOM [41] is a metabolomics pipeline using functions from XCMS and MZmatch from an Excel GUI.it also includes automated annotation based on an internal database of exact mass and retention time that can be update by users according to the machine. Massifquant [42] is a feature detection algorithm integrated into XCMS based on a Kalman filter for the detection of isotope trace, this approach was shown to be particularly useful for low-intensity peaks. MET-COFEA [43] is a C++ software accessed via a GUI that implements a novel mass trace based extracted-ion chromatogram extraction that copes better with drifts in the mass trace. It additionally uses compound-associated peak clusters instead of individual features for alignment (this clustering process is an important step to extract metabolite information and simplify data as it will be discussed below). MET-Xalign [44] is an extension for MET-COFEA that can potentially align compounds of samples from different experiments, a hard task for metabolomics datasets that is not approached by most other tools. apLCMS [45], is an R package for high mass accuracy LC-MS, which tries to be user friendly by providing a file-based operation and a wrapper function for a single command line batch process of LC-MS data, however, still requires quite some computational knowledge to operate. xMSanalyzer [46] is an R package for improving feature detection that integrates with existing packages such as apLCMS and XCMS, it systematically re-extracts features with multiple parameter settings and merges data to optimize sensitivity and reliability. Yamss [47] is a recently developed R package focused in providing high-quality differential analysis implementing a method based on bivariate approximate kernel density estimation for peak identification. In addition to the tools mentioned above there are a few tools for data processing that exclusively perform peak detection or alignment such as peak-grouping-alignment [48], an approach where information from grouping peaks within samples improve alignment across samples, and PTW [49] a fast alignment algorithm based on a variation of parametric time warping working on detected features rather than on complete profile data. In addition, cosmiq

(http://www.bioconductor.org/packages/devel/bioc/html/cosmiq.html) is a peak detection algorithm to improve detection of low abundant signals that can be easily integrated with XCMS. These algorithms represent an important effort in improving the existing approaches but they are much less accessible since they need to be integrated with other tools that usually perform similar functions and in some instances this requires quite advanced computational skills.

It is important to note the significant differences between GC-MS and LC-MS which are intrinsic to the features of each system, and can be summarized as a much higher efficiency and stability in GC over LC separation followed by a very stable fragmentation in traditional GC ion sources in contrast with the typical atmospheric pressure ionization employed with LC. This significantly influences the processes of peak alignment and spectra annotation, and while most of the tools developed with a focus towards LC-MS can also be used for processing GC-MS data, there are many developed with a particular focus on processing GC-MS data, making use of different strategies for peak alignment and integrating metabolite annotation by matching spectra to libraries. AMDIS [50], developed with the support of U.S. Department of Defense, is one of the most popular GC-MS processing tools, it automatically extracts component mass spectra from GC-MS data and uses it for search in mass spectral libraries, a disadvantage of this software is that the output requires extensive treatment to be used for further analysis. However Metab [51], an R package based on functions of XCMS was developed to automate the pipeline for analysis of GC-MS data processed by AMDIS dealing with the issue of its output data. MetaQuant [52] is a tool that uses retention index to define metabolites but it depends on other deconvolution software like AMDIS to extract mass spectra. Both MetaboliteDetector [53]and TagFinder [54] provide an efficient pipeline performing deconvolution, peak detection, compound identification, alignment based on Kovats retention index using alkane mix and quantification, and provide an interactive user interface facilitating use by unexperienced users. They do however require several manually input and data check steps that are time consuming and negate truly high throughput. TargetSearch [55] uses similar approaches to process data, identify and quantify targeted metabolites based on retention time index and spectra matching of multiple correlated masses but it is highly automated and efficient thus allowing the processing of large sample sets. PyMS [56] is an alternative to the previously mentioned interactive software, providing similar functions but being particularly suitable for scripting of customized processing pipelines and for data processing in batch mode working in Python. MET-COFEI (http://bioinfo.noble.org/manuscript-support/met-cofei/) uses reconstructed compound spectra instead of individual peaks to align signals across samples, which is expected to improve peak information for downstream analyses, it also match spectrum against an user-specific library. TNO-DECO [57] uses a segmentation approach to allow the performance of simultaneous deconvolution of multiple chromatographic MS files in a semi-automated fashion in MATLAB, thereby eliminating peak alignment. By contrast, MetaMS [58] is a pipeline for high-throughput GC-MS processing based on XCMS for peak detection and

alignment and CAMERA for compound spectra extraction which is annotated based on match with a database, this tool may be convenient for users that already implement XCMS analysis of other data, but this kind of processing is not optimal for GC-MS when compared with other processing types. Maui-VIA [59] implements a graphical interface that facilitates visual inspection of identifications and alignments providing faster interaction with the data. eRah [60] is an R tool that integrates a novel spectral deconvolution method using multivariate techniques based on blind source separation, alignment of spectra across samples without the need of internal standards for calculating retention indexes, quantification, and automated identification of metabolites by spectral library matching, in a fully automated pipeline, even though internal standards are not necessary they are still recommended to increase reliability in metabolite identification. The software ADAP-GC 3.0 [61] uses a deconvolution algorithm based on hierarchical clustering of fragment ions, the updated version is incorporated into the MZmine 2 platform and addressed issues from the first version such as fragment ions that are produced by more than one co-eluting components, and improved sensitivity and robustness. Finally, MetPP [62] is a processing tool that includes normalization and statistical analysis but is directed towards data emanating from GC×GC-TOF MS system.

Extracting compound mass spectra is another important step of data processing that reduces data complexity by many orders of magnitude by identifying m/z signals that belong to the same compound and provide essential information for further metabolite annotation through the reconstructing of mass spectra. While this process is usually integrated in GC-MS tools for feature detection, alignment and annotation, as mentioned above, there are many approaches to deal with LC-MS data such as the ones employed by CAMERA [63] a package developed in R to extract compound spectra, annotate isotopes and adducts, and propose compound mass as an extension to XCMS, it is easy to use in combination with this software and provides a significant reduction on data complexity. AStream [64] is another R package very similar to CAMERA but using a simpler algorithm for grouping the peaks. ALLocator [65], is a web based workflow that applies centwave from XCMS for feature detection followed by spectra deconvolution either by CAMERA or by the ALLocatorSD algorithm which is optimized for dealing with the particularities of ^{13}C labeled data by grouping mirrored isotopes (lighter isotopologues from feeding experiment). MSClust [66], has the same general features as the others but it was developed in the C++ language and it is optimized to work with the output files of MetAlign. RAMClustR [67] was developed in MATLAB and implemented in R, accepting directly the output of XCMS. The authors suggest the use of a workflow consisting of data acquisition under both low and high collision energy as a way to improve the quality of the spectra generated by feature clustering and provide a data format that can be submitted directly to the MassBank Database and NIST MSSearch program. By contrast, RAMSY [68] uses average peak ratios and their standard deviations rather than correlation to allow the recovery of compound spectra, the performance of this approach is typically better than the results from correlation methods, furthermore, the

script for MATLAB is available or it can be run from a web interface with a .csv table as input.

The last step of data processing, data normalization, is essential for further data analysis in order to remove bias introduced by sample preparation from meaningful biological variation. Most methodologies rely either on the use of internal standards statistical means for normalization. Most data normalization procedures are usually integrated in data analysis tools, but there are few examples of more specialized tools such as MetTailor [69] that uses a dynamic block summarization method for correcting misalignments reducing missing data and apply an RT-based local normalization procedure, or Normalyzer [70] that uses twelve different well known normalization methods and compares the results based on different parameters. IntCor [71] that corrects for peak intensity drift effects based on variance analysis, MetNormalizer [72] which allows normalization and integration of multiple batches in large scale experiments using support vector regression, and EigenMS [73] which detect bias trends in the data and eliminates them using single value decomposition are also highly useful. All of these software are implemented in R, however, with the exception of Normalyzer which can be also used in a web interface they all require considerable familiarity with this programing language.

A common feature of mass spectrometry data is the presence of multiple peaks for individual fragments resulting from the distribution of natural isotopes which are particularly interesting and explored in stable isotope labeling experiments. There are a few tools for correcting and extracting label enrichment from processed data such as Corrector [74], IsoCor [75] and ICT [76]. These tools are very similar all being based on the same matrix calculation. Corrector was developed to work on the output of TagFinder but data processed with most other tools can be easily arranged in a similar table format. IsoCor provides a GUI with a few different options including corrections for the label input whereas ICT includes features to process data from tandem MS. Nevertheless most data processing pipelines available are not particularly efficient for dealing with this kind of experiment, to fill this gap there are some specialized tools like mzMatch–ISO [77], integrated in the mzMatch pipeline. This software is capable of targeted and untargeted processing of labeled datasets and the output includes a set of plots summarizing the pattern of labelling observed per peak allowing users to quickly explore data. MetExtract [78] which relies on a mixture of cultures from the same organism under natural and labeled media to select signals that show a clear pattern of isotopic enrichment. However, the approach requires the labeled fraction to be fully labeled and the tracer to be highly pure to get the proper isotopic distributions. X13CMS [79] and geoRge [80], both run on the R platform using GC-MS output, the former algorithm iterating over MS signals in each mass spectra using the mass difference due to the label, while the latter uses statistical testing to distinguish Spectral peaks originated from labeled metabolites resulting in significant less false positives. The MIA program [81] detects isotopic enrichment in GC-MS datasets in a non-targeted manner, providing an easy GUI to visualize mass isotopomer distributions (MID) of

the detected fragments as barplots including confidence intervals and quality measures, tools for differential analysis of relative mass isotopomer abundance across samples and network assembly based on pairwise similarity of MID that can reveal related metabolites.

Another important feature of many mass spectrometry systems is their capability of performing tandem mass spectrometry. While this can significantly improve data in many ways, it adds another level of complexity for data processing. A very common use of tandem MS is to increase selectivity and accuracy in targeted analysis and MRMAnalyzer [82], MMSAT [83] and MRMPROBS [84] are useful tools developed for processing data from multiple reaction monitoring experiments. MMSAT [83] is a web tool that takes mzXML files as the input, it is able to automatically quantify MRM peaks but lacks metabolite identification capability. By contrast, MRMPROBS [84] detects and identifies metabolites automatically, providing a user-friendly GUI for data analysis. The algorithm has one limitation that it needs at least two transitions per metabolite in order to discriminate the target metabolite form isomeric metabolites and the background noise. Similarly, MRMAnalyzer [82] is an R tool allowing processing, alignment, metabolite identification, quality control check and statistical analysis of large datasets that transforms data in "pseudo" accurate m/z, in order to use the centwave algorithm from XCMS for peak detection. Untargeted metabolomics analysis can also take advantage of tandem MS, particularly for compound annotation, and there are few resources for dealing with the complexity of such experiments such as decoMS2 [85], an R package for deconvoluting MS2 spectra eliminating contaminating fragments without the need of sacrificing sensitivity in favor of sensibility by narrowing the window of isolation for collision-induced dissociation (CID) during data acquisition. This approach requires MS2 data to be acquired under low and high collision energies to solve the mathematical equations potentially reducing sensitivity of the method. Similarly MS2Analyzer [86] is a java software for identifying neutral losses, precursor ions, product ions and m/z differences from MS2 spectra based on a list of predefined transitions. These features are essential for structure elucidation using mass spectrometry and the software provides a fast and high-throughput platform for extracting this data. MS2LDA [87] is based on latent Dirichlet allocation (LDA), an algorithm originally used for text mining that was adapted to generate a list with blocks of co-occurring fragments and losses providing results similar to MS2Analyzer but without the need of user specified precursor/product transitions. MS-DIAL [88] and MetDIA [89] both deal with Data-independent acquisition (DIA) data, an interesting approach for untargeted metabolomics that acquire MS2 spectra for all precursor ions simultaneously with the complication that it uses larger isolation windows, hence increasing the probability of contamination in the MS2, and it loses the relation between precursor and fragment ions. MS-DIAL addresses these problems by a mathematical deconvolution based on GC-MS processing tools in a fully untargeted manner, whilst achieving the metabolite identification through a spectrum-centric library matching. By contrast, MetDIA [89] uses algorithms from XCMS for peak detection and alignment combined with a targeted approach based on

matching metabolites in a library to the detected peaks, thus achieving higher sensitivity and specificity on metabolite identification and wider metabolite coverage.

A trade-off for most of the more flexible and powerful resources presented here is that they have multiple parameters that need to be optimized, and recently a number of tools try to assist in evaluating and automatizing this process. In this context IPO [90] was developed to perform automatic optimization of XCMS parameters based on design of experiment , Credentialing Features [91] optimize detection based on regular and 13C-enriched , MetaboQC [92] is a quality control approach that evaluates alignment and suggests optimal parameters for feature detection based on discrepancies between replicate samples , and SIMAT [93] allows the selection of the optimal set of fragments and retention time windows for target analytes in GC-SIM-MS based analysis.

Data analysis

Metabolomics datasets are usually characterized by high dimensionality, heteroscedasticity (i.e. the variance in errors is not constant across the dataset) and differences of orders of magnitude across metabolite concentrations and fold changes, making it challenging to extract and visualize useful information from processed data. There are numerous approaches for data scaling, reduction, visualization and statistical analysis particularly useful for analyzing metabolomics data, many of them very well established such as analysis of variance (ANOVA), hierarchical cluster analysis (HCS), principal component analysis (PCA) and partial least squares discriminant analysis (PLS-DA) to mention just a few. There are many general statistical software capable of performing most of these functions, but also a variety of software tools exist combining procedures relevant to metabolomics in a single pipeline and thus facilitating the workflow such as DeviumWeb (https://github.com/dgrapov/DeviumWeb), BioStatFlow (http://biostatflow.org/), MetaboLyzer [94], metaP-Server [95], Fusion (https://fusion.cebitec.uni-bielefeld.de/Fusion/login) , Pathomx [96], MSPrep [97], MixOmics (http://mixomics.org/) and COVAIN [98].

Other interesting and somehow more specialized tools include RepExplore [99] which exploits information from technical replicate variance to improve statistics of differential expression and abundance of omics datasets, KMMDA [100] and Metabomxtr [101] which deal with the troublesome issue of missing metabolite values, the former through a kernel-based score test and the later through mixed-model analysis. Similarly, PeakANOVA [102] identifies peaks that are likely to be associated with one compound and uses them to improve accuracy of quantification, a particularly useful approach for experiments with limited sample size. SPICA [103], is a tool that aims at extracting relevant information from noisy data sets by analyzing ion-pairs instead of individual ions. MetabR [104], normalizes data using linear mixed models and tests for treatment effects with ANOVA. By contrast MPA-RF [105] combines random forests with model population analysis for selecting informative metabolites. Qcscreen [106], helps to verify data consistency, measurement

precision and stability of large scale biological experiments. The program SpectConnect [107] identifies conserved metabolites in GC-MS datasets. Finally,MathDAMP [108], a Mathematica package for Differential Analysis of Metabolite Profiles highlights differences within raw LCMS and GCMS dataset.

Metabolite annotation

Metabolite annotation is often considered the most challenging step and as such represents a major bottleneck for metabolomics studies. Even though the gold standard for structural characterization remains NMR characterization of the pure compound [109, 110], MS based metabolomics offers many advantages including lower cost, higher sensitive and throughput, and it can be easily hyphenated with chromatography while still providing considerable structural information. As a consequence great efforts have been made to improve mass spectrometry based metabolite annotation, and a battery of interesting tools were developed with this goal in mind. Structural information is normally extracted from mass of molecular ion in high-resolution MS (HRMS) which can provide the molecular formula and fragmentation pattern. It is important to note that most strategies for metabolite annotation rely heavily on information retrieved from databases of molecular formulas, spectra and pathways which will be discussed in more detail below.

The most common tools are based on matching spectra or exact masses from unknown compounds against spectral data deposited in some database. One example using this approach is MetaboSearch [111], which accepts either a list of m/z or the output of CAMERA as input and searches against four major metabolite databases, Human Metabolome DataBase (HMDB), Madison Metabolomics Consortium Database (MMCD), Metlin, and LipidMaps. Similarly, PUTMEDID-LCMS [112] developed in the Taverna Workflow Management System, also integrates a step of compound mass spectra extraction to define a molecular formula from high resolution m/z that is then matched against a predefined list of molecular formulas to annotate compounds. MetAssign [113] is integrated in mzMatch and it considers the uncertainty related with metabolite annotation using a Bayesian clustering approach to assign peak groups, this approach has the advantage of providing a quantitative values for uncertainty/confidence in the outputs that can be used in further analysis. The program SIRIUS [114] is a Java-based software that combines high accuracy mass with isotopic pattern analysis to distinguish even molecular formulas in higher mass regions. Furthermore it also analyses the fragmentation pattern of a compound using fragmentation trees that can be directly uploaded to CSI:FingerID (described below) via a web service. MFSearcher [115] is a tool that efficiently searches high accuracy masses against a database of pre-calculated molecular formulas with fixed kinds and numbers of atoms that are further queried against different databases, HR3 [116] is a similar tool for molecular formula calculation and query in external databases. It uses different sets of rules for heuristic filtering of candidate formulas instead of a pre-calculated database which makes it slightly slower than MFSearcher, but HR3 includes compounds with atoms that are

not present in MFSeacher's list as well as considering matches to the isotopic pattern within its annotations. Another level of biologically relevant information is added by many tools that incorporate pathway information to assist annotation and interpretation of results such as Metabolome searcher [117], a web-based application to directly search genome-constructed metabolic databases which includes MetaCyc with data on plant metabolism. MassTRIX [118] is a web interface that takes a mass peak list from HRMS as input and matches them against KEGG compounds database returning a pathway map with the matches, organisms can be selected and the output represents organism-specific and extra-organism items differentially colored to assist interpretation. MetabNet [119] is an R package to perform targeted metabolome wide association study of specific metabolites, this approach uses the correlation of all mass signals with the targeted metabolite across samples to build networks that can be visualized in pdf or exported to Cytoscape. This can be a very useful approach to identify related compounds and associate them to metabolic pathways. Similarly, ProbMetab [120] is an R package for probabilistic annotation of compounds based on the method developed by Rogers et al. (2009) [121] that incorporates information on possible biochemical reactions between the candidate structures to assign higher probabilities to compounds that form substrate/product pairs within the same sample. MI-Pack [122], implemented in python, calculates differences in mass between all molecular formulas annotated from HRMS and compares them to known substrate/product pairs from KEGG, but matches are considered based on the error between experimental and theoretical masses compared to a threshold defined by a calculated mass error surface. PlantMAT [123] is a particularly interesting tool specifically for the investigation of plant specialized metabolism, which uses an approach based on common metabolic building blocks to predict combinatorial possibilities of phytochemical structures used for annotation and as such is a highly effective way to search the chemical space surrounding a (set of) metabolite(s)

Another more recent and promising approach made possible by the huge amount of data available uses algorithms, mostly based on machine learning, to predict molecular properties of unknown compounds from its tandem mass spectra. All the tools listed below provide similar web interfaces for putative metabolite identification differing mainly on the algorithms used to perform the identification and the overall performance. MetFrag [124] retrieves candidate structures either from databases based on exact mass or from user specified SDF files, fragments them using a bond dissociation approach and compares the fragments with the input spectra scoring matches based on a series of rules. The candidates can also be filtered to facilitate the analysis based on relevant factors such as metabolite origin, composition, LC retention time and metadata from the databases. Besides the Java web-interface a command line version and an R package are provided which are more suitable for batch processing and integration with other tools. In a very similar approach MolFind [125] retrieves candidates from databases based on exact mass, filters them by comparing experimentally measured retention index, ECOM50 (the energy in eV required to

fragment 50% of a selected precursor ion) and drift time (for ion mobility MS) with predicted ones, and analysis CID of the best candidates using MetFrag. CFM-ID [126] is based on competitive fragmentation modeling, a probabilistic generative model that uses machine learning to learn its parameters from data. It can be used to predict spectra of known chemical structures, to annotate peaks in the spectra of a known compound or to predict candidate structures for an unknown compound by ranking candidates in terms of how closely the predicted spectra match the input. MAGMa [127], extends prediction based on substructure assignment by creating hierarchical trees of predicted substructures capable of explaining MS^n data, where each level takes into account the restrictions imposed by the assignment of precursor and subsequent fragmentation. FingerId [128] developed a model based on a large dataset of tandem MS from MassBank and uses a support vector machine to predict the molecular fingerprint of the unknown spectra and compare this with the fingerprint of compounds in a large molecular database. CSI:FingerID [129] is a more recent tool based on fingerID that includes computation of fragmentation tree achieving the best search performance so far. Besides the web interface it can be also queried directly through Sirius but it currently does not support batch mode. Finally MetFusion [130] is a Java web tool that combines spectra database matching against MassBank with the prediction based annotation provided by MetFrag.

Data interpretation

Interpretation of omics data is usually complicated by the amount and complexity of data. There are many tools to assist metabolomics data interpretation, particularly for its visualization by mapping metabolites into pathways and providing biological context, and for the integration with data from different platforms (e.g. transcriptomics, proteomics see Tohge et al. (2015) [15] for details). As for metabolite annotation, these tools usually rely upon knowledge stored in metabolite and pathway databases, and many of them include some kind of statistical analysis such as pathway enrichment and correlation analysis.

Visualization tools provide a simple mean of representing and mapping metabolic changes in tools like PATHOS [131], PathWhiz [132] and iPath [133]. They can often provide some kind of pathway structure analysis such as PathVisio [134], FunRich [135], BiNChE [136] and MPEA [137] that uses pathway enrichment analysis and PAPi [138] that calculates pathway activity scores to represent the potential metabolic pathway activities, and performs statistical analysis to investigate differences in activity between conditions. Tools like InCroMAP [139], IIS [140], KaPPA-View4 [141], MapMan [142], ProMeTra [143] which is integrated with MeltDB 2.0, Paintomics [144], VANTED [145], MBROLE [146] and IMPaLA [147] go one step further and integrate metabolomics processed data with other omics platforms, particularly transcriptomics, providing analysis and visualization of large integrated datasets to assist data interpretation.

Few tools try to actually use mass spectra features to build the networks, which can also improve annotation of unknown compounds. MetaNetter [148] uses raw high-resolution

data and a list of potential biochemical transformations to infer metabolic networks. MetaMapR [149] builds chemical and spectral similarity networks based on annotated and unknown compounds. ChemTreeMap [150] uses annotated structures and a computational approach to produce hierarchical trees based on compound similarity to assist visualization of chemical overlap between molecular datasets and the extraction of structure–activity relationships. MetFamily [151], groups metabolites in families based on an integrated analysis of MS1 abundances and MS/MS facilitating further data interpretation. MetCirc (https://www.bioconductor.org/packages/release/bioc/html/MetCirc.html) is an R tool particularly useful for comparative analysis from cross-species and cross-tissue experiments through computation of similarity between individual MS/MS spectra and visualization of similarity based on interactive graphical tools, and TrackSM [152] is a Java tool that uses molecular structure similarities to assign newly identified biochemical compounds to known metabolic pathways.

Databases

It must be clear from previous sections that mass spectrometry based metabolomics, particularly metabolite annotation and data interpretation, relies heavily upon data from characterized mass spectra, molecular properties of analytes and metabolic pathways. While all the different techniques offer a lot of flexibility, metabolomics struggles with standardization and a great volume of metadata when compared with other omics techniques and still lags behind most of them in terms of public repositories of published data. Nonetheless there are a wealth of databases with useful information for mass spectrometry based plant metabolomics and we try to summarize some of the most relevant and the structure and functionalities of resources available.

Chemspider [153], PubChem [154], ChEBI [155], ChEMBL [156],ChemBank [157], HMDB [158], MMCD [159] and MMsINC [160] are all large databases of small molecules with information such as chemical structure, molecular formula and molecular/exact mass, many of these databases complement each other and data exchange between them is very common, nevertheless it is important to be aware of the sources of data in each one of them and to which extent these data is curated, Chemspider for instance has more than 58 million structures automatically retrieved from over 450 different sources, with only a fraction of this being manually curated by registered users while the majority of data only went throught some sort of automatic curation and elimination of redundant entries. Overall such huge databases are particularly useful for looking for physico-chemical properties of identified metabolites and checking for possible candidates based solely on their mass.

There are a few plant specific databases with curated information on chemical composition and distribution across different plant species as well, namely KNApSAcK [161] with information of more than 50,000 metabolites, chemical composition of over 22,000 species, Flavonoid viewer [162] with 6,902 molecular structures of flavonoids from 1,687 plant

species, Dr. Duke's Phytochemical and Ethnobotanical Databases (https://phytochem.nal.usda.gov/phytochem/search) with information on 29,585 chemicals of 3,686 medicinal plants, BioPhytMol [163] a resource on anti-mycobacterial phytomolecules and plant extracts holding 2,582 entries including 188 plant families, comprised of 692 genera and 808 species, and 633 active compounds and plant extracts identified against 25 target mycobacteria, and EssOilDB [164] with 123,041 essential oil records from 92 plant families. These are very interesting resources for screening chemical composition of specific species and analyzing chemical distribution species wide, and all of the data in these databases is manually curated. From all this resources KNApSAcK is particularly useful not only for the larger amount of data but also for providing an easy platform to access and extract information quickly.

Databases providing mass spectra of pure compounds under controlled conditions developed to allow search for common spectra features for the identification of unknown compounds are an essential resource for MS based identification of metabolites. As previously mentioned the great stability and reproducibility of GC-MS generates reliable fragmentation patterns and relative retention indexes that are very efficient for metabolite annotation by spectra matching. NIST is a very popular commercial library for GC-MS annotation, that also provide free access to some data throught NIST Chem WebBook (http://webbook.nist.gov/chemistry/), containing mass spectra of 33,000 compounds. SDBS (http://sdbs.db.aist.go.jp/sdbs/cgi-bin/cre_index.cgi) with 25,000 mass spectra is the database from the National Institute of Advanced Industrial Science and Technology (AIST) from Japan. Both of them are limited in the fact that they do not offer an interface for spectra matching and the user have limited access to data, so those are only useful for checking the spectra of targeted compounds. Some more interesting freely-accessible plant specific GC-MS libraries include the Golm metabolome database [165] with a total of 26,590 spectra and 4,663 analytes at the time this article was written and the VocBinBase [166] includes 1,537 unique mass spectra at the time this article was written. Both of these databases can be downloaded and integrated to processing tools for metabolite annotation based on spectra matching.

One of the greatest efforts in the field of metabolomics has been directed to the development of databases of mass spectra obtained from LC-MS analysis. The higher flexibility of this technique compared to GC-MS in terms of the chemical space that it can analyze comes with the drawback of a high sensitivity to multiple factors that can influence mass spectra quality and reproducibility. LC-MS databases are usually characterized by the greatest volume of metadata that accompanies the analytical data, and a more complex structure for search based in spectra features when compared to GC-MS databases. Some large general LC-MS databases include MassBank [167], a public repository of mass spectra with 41,092 spectra of 15,828 compounds obtained by 26 different systems (at the time of writing). This database is very accessible allowing search by submitted spectra or simply by typing in spectral features, mass or targeted compound name, it furthermore allows users

to directly extract spectra during data processing through many tools like RAMClustR, RMassBank and Mass++. METLIN [168] currently containins 961,829 molecules from which 200,000 have in silico MS/MS data, and 14,000 mass spectra at multiple collision energies in positive and negative ionization mode. METLIN also integrates isoMETLIN [169] that allows the search of isotopologues for all METLIN metabolites based on m/z and isotopes of interest, and includes experimental data on hundreds of isotopic labeled metabolites that can be used to obtain information of precursor atoms in the fragments, both databases can be accessed after free registration and searching by mass is fast and easy with the advantage that it allows the user to select possible adducts and spectra conditions and search directly the mass observed in the spectra. T3DB [170], is a database for toxin data, many of which are plant secondary metabolites, with MS, MS-MS and GC-MS spectra of 3,600 common toxic substances (at the time of writing). mzCloud is a new database with a more complex organizing structure that can improve and facilitate data interpretation, currently with 6,255 compounds analyzed in different conditions totalizing 1,913,621 spectra arranged in 9,896 tree structures. It allows the user to easily navigate through different spectra of a single compound through its tree structure and also includes visualization of predicted molecular formula of the fragments in the spectra (https://www.mzcloud.org/). Finally the recently developed MoNA (http://mona.fiehnlab.ucdavis.edu/) is intended to be a centralized, collaborative database of metabolite mass spectra and metadata, currently containing over 200,000 mass spectral records from experimental and in-silico libraries from different sources. The search is limited to name, compound class, molecular formula or exact mass of the metabolite, it can be filtered by type of spectra, and the results are presented as a single list of individual interactive spectra next to the metadata making it easy to navigate through different spectra. The great diversity of phytochemicals observed in plants represent an important portion of all these numbers, and a few plant specific databases are available such as Spektraris [171], a LC-MS of about 500 plant natural products that integrates accurate mass – time tag to incorporate retention time relative to an internal standard in a similar fashion as it is usually done for GC-MS based annotation, therefore, in order to use this feature it is necessary to analyze samples with addition of the same internal standard used when developing the database entries. It is important to highlight that this kind of approach is much less effective for LC-MS where relative retention time is prone to larger variation. MS-MS Fragment Viewer (http://webs2.kazusa.or.jp/msmsfragmentviewer/) is a very small and not very frequently updated database containing FT-MS, IT- and FT-MS/MS spectral data on 116 flavonoids. ReSpect [172] is a collection of MSn spectra data from 9,017 phytochemicals from literature and standards with searching functionalities very similar to MassBank, and WEIZMASS [173], a metabolite spectral library of high-resolution MS data from 3,540 plant metabolites that uses a probabilistic approach to match library and experimental data with the MatchWeiz software. WEIZMASS is available for implementation in R as a pipeline for metabolite identification which can be easily integrated with data processing. While this is a much less accessible tool for general use compared with other web based databases the

results obtained are far more considerable and the effort required in its use is, therefore, more than compensated by the gains which it affords.

A very common issue encountered in data from mass spectrometry is the presence of a variety of contaminants from sample preparation and analysis that can be challenging for data interpretation. MaConDa [174] provides a very useful database of common contaminants and adducts in mass spectrometry, containing over 200 contaminant records with origin of the contaminant, its mass and the adducts formed. MaConDa can be downloaded in different formats or accessed via the web browser.

Compound spectra databases are essential for identification of metabolites by mass spectrometry, but a significant effort has also been directed towards the development of repositories of experimental data on specific samples to facilitate dereplication studies and data analysis. These databases are often restricted to specific species, as it is the case for AtMetExpress [175], a LC-MS database of Arabidopsis with data on 20 different ecotypes and 36 developmental stages which allows users to download raw and processed data as well as query using mass chromatogram features in the web platform and visualize annotation and distribution of selected features. MeKO [176], is a GC-MS database of 50 Arabidopsis KO mutants. All raw data can be downloaded as netCDF files and results from data analysis can be visualized in a very informative summary in the web browser that shows plant phenotypes, differentially accumulated metabolites indicated in a pathway map and log fold changes for most significantly changed metabolites. MoTo DB [177] is a LC-MS database of *Solanum lycopersicum* with information of annotated metabolites where the user can search for specific masses or a range of masses. The database is based on accurate mass and the user therefore does not have access to raw data and chromatograms. NaDH [178], a platform for integration and visualization of different omics datasets of *Nicotiana attenuata* including LC-MS data on 14 different tissues, allows search for spectra based on name and m/z and provides some interesting tools for data interpretation easily accessible directly from the metabolite entry including metabolite-metabolite and metabolite-gene coexpression analysis and visualization of metabolite expression across different tissues in a bar chart or eFP browser interface. The Optimas-DW software [179], is a data collection for maize data of 15 different experiments, the interface for metabolites allows easy browsing through all the metabolites and visualization of values for individual experiments in a table format but no access to raw data, and the SoyMetDB [180], a metabolomics database for soybean, with GC-MS and LC-MS data of four different tissues under two different conditions, which has a simple interface that provide search by metabolite name or browsing through the whole dataset, metabolite entries provide m/z, retention time as well as an apparent defunct link to a pathway viewer. Similar databases with relative broader spectra include the plant specific KOMIC Maket [181] currently warehousing LC-MS data on 74 samples from 17 species, in which the user can search for peaks and browse through samples and the interface shows retention times, m/z and annotation details classifying the annotation based on a grading system. MS2T [182] is an MSMS library created using a

function for automatic Tandem MS acquisition from over 150 samples from 10 different plant species, the web platforms allows search by retention time, m/z and spectra similarity. PMR [183], is a database for plants and eukaryotic microorganisms which includes the earlier database of medicinal plants MPMR [184] and currently comprises of GC-MS and LC-MS data on 24 species from different sources and experiments including different tissues and developmental stages. It has an easy and clear interface with summary of all the experiments once an individual species is selected including metadata and annotated metabolites. It additionally allows the download of all the results in csv format in the form of peak tables and it has some basic tool for comparative analysis where volcano plots can be generated comparing different experiments. By contrast, the more general databases Bio-MassBank (http://bio.massbank.jp/), a repository of LC-MS and GC-MS data from biological samples, in contrast with the original MassBank in this database most of the data is tagged as “Unknowns” or are just putative metabolites, searching functions are similar to the original database but it includes a samples section where it is possible to access all the experiments available. MassBase (http://webs2.kazusa.or.jp/massbase/) is a large repository providing raw and processed mass chromatograms on 46,398 samples of over 40 species, including several plants, analyzed by LC-MS, GC-MS and CE-MS. Metabolomics Workbench [185] is a repository of a variety of metabolomics experiments containing over 60,000 entries, including raw and processed MS data, a section with detailed protocols for the experiments, and web tools for analysis and interpretation that can be used with any uploaded data. Similarly, Metabolights [186], is a cross species repository containing data from 190 mass spectrometry based metabolomics studies that is currently recommended as repository of experimental data by many journals, all experimental data can be downloaded from an ftp server and data submission is powered by the use of ISA software that assists in the reporting and management of metadata. MetabolomeXchange [187], is a data aggregation system that allows users to efficiently explore experimental metabolomics data from different databases including MetaboLights and Metabolomics Workbench providing an RSS feeding service to allow users to get updates over the datasets available. Similarly, GNPS [188], a plant natural product knowledge base for community-wide organization and sharing of raw, processed or identified tandem mass spectrometry data currently comprising of 221,083 MS/MS spectra from 18,163 unique compounds. The platform allows users to upload data and provides a series of tools for analysis and interpretation based on the data from the database.

As previously mentioned, many resources that are particularly useful for data interpretation organize the data in pathways based on literature data, and often also provide tools for data visualization and interpretation. Many of these databases contain either generic pathways or combine different organisms, some examples are KEGG [189], which includes 504 pathway maps with 17,891 compounds and 10,419 reactions for 4,607 different organisms, representing data in an interactive interface that links the entries to a great amount of external resources being one of the most popular sources of information on metabolic

pathways One of the greatest issues of KEGG leading many user to misinterpreting their data is that it displays all genes in generic pathway maps of which some are characterized only by similarity, resulting in pathways that are not present in the analysed organism being represented. By contrast, WikiPathways [190], is a wiki-style website with 2,471 community curated pathways of 28 different organisms. Its interactive interface is similar to KEGG providing link with external resources for metabolites and enzymes. Similarly, kpath [191], is a database that integrates information related to metabolic pathways with 74,180 pathways 13,153 reactions and 37,029 metabolites providing tools for pathway visualization, editing and relationship search. BioCyc [192], is a collection of 9,387 Pathway/Genome Databases, and MetaCyc [192] is the largest curated database of experimentally elucidated metabolic pathways containing 2,491 pathways from 2,816 different organisms. KBase [193], meanwhile, is a data platform with data on plants and microbes that allow users to upload their own data and integrates data and tools for systems biology including 1,470 metabolic pathways with 33,773 reactions and 27,838 compounds, genome data on 60 different plant species and tools for assembly, annotation, metabolic modeling, comparative analysis, phylogenetic analysis and expression analysis. There are also a significant amount of plant specific data organized in databases like KaPPA-View4 [141], containing 153 pathways with 1,427 compounds and 1,434 reaction from 10 species, allowing users to upload their own data and is able to represent gene-to-gene and metabolite-to-metabolite relationships as curves on a metabolic pathway maps to help in data interpretation. PlantCyc (http://www.plantcyc.org/) provides access to manually curated or reviewed information about metabolic pathways in over 800 pathways of 350 plant species, usefully the platform provides "evidence codes" to clearly indicate the type of support associated with each database item. MetaCrop [194], is a pathway database containing information about seven major crop plants and two model plants that allows integration of experimental data into metabolic pathways, as well as the automatic export of information for the creation of detailed metabolic models. Similarly, MetNetDB [195], contains integrative information on metabolic and regulatory networks of Arabidopsis and Soybean with metabolism, signalling, and transcriptional pathways being fully integrated into a single network and manually curated subcellular localization is represented in the pathway maps. The network information can be exported to other applications for network analysis such as exploRase, and Cytoscape/FCM. Like MetNetDB, Gramene [196] is an integrated data resource for comparative functional genomics in crops and model plants that host pathway databases for rice, maize, Bracypodium, and sorghum as well as providing mirrors for MetaCyc and PlantCyc data. It is worth mentioning a few resources that are focused on the reactions within the pathways offering detailed curated metabolic reactions, namely BioMeta [197], whose contents are based on the KEGG Ligand database with a large number of chemical structures corrected with respect to constitution and reactions' stereochemistry being correctly balanced. BKM-react [198] is a non-redundant biochemical reaction database containing 18,172 unique biochemical reactions retrieved from BRENDA, KEGG, and MetaCyc databases that were matched and integrated by aligning substrates and products.

Similar to this MetRxn [199], also integrates information from BRENDA, KEGG and MetaCyc, combining also Reactome.org and 44 metabolic models in a standardized description of metabolites and reactions where all metabolites have matched synonyms, resolved protonation states, and are linked to unique structures, and all reactions are balanced.

Together with the development of many prediction tools previously mentioned we watched in the last years the development of some interesting *In Silico* databases that are extremely useful for *de novo* metabolite identification such as MINE [200], a database developed by the integration of an algorithm called Biochemical Network Integrated Computational Explorer (BNICE) and expert-curated reaction rules to predict chemical structures product of enzyme promiscuity, MetCCS [201] a database and algorithm for prediction of Collision Cross-Section values for metabolites in ion mobility mass spectrometry, a technique increasingly used to assist metabolite elucidation based on the drift speed of the ion that is proportional to its cross section, and the plant specific ISDB [202] an *in silico* database of natural products generated using CFM-ID [126] with input from the commercial Dictionary of Natural Products.

Other programs of interest

The complexity of metabolomics data experiments, particularly in terms of sample number and metadata pushed the development of many tools for experiment and metadata management, and while many of these functions are integrated in some of the databases previously discussed there are a few specialized tools such as QTREDS [203] and MASTR-MS [204], that are LIMS based software for assisting in organizing experimental design, metadata management and sample data acquisition , MetaDB [205] a web application for Metabolomics metadata management with interface to MetaMS data processing tool, and Metabolonote [206], a metadata database/management system.

The enormous amount of data available for metabolomics raises many questions regarding how to easily access and unify all this data, taking into account the vast chemical space explored in these experiments. Many tools have been developed with the purpose of facilitating access to chemical data spread in the literature, from the development of identifiers to reduce duplication of information such as the SPLASH [207] hash designed for the MoNA database, to tools like Metmask [208], for managing different identifiers, CTS [209], for translation of chemical identifiers, PhenoMeter [210] for querying databases based on metabolic phenotype and Metab2MeSH [211] for a more efficient literature search that automatically annotate compounds with the concepts defined in MeSH providing a fast link between compound and the literature.

Different vendors usually export their data in proprietary formats which complicates data transfer across different platforms. Most proprietary software are able to convert files to .cdf format, but some tools from which the most popular is msConverter from Proteowizard (http://proteowizard.sourceforge.net/) can handle conversion from/to different formats

including mzXML. mzTab is another format proposed by the Proteomics Standards Initiative targeting researchers outside of proteomics, it is supposed to contain the minimal information required to evaluate the results of a proteomics experiment making it more accessible to non-experts, jmzTab [212] is a java application that provides reading and writing capabilities and conversion of files to mzTab. The PeakML [213] file format is an initiative developed by the creators of mzMatch to enable the exchange of data between analysis software by representing peak and meta-information from each step in an analysis pipeline, as a proof of concept the R-package 'mzmatch.R' was developed to extend XCMS functionalities for storing and reading data in PeakML format.

All equipment for mass spectrometry comes with their own software for data visualization and some basic analysis but those are usually not designed to deal with the complexities of metabolomics datasets. There are some interesting open source alternatives such as BatMass [214] and Mass++ [215] for data visualization, and for generating images from raw data like SpeckTackle [216] that provides several pre-defined chart types easy to integrate into web-facing resources and RMassBank [217] capable of automatically generating MassBank records from raw MS and MS/MS data.

Mass spectrometry imaging is a relative young technique that has being growing fast in importance providing high resolution special distribution of small molecules in molecular histology [218]. Few tools have been developed so far, namely EXIMS [219] for data processing and analysis, and OpenMSI [220], a web-based visualization, analysis and management tool.

Lipidomics data requires a very specialized pipeline and therefore many tools were developed exclusively for this kind of analysis however we will only briefly summarize these here. ALEX [221], MRM-DIFF [222], LICRE [223], LipidXplorer [224], LIMSA [225], VaLID [226], LOBSTAHS [227], Lipid-Pro [228], LDA [229] and LipidQA [230] are all tools for processing, annotating and analyzing lipidomics data. Lipids databases include LIPID MAPS [231], LIPIDBANK [232], LipidBlast [233], and in silico generated lipids database LipidHome [234], SwissLipids [235] and ARALIP (http://aralip.plantbiology.msu.edu/pathways/pathways).

Future perspectives

Many of the resources presented here were fruit of the efforts in setting the theoretical background for each step in the data processing and analysis workflow. However, more recent efforts are moving towards the development of integrated tools, which are often developed by the integration of already well established tools into a single pipeline in an attempt to accelerate the process and in a few cases providing an easier interface. XCMS online, for example, is a web platform providing most of the function from XCMS with additional capabilities for interactive exploratory data visualization and analysis in a much easier interface than the original software [236], HayStack [237], is a web platform that uses

XCMS to process data and automatically generates total ion chromatograms (TIC) and base peak chromatograms as well as offering an easy way of plotting extracted ion chromatograms (EIC) and some basic statistical tools such as PCA scores plot, volcano plots, and dendrograms for group comparisons, SMART [238] is an R package that combines different tools such as XCMS and CAMERA with a series of common statistical approaches to provide an integrated pipeline for data processing, visualization, and analysis. MZmine 2 [239] is another very popular tool with over 1000 citations, it was originally developed for LC-MS data processing but it became one of the most popular platforms for development of integrated tools in Java providing a user-friendly, flexible and extendable software constantly updated and with a set of modules covering most steps of LC-MS processing and data analysis workflow including several option of visualization tools. MetSign [240] is a MATLAB package providing tools for spectra deconvolution, metabolite putative assignment by matching m/z and peak isotopic distribution against its own database, peak list alignment, a series of normalization algorithms, statistical significance tests, unsupervised clustering, and time course analysis, all in a modular and interactive design presented with a wizard to facilitate the analysis workflow. MultiAlign [241] is a software developed in the .NET platform using C++ and C# originally for proteomics but that can also be used for metabolomics comparative analysis, its functionalities include feature detection, alignment, several plotting options, normalization, and basic statistical comparisons, Metabolome Express [242] works as a web server to process, interpret and share GC/MS metabolomics datasets, whilst MAIT [243] is an R package aiming at providing an end-to-end programmable metabolomics pipeline with emphasis in metabolite annotation and statistics, it uses XCMS for peak detection, an approach based on CAMERA combined with an user defined table of biotransformations followed by database search for metabolite annotation and a series of statistical tests to identify statistically significant features containing the highest amount of class-related information. By contrast, MAVEN [244] is a software for data processing, analysis and visualization with some interesting features for pathway-based visualization of isotope-labeling data that can be helpful for the interpretation of this kind of experiment. MeltDB [245] is a java web based platform that integrates different algorithms for data processing, compound identification by spectra matching statistical analysis, data visualization and integration with transcriptomics and proteomics datasets via the ProMeTra software. It provides a tool for saving peaks of reference compounds directly in the MeltDB database, and allows storage and sharing of projects within the web server. MetaboAnalyst [246] is another java web platform with data processing and a comprehensive set of data analysis tools, it includes most common approaches for statistical analysis as well as modules for functional enrichment analysis, metabolic pathway analysis, time series and two-factor data analysis, biomarker analysis, sample size and power analysis, integrated pathway analysis, and image and report generation. The program mzMatch [213] is a popular Java toolkit for processing, filtering, and annotation, with particular focus on integration of processed data across different platforms and providing a customizable modular pipeline to facilitate the development and

integration of different tools. It includes many other tools previously described here like mzmatchISO and metAssign and it is based entirely in the PeakML file format. The MarVis-Suite [247] is a software for the interactive ranking, filtering, combination, clustering, visualization, and functional analysis of transcriptomics and metabolomics data sets, the clustering algorithm is based on one-dimensional self-organizing maps (1D-SOMs), and the software additionaly provides functions for metabolite annotation and pathway reconstruction. MetMSLine [248] is an R package that works with processed data providing a series of statistical analysis steps focusing on biomarker discovery combined with metabolite annotation based on exact mass matching against a target list of metabolites and MassCascade [249] is a Java library that takes advantage of the KINIME workflow environment facilitating integration with other tools and making the tool user friendly, the core library contains a collection of data processing algorithms, a visualization framework and metabolite annotation functions, while the plug-in for KNIME allows easy integration with other statistical workflows. MASSyPup [250] does not actually integrate different procedures but it does provides an easy platform for accessing many different tools in the form of a Linux distribution that can be run directly from different media without installation.

It is clear from this review the infinity of choices for performing a variety of functions and the fast pace by which they change and get outdated; hence it is an arduous task to keep updated of all of them. Some research groups, engaged in the development of metabolomics tools, have their own repositories like KOMICS [251], MetaOpen (http://metaopen.sourceforge.net/) and PRIMe [252], while OMICtools [253], NAR online Molecular Biology Database Collection and the Bioinformatics Links Directory provide unified repositories but still covering only a small portion of all the resources available. With the rapid development of new tools it is of great interest for the metabolomics community to develop classification systems and repositories to catalog and provide a platform for submission, curation and feedback facilitating users' access to the most appropriate resources for each aim. Another clear observation that can be made from the proceeding sections is that the number of tools for analysis by far exceeds that of the number of data repositories whilst metabolomics is clearly difficult to fully standardize this is still a great shame. There are a number of clear reporting standards that should aid in this respect [254], furthermore, both the existing databases and carefully compared meta-analysis [22, 255], demonstrate that such approaches are indeed highly powerful in the enhancement of biological understanding. As such we feel that it is an urgent priority to focus efforts on the improvement of this feature of computational metabolomics since it will aid not only in the expansion of our coverage of the metabolite complement of the plant cell but also in the equally important task of interpreting the biological function of the individual metabolites themselves.

Competing interests

The authors declare that they have no competing interests.

Acknowledgement

We thank the Max Planck Society, the National Council for Scientific and Technological Development CNPq-Brazil (LPS) and the IMPRS-PMPG program (TN) for the financial support.

References

1. Oliver SG, Winson MK, Kell DB and Baganz F. Systematic functional analysis of the yeast genome. Trends Biotechnol. 1998;16 9:373-8. doi:10.1016/s0167-7799(98)01214-1.
2. Fiehn O, Kopka J, Dormann P, Altmann T, Trethewey RN and Willmitzer L. Metabolite profiling for plant functional genomics. Nat Biotechnol. 2000;18 11:1157-61. doi:10.1038/81137.
3. Sauter H, Lauer M and Fritsch H. METABOLIC PROFILING OF PLANTS - A NEW DIAGNOSTIC-TECHNIQUE. Abstr Pap Am Chem Soc. 1988;195:129-AGRO.
4. Dorr JR, Yu Y, Milanovic M, Beuster G, Zasada C, Dabritz JHM, et al. Synthetic lethal metabolic targeting of cellular senescence in cancer therapy. Nature. 2013;501 7467:421-+. doi:10.1038/nature12437.
5. Kell DB. Metabolomics and systems biology: making sense of the soup. Current Opinion in Microbiology. 2004;7 3:296-307. doi:10.1016/j.mib.2004.04.012.
6. Nicholson JK and Wilson ID. Understanding 'global' systems biology: Metabonomics and the continuum of metabolism. Nature Reviews Drug Discovery. 2003;2 8:668-76. doi:10.1038/nrd1157.
7. Fernie AR and Schauer N. Metabolomics-assisted breeding: a viable option for crop improvement? Trends in Genetics. 2009;25 1:39-48. doi:10.1016/j.tig.2008.10.010.
8. Meyer RC, Steinfath M, Lisec J, Becher M, Witucka-Wall H, Torjek O, et al. The metabolic signature related to high plant growth rate in Arabidopsis thaliana. Proceedings of the National Academy of Sciences of the United States of America. 2007;104 11:4759-64. doi:10.1073/pnas.0609709104.
9. Roessner U, Willmitzer L and Fernie AR. Metabolic profiling and biochemical phenotyping of plant systems. Plant Cell Reports. 2002;21 3:189-96. doi:10.1007/s00299-002-0510-8.
10. Schauer N and Fernie AR. Plant metabolomics: towards biological function and mechanism. Trends in Plant Science. 2006;11 10:508-16. doi:10.1016/j.tplants.2006.08.007.
11. Weckwerth W. Metabolomics in systems biology. Annu Rev Plant Biol. 2003;54:669-89. doi:10.1146/annurev.arplant.54.031902.135014.
12. Fernie AR and Stitt M. On the Discordance of Metabolomics with Proteomics and Transcriptomics: Coping with Increasing Complexity in Logic, Chemistry, and Network Interactions. Plant Physiology. 2012;158 3:1139-45. doi:10.1104/pp.112.193235.
13. Nobeli I, Ponstingl H, Krissinel EB and Thornton JM. A structure-based anatomy of the E-coli metabolome. Journal of Molecular Biology. 2003;334 4:697-719. doi:10.1016/j.jmb.2003.10.008.
14. van der Werf MJ, Overkamp KM, Muilwijk B, Coulier L and Hankemeier T. Microbial metabolomics: Toward a platform with full metabolome coverage. Analytical Biochemistry. 2007;370 1:17-25. doi:10.1016/j.ab.2007.07.022.
15. Tohge T, Scossa F and Fernie AR. Integrative Approaches to Enhance Understanding of Plant Metabolic Pathway Structure and Regulation. Plant Physiology. 2015;169 3:1499-511. doi:10.1104/pp.15.01006.

16. Sulpice R, Pyl E-T, Ishihara H, Trenkamp S, Steinfath M, Witucka-Wall H, et al. Starch as a major integrator in the regulation of plant growth. Proceedings of the National Academy of Sciences. 2009;106 25:10348-53. doi:10.1073/pnas.0903478106.
17. Davey MP, Burrell MM, Woodward FI and Quick WP. Population-specific metabolic phenotypes of Arabidopsis lyrata ssp. petraea. New Phytologist. 2008;177 2:380-8. doi:10.1111/j.1469-8137.2007.02282.x.
18. Beleggia R, Rau D, Laidò G, Platani C, Nigro F, Fragasso M, et al. Evolutionary Metabolomics Reveals Domestication-Associated Changes in Tetraploid Wheat Kernels. Molecular Biology and Evolution. 2016;33 7:1740-53. doi:10.1093/molbev/msw050.
19. Kliebenstein D. Advancing Genetic Theory and Application by Metabolic Quantitative Trait Loci Analysis. The Plant Cell. 2009;21 6:1637-46. doi:10.1105/tpc.109.067611.
20. Luo J. Metabolite-based genome-wide association studies in plants. Current Opinion in Plant Biology. 2015;24:31-8. doi:http://dx.doi.org/10.1016/j.pbi.2015.01.006.
21. Brotman Y, Landau U, Pnini S, Lisec J, Balazadeh S, Mueller-Roeber B, et al. The LysM receptor-like kinase LysM RLK1 is required to activate defense and abiotic-stress responses induced by overexpression of fungal chitinases in Arabidopsis plants. Molecular plant. 2012;5 5:1113-24.
22. Obata T and Fernie AR. The use of metabolomics to dissect plant responses to abiotic stresses. Cellular and Molecular Life Sciences. 2012;69 19:3225-43. doi:10.1007/s00018-012-1091-5.
23. Tohge T and Fernie AR. Web-based resources for mass-spectrometry-based metabolomics: A user's guide. Phytochemistry. 2009;70 4:450-6. doi:http://dx.doi.org/10.1016/j.phytochem.2009.02.004.
24. Hibbert DB. Experimental design in chromatography: A tutorial review. Journal of Chromatography B. 2012;910:2-13. doi:http://dx.doi.org/10.1016/j.jchromb.2012.01.020.
25. Gullberg J, Jonsson P, Nordström A, Sjöström M and Moritz T. Design of experiments: an efficient strategy to identify factors influencing extraction and derivatization of Arabidopsis thaliana samples in metabolomic studies with gas chromatography/mass spectrometry. Analytical Biochemistry. 2004;331 2:283-95. doi:http://dx.doi.org/10.1016/j.ab.2004.04.037.
26. Nistor I, Cao M, Debrus B, Lebrun P, Lecomte F, Rozet E, et al. Application of a new optimization strategy for the separation of tertiary alkaloids extracted from Strychnos usambarensis leaves. Journal of Pharmaceutical and Biomedical Analysis. 2011;56 1:30-7. doi:http://dx.doi.org/10.1016/j.jpba.2011.04.027.
27. Bradbury J, Genta-Jouve G, Allwood JW, Dunn WB, Goodacre R, Knowles JD, et al. MUSCLE: automated multi-objective evolutionary optimization of targeted LC-MS/MS analysis. Bioinformatics. 2015;31 6:975-7. doi:10.1093/bioinformatics/btu740.
28. Nikolskiy I, Siuzdak G and Patti GJ. Discriminating precursors of common fragments for large-scale metabolite profiling by triple quadrupole mass spectrometry. Bioinformatics. 2015;31 12:2017-23.
29. Katajamaa M and Orešič M. Data processing for mass spectrometry-based metabolomics. Journal of Chromatography A. 2007;1158 1–2:318-28. doi:http://dx.doi.org/10.1016/j.chroma.2007.04.021.
30. Sugimoto M, Kawakami M, Robert M, Soga T and Tomita M. Bioinformatics tools for mass spectroscopy-based metabolomic data processing and analysis. Current bioinformatics. 2012;7 1:96-108.
31. Lange E, Tautenhahn R, Neumann S and Gröpl C. Critical assessment of alignment procedures for LC-MS proteomics and metabolomics measurements. BMC Bioinformatics. 2008;9:375-. doi:10.1186/1471-2105-9-375.
32. Tautenhahn R, Böttcher C and Neumann S. Highly sensitive feature detection for high resolution LC/MS. BMC Bioinformatics. 2008;9 1:504. doi:10.1186/1471-2105-9-504.

33. Lommen A. MetAlign: interface-driven, versatile metabolomics tool for hyphenated full-scan mass spectrometry data preprocessing. Analytical Chemistry. 2009;81 8:3079-86.
34. Smith CA, Want EJ, O'Maille G, Abagyan R and Siuzdak G. XCMS: processing mass spectrometry data for metabolite profiling using nonlinear peak alignment, matching, and identification. Analytical Chemistry. 2006;78 doi:10.1021/ac051437y.
35. Tengstrand E, Lindberg J and Åberg KM. TracMass 2⍰ A Modular Suite of Tools for Processing Chromatography-Full Scan Mass Spectrometry Data. Analytical Chemistry. 2014;86 7:3435-42.
36. Chang H-Y, Chen C-T, Lih TM, Lynn K-S, Juo C-G, Hsu W-L, et al. iMet-Q: A User-Friendly Tool for Label-Free Metabolomics Quantitation Using Dynamic Peak-Width Determination. PLoS One. 2016;11 1:e0146112. doi:10.1371/journal.pone.0146112.
37. Treviño V, Yañez-Garza IL, Rodriguez-López CE, Urrea-López R, Garza-Rodriguez ML, Barrera-Saldaña HA, et al. GridMass: a fast two-dimensional feature detection method for LC/MS. Journal of Mass Spectrometry. 2015;50 1:165-74.
38. Duran AL, Yang J, Wang L and Sumner LW. Metabolomics spectral formatting, alignment and conversion tools (MSFACTs). Bioinformatics. 2003;19 17:2283-93.
39. Broeckling CD, Reddy IR, Duran AL, Zhao X and Sumner LW. MET-IDEA: data extraction tool for mass spectrometry-based metabolomics. Analytical Chemistry. 2006;78 13:4334-41.
40. Fructuoso S, Sevilla Á, Bernal C, Lozano AB, Iborra JL and Cánovas M. EasyLCMS: an asynchronous web application for the automated quantification of LC-MS data. BMC research notes. 2012;5 1:428.
41. Creek DJ, Jankevics A, Burgess KE, Breitling R and Barrett MP. IDEOM: an Excel interface for analysis of LC–MS-based metabolomics data. Bioinformatics. 2012;28 7:1048-9.
42. Conley CJ, Smith R, Torgrip RJ, Taylor RM, Tautenhahn R and Prince JT. Massifquant: open-source Kalman filter-based XC-MS isotope trace feature detection. Bioinformatics. 2014;30 18:2636-43.
43. Zhang W, Chang J, Lei Z, Huhman D, Sumner LW and Zhao PX. MET-COFEA: a liquid chromatography/mass spectrometry data processing platform for metabolite compound feature extraction and annotation. Analytical Chemistry. 2014;86 13:6245-53.
44. Zhang W, Lei Z, Huhman D, Sumner LW and Zhao PX. MET-XAlign: A metabolite cross-alignment tool for LC/MS-based comparative metabolomics. Analytical Chemistry. 2015;87 18:9114-9.
45. Yu T, Park Y, Johnson JM and Jones DP. apLCMS—adaptive processing of high-resolution LC/MS data. Bioinformatics. 2009;25 15:1930-6.
46. Uppal K, Soltow QA, Strobel FH, Pittard WS, Gernert KM, Yu T, et al. xMSanalyzer: automated pipeline for improved feature detection and downstream analysis of large-scale, non-targeted metabolomics data. BMC Bioinformatics. 2013;14 1:15.
47. Myint L, Kleensang A, Zhao L, Hartung T and Hansen KD. Joint bounding of peaks across samples improves differential analysis in mass spectrometry-based metabolomics. Analytical Chemistry. 2017; doi:10.1021/acs.analchem.6b04719.
48. Wandy J, Daly R, Breitling R and Rogers S. Incorporating peak grouping information for alignment of multiple liquid chromatography-mass spectrometry datasets. Bioinformatics. 2015;31 12:1999-2006.
49. Wehrens R, Bloemberg TG and Eilers PH. Fast parametric time warping of peak lists. Bioinformatics. 2015;31 18:3063-5.
50. Stein SE. An integrated method for spectrum extraction and compound identification from gas chromatography/mass spectrometry data. Journal of the American Society for Mass Spectrometry. 1999;10 8:770-81. doi:http://dx.doi.org/10.1016/S1044-0305(99)00047-1.
51. Aggio R, Villas SG and Ruggiero K. Metab: an R package for high-throughput analysis of metabolomics data generated by GC-MS. Bioinformatics. 2011;27 16:2316-8.

52. Bunk B, Kucklick M, Jonas R, Münch R, Schobert M, Jahn D, et al. MetaQuant: a tool for the automatic quantification of GC/MS-based metabolome data. Bioinformatics. 2006;22 23:2962-5.

53. Hiller K, Hangebrauk J, Jäger C, Spura J, Schreiber K and Schomburg D. MetaboliteDetector: comprehensive analysis tool for targeted and nontargeted GC/MS based metabolome analysis. Analytical Chemistry. 2009;81 9:3429-39.

54. Luedemann A, Strassburg K, Erban A and Kopka J. TagFinder for the quantitative analysis of gas chromatography—mass spectrometry (GC-MS)-based metabolite profiling experiments. Bioinformatics. 2008;24 5:732-7.

55. Cuadros-Inostroza Á, Caldana C, Redestig H, Kusano M, Lisec J, Peña-Cortés H, et al. TargetSearch-a Bioconductor package for the efficient preprocessing of GC-MS metabolite profiling data. BMC Bioinformatics. 2009;10 1:428.

56. O'Callaghan S, De Souza DP, Isaac A, Wang Q, Hodkinson L, Olshansky M, et al. PyMS: a Python toolkit for processing of gas chromatography-mass spectrometry (GC-MS) data. Application and comparative study of selected tools. BMC Bioinformatics. 2012;13 1:115.

57. Jellema RH, Krishnan S, Hendriks MM, Muilwijk B and Vogels JT. Deconvolution using signal segmentation. Chemometrics and Intelligent Laboratory Systems. 2010;104 1:132-9.

58. Wehrens R, Weingart G and Mattivi F. metaMS: An open-source pipeline for GC–MS-based untargeted metabolomics. Journal of Chromatography B. 2014;966:109-16.

59. Kuich PHJ, Hoffmann N and Kempa S. Maui-VIA: a user-friendly software for visual identification, alignment, correction, and quantification of gas chromatography–mass spectrometry data. Frontiers in bioengineering and biotechnology. 2014;2.

60. Domingo-Almenara X, Brezmes J, Vinaixa M, Samino S, Ramirez N, Ramon-Krauel M, et al. eRah: A Computational Tool Integrating Spectral Deconvolution and Alignment with Quantification and Identification of Metabolites in GC/MS-Based Metabolomics. Analytical Chemistry. 2016;88 19:9821-9.

61. Ni Y, Su M, Qiu Y, Jia W and Du X. ADAP-GC 3.0: Improved Peak Detection and Deconvolution of Co-eluting Metabolites from GC/TOF-MS Data for Metabolomics Studies. Analytical Chemistry. 2016;88 17:8802-11.

62. Wei X, Shi X, Koo I, Kim S, Schmidt RH, Arteel GE, et al. MetPP: a computational platform for comprehensive two-dimensional gas chromatography time-of-flight mass spectrometry-based metabolomics. Bioinformatics. 2013;29 14:1786-92. doi:10.1093/bioinformatics/btt275.

63. Kuhl C, Tautenhahn R, Böttcher C, Larson TR and Neumann S. CAMERA: An Integrated Strategy for Compound Spectra Extraction and Annotation of Liquid Chromatography/Mass Spectrometry Data Sets. Analytical Chemistry. 2012;84 1:283-9. doi:10.1021/ac202450g.

64. Alonso A, Julià A, Beltran A, Vinaixa M, Díaz M, Ibañez L, et al. AStream: an R package for annotating LC/MS metabolomic data. Bioinformatics. 2011;27 9:1339-40.

65. Kessler N, Walter F, Persicke M, Albaum SP, Kalinowski J, Goesmann A, et al. Allocator: An interactive web platform for the analysis of metabolomic LC-ESI-MS datasets, enabling semi-automated, user-revised compound annotation and mass isotopomer ratio analysis. PLoS One. 2014;9 11:e113909.

66. Tikunov Y, Laptenok S, Hall R, Bovy A and De Vos R. MSClust: a tool for unsupervised mass spectra extraction of chromatography-mass spectrometry ion-wise aligned data. Metabolomics. 2012;8 4:714-8.

67. Broeckling CD, Afsar F, Neumann S, Ben-Hur A and Prenni J. RAMClust: a novel feature clustering method enables spectral-matching-based annotation for metabolomics data. Analytical Chemistry. 2014;86 14:6812-7.

68. Gu H, Gowda GN, Neto FC, Opp MR and Raftery D. RAMSY: ratio analysis of mass spectrometry to improve compound identification. Analytical Chemistry. 2013;85 22:10771-9.

69. Chen G, Cui L, Teo GS, Ong CN, Tan CS and Choi H. MetTailor: dynamic block summary and intensity normalization for robust analysis of mass spectrometry data in metabolomics. Bioinformatics. 2015:btv434.

70. Chawade A, Alexandersson E and Levander F. Normalyzer: a tool for rapid evaluation of normalization methods for omics data sets. Journal of Proteome Research. 2014;13 6:3114-20.

71. Fernández-Albert F, Llorach R, Garcia-Aloy M, Ziyatdinov A, Andres-Lacueva C and Perera A. Intensity drift removal in LC/MS metabolomics by common variance compensation. Bioinformatics. 2014;30 20:2899-905.

72. Shen X, Gong X, Cai Y, Guo Y, Tu J, Li H, et al. Normalization and integration of large-scale metabolomics data using support vector regression. Metabolomics. 2016;12 5:1-12.

73. Karpievitch YV, Nikolic SB, Wilson R, Sharman JE and Edwards LM. Metabolomics Data Normalization with EigenMS. PLoS One. 2015;9 12:e116221. doi:10.1371/journal.pone.0116221.

74. Huege J, Goetze J, Dethloff F, Junker B and Kopka J. Quantification of stable isotope label in metabolites via mass spectrometry. Plant Chemical Genomics: Methods and Protocols. 2014:213-23.

75. Millard P, Letisse F, Sokol S and Portais J-C. IsoCor: correcting MS data in isotope labeling experiments. Bioinformatics. 2012;28 9:1294-6.

76. Jungreuthmayer C, Neubauer S, Mairinger T, Zanghellini J and Hann S. ICT: isotope correction toolbox. Bioinformatics. 2016;32 1:154-6.

77. Chokkathukalam A, Jankevics A, Creek DJ, Achcar F, Barrett MP and Breitling R. mzMatch–ISO: an R tool for the annotation and relative quantification of isotope-labelled mass spectrometry data. Bioinformatics. 2013;29 2:281-3.

78. Bueschl C, Kluger B, Berthiller F, Lirk G, Winkler S, Krska R, et al. MetExtract: a new software tool for the automated comprehensive extraction of metabolite-derived LC/MS signals in metabolomics research. Bioinformatics. 2012;28 5:736-8.

79. Huang X, Chen Y-J, Cho K, Nikolskiy I, Crawford PA and Patti GJ. X13CMS: global tracking of isotopic labels in untargeted metabolomics. Analytical Chemistry. 2014;86 3:1632-9.

80. Capellades J, Navarro M, Samino S, Garcia-Ramirez M, Hernandez C, Simo R, et al. geoRge: A computational tool to detect the presence of stable isotope labeling in LC/MS-based untargeted metabolomics. Analytical Chemistry. 2015;88 1:621-8.

81. Weindl D, Wegner A and Hiller K. MIA: non-targeted mass isotopolome analysis. Bioinformatics. 2016:btw317.

82. Cai Y, Weng K, Guo Y, Peng J and Zhu Z-J. An integrated targeted metabolomic platform for high-throughput metabolite profiling and automated data processing. Metabolomics. 2015;11 6:1575-86.

83. Wong JW, Abuhusain HJ, McDonald KL and Don AS. MMSAT: automated quantification of metabolites in selected reaction monitoring experiments. Analytical Chemistry. 2011;84 1:470-4.

84. Tsugawa H, Arita M, Kanazawa M, Ogiwara A, Bamba T and Fukusaki E. MRMPROBS: A data assessment and metabolite identification tool for large-scale multiple reaction monitoring based widely targeted metabolomics. Analytical Chemistry. 2013;85 10:5191-9.

85. Nikolskiy I, Mahieu NG, Chen Y-J, Tautenhahn R and Patti GJ. An untargeted metabolomic workflow to improve structural characterization of metabolites. Analytical Chemistry. 2013;85 16:7713-9.

86. Ma Y, Kind T, Yang D, Leon C and Fiehn O. MS2Analyzer: A software for small molecule substructure annotations from accurate tandem mass spectra. Analytical Chemistry. 2014;86 21:10724-31.

87. van der Hooft JJJ, Wandy J, Barrett MP, Burgess KEV and Rogers S. Topic modeling for untargeted substructure exploration in metabolomics. Proceedings of the National Academy of Sciences. 2016;113 48:13738-43. doi:10.1073/pnas.1608041113.

88. Tsugawa H, Cajka T, Kind T, Ma Y, Higgins B, Ikeda K, et al. MS-DIAL: data-independent MS/MS deconvolution for comprehensive metabolome analysis. Nature methods. 2015;12 6:523-6.

89. Li H, Cai Y, Guo Y, Chen F and Zhu Z-J. MetDIA: Targeted Metabolite Extraction of Multiplexed MS/MS Spectra Generated by Data-Independent Acquisition. Analytical Chemistry. 2016;88 17:8757-64.

90. Libiseller G, Dvorzak M, Kleb U, Gander E, Eisenberg T, Madeo F, et al. IPO: a tool for automated optimization of XCMS parameters. BMC Bioinformatics. 2015;16 1:118.

91. Mahieu NG, Huang X, Chen Y-J and Patti GJ. Credentialing features: a platform to benchmark and optimize untargeted metabolomic methods. Analytical Chemistry. 2014;86 19:9583-9.

92. Brodsky L, Moussaieff A, Shahaf N, Aharoni A and Rogachev I. Evaluation of Peak Picking Quality in LC− MS Metabolomics Data. Analytical Chemistry. 2010;82 22:9177-87.

93. Ranjbar MRN, Di Poto C, Wang Y and Ressom HW. Simat: Gc-sim-ms data analysis tool. BMC Bioinformatics. 2015;16 1:259.

94. Mak TD, Laiakis EC, Goudarzi M and Fornace Jr AJ. Metabolyzer: A novel statistical workflow for analyzing postprocessed lc–ms metabolomics data. Analytical Chemistry. 2013;86 1:506-13.

95. Kastenmüller G, Römisch-Margl W, Wägele B, Altmaier E and Suhre K. metaP-server: a web-based metabolomics data analysis tool. BioMed Research International. 2010;2011.

96. Fitzpatrick MA, McGrath CM and Young SP. Pathomx: an interactive workflow-based tool for the analysis of metabolomic data. BMC Bioinformatics. 2014;15 1:396.

97. Hughes G, Cruickshank-Quinn C, Reisdorph R, Lutz S, Petrache I, Reisdorph N, et al. MSPrep—Summarization, normalization and diagnostics for processing of mass spectrometry–based metabolomic data. Bioinformatics. 2014;30 1:133-4.

98. Sun X and Weckwerth W. COVAIN: a toolbox for uni-and multivariate statistics, time-series and correlation network analysis and inverse estimation of the differential Jacobian from metabolomics covariance data. Metabolomics. 2012;8 1:81-93.

99. Glaab E and Schneider R. RepExplore: Addressing technical replicate variance in proteomics and metabolomics data analysis. Bioinformatics. 2015:btv127.

100. Zhan X, Patterson AD and Ghosh D. Kernel approaches for differential expression analysis of mass spectrometry-based metabolomics data. BMC Bioinformatics. 2015;16 1:77.

101. Nodzenski M, Muehlbauer MJ, Bain JR, Reisetter AC, Lowe WL and Scholtens DM. Metabomxtr: an R package for mixture-model analysis of non-targeted metabolomics data. Bioinformatics. 2014;30 22:3287-8.

102. Suvitaival T, Rogers S and Kaski S. Stronger findings from mass spectral data through multi-peak modeling. BMC Bioinformatics. 2014;15 1:208.

103. Mak TD, Laiakis EC, Goudarzi M and Fornace Jr AJ. Selective paired ion contrast analysis: a novel algorithm for analyzing postprocessed LC-MS metabolomics data possessing high experimental noise. Analytical Chemistry. 2015;87 6:3177-86.

104. Ernest B, Gooding JR, Campagna SR, Saxton AM and Voy BH. MetabR: an R script for linear model analysis of quantitative metabolomic data. BMC research notes. 2012;5 1:596.

105. Huang J-H, Yan J, Wu Q-H, Ferro MD, Yi L-Z, Lu H-M, et al. Selective of informative metabolites using random forests based on model population analysis. Talanta. 2013;117:549-55.

106. Simader AM, Kluger B, Neumann NKN, Bueschl C, Lemmens M, Lirk G, et al. QCScreen: a software tool for data quality control in LC-HRMS based metabolomics. BMC Bioinformatics. 2015;16 1:341.

107. Styczynski MP, Moxley JF, Tong LV, Walther JL, Jensen KL and Stephanopoulos GN. Systematic identification of conserved metabolites in GC/MS data for metabolomics and biomarker discovery. Analytical Chemistry. 2007;79 3:966-73.

108. Baran R, Kochi H, Saito N, Suematsu M, Soga T, Nishioka T, et al. MathDAMP: a package for differential analysis of metabolite profiles. BMC Bioinformatics. 2006;7 1:530.

109. Fernie AR. The future of metabolic phytochemistry: Larger numbers of metabolites, higher resolution, greater understanding. Phytochemistry. 2007;68 22–24:2861-80. doi:http://dx.doi.org/10.1016/j.phytochem.2007.07.010.

110. Tohge T, Wendenburg R, Ishihara H, Nakabayashi R, Watanabe M, Sulpice R, et al. Characterization of a recently evolved flavonol-phenylacyltransferase gene provides signatures of natural light selection in Brassicaceae. Nature communications. 2016;7.

111. Zhou B, Wang J and Ressom HW. MetaboSearch: tool for mass-based metabolite identification using multiple databases. PLoS One. 2012;7 6:e40096.

112. Brown M, Wedge DC, Goodacre R, Kell DB, Baker PN, Kenny LC, et al. Automated workflows for accurate mass-based putative metabolite identification in LC/MS-derived metabolomic datasets. Bioinformatics. 2011;27 8:1108-12.

113. Daly R, Rogers S, Wandy J, Jankevics A, Burgess KE and Breitling R. MetAssign: probabilistic annotation of metabolites from LC–MS data using a Bayesian clustering approach. Bioinformatics. 2014;30 19:2764-71.

114. Böcker S, Letzel MC, Lipták Z and Pervukhin A. SIRIUS: decomposing isotope patterns for metabolite identification. Bioinformatics. 2009;25 2:218-24.

115. Sakurai N, Ara T, Kanaya S, Nakamura Y, Iijima Y, Enomoto M, et al. An application of a relational database system for high-throughput prediction of elemental compositions from accurate mass values. Bioinformatics. 2013;29 2:290-1.

116. Lommen A. Ultrafast PubChem searching combined with improved filtering rules for elemental composition analysis. Analytical Chemistry. 2014;86 11:5463-9.

117. Dhanasekaran AR, Pearson JL, Ganesan B and Weimer BC. Metabolome searcher: a high throughput tool for metabolite identification and metabolic pathway mapping directly from mass spectrometry and using genome restriction. BMC Bioinformatics. 2015;16 1:62.

118. Suhre K and Schmitt-Kopplin P. MassTRIX: mass translator into pathways. Nucleic acids research. 2008;36 suppl 2:W481-W4.

119. Uppal K, Soltow QA, Promislow DE, Wachtman LM, Quyyumi AA and Jones DP. MetabNet: an R package for metabolic association analysis of high-resolution metabolomics data. Frontiers in bioengineering and biotechnology. 2015;3:87.

120. Silva RR, Jourdan F, Salvanha DM, Letisse F, Jamin EL, Guidetti-Gonzalez S, et al. ProbMetab: an R package for Bayesian probabilistic annotation of LC–MS-based metabolomics. Bioinformatics. 2014;30 9:1336-7.

121. Rogers S, Scheltema RA, Girolami M and Breitling R. Probabilistic assignment of formulas to mass peaks in metabolomics experiments. Bioinformatics. 2009;25 4:512-8. doi:10.1093/bioinformatics/btn642.

122. Weber RJ and Viant MR. MI-Pack: Increased confidence of metabolite identification in mass spectra by integrating accurate masses and metabolic pathways. Chemometrics and Intelligent Laboratory Systems. 2010;104 1:75-82.

123. Qiu F, Fine DD, Wherritt DJ, Lei Z and Sumner LW. PlantMAT: A Metabolomics Tool for Predicting the Specialized Metabolic Potential of a System and for Large-Scale Metabolite Identifications. Analytical Chemistry. 2016;88 23:11373-83.

124. Ruttkies C, Schymanski EL, Wolf S, Hollender J and Neumann S. MetFrag relaunched: incorporating strategies beyond in silico fragmentation. Journal of cheminformatics. 2016;8 1:3.

125. Menikarachchi LC, Cawley S, Hill DW, Hall LM, Hall L, Lai S, et al. MolFind: a software package enabling HPLC/MS-based identification of unknown chemical structures. Analytical Chemistry. 2012;84 21:9388-94.
126. Allen F, Pon A, Wilson M, Greiner R and Wishart D. CFM-ID: a web server for annotation, spectrum prediction and metabolite identification from tandem mass spectra. Nucleic acids research. 2014;42 W1:W94-W9.
127. Ridder L, van der Hooft JJ and Verhoeven S. Automatic compound annotation from mass spectrometry data using MAGMa. Mass Spectrometry. 2014;3 Special_Issue_2:S0033-S.
128. Heinonen M, Shen H, Zamboni N and Rousu J. Metabolite identification and molecular fingerprint prediction through machine learning. Bioinformatics. 2012;28 18:2333-41.
129. Dührkop K, Shen H, Meusel M, Rousu J and Böcker S. Searching molecular structure databases with tandem mass spectra using CSI: FingerID. Proceedings of the National Academy of Sciences. 2015;112 41:12580-5.
130. Gerlich M and Neumann S. MetFusion: integration of compound identification strategies. Journal of Mass Spectrometry. 2013;48 3:291-8.
131. Leader DP, Burgess K, Creek D and Barrett MP. Pathos: A web facility that uses metabolic maps to display experimental changes in metabolites identified by mass spectrometry. Rapid Communications in Mass Spectrometry. 2011;25 22:3422-6.
132. Pon A, Jewison T, Su Y, Liang Y, Knox C, Maciejewski A, et al. Pathways with PathWhiz. Nucleic acids research. 2015:gkv399.
133. Yamada T, Letunic I, Okuda S, Kanehisa M and Bork P. iPath2. 0: interactive pathway explorer. Nucleic acids research. 2011;39 suppl 2:W412-W5.
134. Kutmon M, van Iersel MP, Bohler A, Kelder T, Nunes N, Pico AR, et al. PathVisio 3: an extendable pathway analysis toolbox. PLoS Comput Biol. 2015;11 2:e1004085.
135. Pathan M, Keerthikumar S, Ang CS, Gangoda L, Quek CY, Williamson NA, et al. FunRich: An open access standalone functional enrichment and interaction network analysis tool. Proteomics. 2015;15 15:2597-601.
136. Moreno P, Beisken S, Harsha B, Muthukrishnan V, Tudose I, Dekker A, et al. BiNChE: a web tool and library for chemical enrichment analysis based on the ChEBI ontology. BMC Bioinformatics. 2015;16 1:56.
137. Kankainen M, Gopalacharyulu P, Holm L and Orešič M. MPEA—metabolite pathway enrichment analysis. Bioinformatics. 2011;27 13:1878-9.
138. Aggio RB, Ruggiero K and Villas-Bôas SG. Pathway Activity Profiling (PAPi): from the metabolite profile to the metabolic pathway activity. Bioinformatics. 2010;26 23:2969-76.
139. Eichner J, Rosenbaum L, Wrzodek C, Häring H-U, Zell A and Lehmann R. Integrated enrichment analysis and pathway-centered visualization of metabolomics, proteomics, transcriptomics, and genomics data by using the InCroMAP software. Journal of Chromatography B. 2014;966:77-82.
140. Carazzolle MF, de Carvalho LM, Slepicka HH, Vidal RO, Pereira GAG, Kobarg J, et al. IIS–Integrated Interactome System: a web-based platform for the annotation, analysis and visualization of protein-metabolite-gene-drug interactions by integrating a variety of data sources and tools. PLoS One. 2014;9 6:e100385.
141. Sakurai N, Ara T, Ogata Y, Sano R, Ohno T, Sugiyama K, et al. KaPPA-View4: a metabolic pathway database for representation and analysis of correlation networks of gene co-expression and metabolite co-accumulation and omics data. Nucleic acids research. 2011;39 suppl 1:D677-D84.
142. Usadel B, Poree F, Nagel A, Lohse M, CZEDIK-EYSENBERG A and Stitt M. A guide to using MapMan to visualize and compare Omics data in plants: a case study in the crop species, Maize. Plant, cell & environment. 2009;32 9:1211-29.
143. Neuweger H, Persicke M, Albaum SP, Bekel T, Dondrup M, Hüser AT, et al. Visualizing post genomics data-sets on customized pathway maps by ProMeTra–aeration-dependent gene

expression and metabolism of Corynebacterium glutamicum as an example. BMC systems biology. 2009;3 1:82.

144. García-Alcalde F, García-López F, Dopazo J and Conesa A. Paintomics: a web based tool for the joint visualization of transcriptomics and metabolomics data. Bioinformatics. 2011;27 1:137-9.

145. Rohn H, Junker A, Hartmann A, Grafahrend-Belau E, Treutler H, Klapperstück M, et al. VANTED v2: a framework for systems biology applications. BMC systems biology. 2012;6 1:139.

146. López-Ibáñez J, Pazos F and Chagoyen M. MBROLE 2.0—functional enrichment of chemical compounds. Nucleic acids research. 2016;44 W1:W201-W4.

147. Kamburov A, Cavill R, Ebbels TM, Herwig R and Keun HC. Integrated pathway-level analysis of transcriptomics and metabolomics data with IMPaLA. Bioinformatics. 2011;27 20:2917-8.

148. Jourdan F, Breitling R, Barrett MP and Gilbert D. MetaNetter: inference and visualization of high-resolution metabolomic networks. Bioinformatics. 2008;24 1:143-5.

149. Grapov D, Wanichthanarak K and Fiehn O. MetaMapR: pathway independent metabolomic network analysis incorporating unknowns. Bioinformatics. 2015:btv194.

150. Lu J and Carlson HA. ChemTreeMap: an interactive map of biochemical similarity in molecular datasets. Bioinformatics. 2016;32 23:3584-92. doi:10.1093/bioinformatics/btw523.

151. Treutler H, Tsugawa H, Porzel A, Gorzolka K, Tissier A, Neumann S, et al. Discovering Regulated Metabolite Families in Untargeted Metabolomics Studies. Analytical Chemistry. 2016;88 16:8082-90.

152. Hamdalla MA, Rajasekaran S, Grant DF and Măndoiu II. Metabolic Pathway Predictions for Metabolomics: A Molecular Structure Matching Approach. Journal of chemical information and modeling. 2015;55 3:709-18.

153. Pence HE and Williams A. ChemSpider: an online chemical information resource. ACS Publications, 2010.

154. Kim S, Thiessen PA, Bolton EE, Chen J, Fu G, Gindulyte A, et al. PubChem substance and compound databases. Nucleic acids research. 2015:gkv951.

155. Hastings J, Owen G, Dekker A, Ennis M, Kale N, Muthukrishnan V, et al. ChEBI in 2016: Improved services and an expanding collection of metabolites. Nucleic acids research. 2015:gkv1031.

156. Gaulton A, Bellis LJ, Bento AP, Chambers J, Davies M, Hersey A, et al. ChEMBL: a large-scale bioactivity database for drug discovery. Nucleic acids research. 2012;40 D1:D1100-D7.

157. Seiler KP, George GA, Happ MP, Bodycombe NE, Carrinski HA, Norton S, et al. ChemBank: a small-molecule screening and cheminformatics resource database. Nucleic acids research. 2008;36 suppl 1:D351-D9.

158. Wishart DS, Jewison T, Guo AC, Wilson M, Knox C, Liu Y, et al. HMDB 3.0—the human metabolome database in 2013. Nucleic acids research. 2012:gks1065.

159. Cui Q, Lewis IA, Hegeman AD, Anderson ME, Li J, Schulte CF, et al. Metabolite identification via the Madison Metabolomics Consortium Database. Nat Biotech. 2008;26 2:162-4. doi:http://www.nature.com/nbt/journal/v26/n2/suppinfo/nbt0208-162_S1.html.

160. Masciocchi J, Frau G, Fanton M, Sturlese M, Floris M, Pireddu L, et al. MMsINC: a large-scale chemoinformatics database. Nucleic acids research. 2009;37 suppl 1:D284-D90.

161. Afendi FM, Okada T, Yamazaki M, Hirai-Morita A, Nakamura Y, Nakamura K, et al. KNApSAcK family databases: integrated metabolite–plant species databases for multifaceted plant research. Plant and Cell Physiology. 2012;53 2:e1-e.

162. Arita M and Suwa K. Search extension transforms Wiki into a relational system: a case for flavonoid metabolite database. BioData mining. 2008;1 1:7.

163. Sharma A, Dutta P, Sharma M, Rajput NK, Dodiya B, Georrge JJ, et al. BioPhytMol: a drug discovery community resource on anti-mycobacterial phytomolecules and plant extracts. Journal of cheminformatics. 2014;6 1:46.

164. Kumari S, Pundhir S, Priya P, Jeena G, Punetha A, Chawla K, et al. EssOilDB: a database of essential oils reflecting terpene composition and variability in the plant kingdom. Database. 2014;2014:bau120.

165. Hummel J, Selbig J, Walther D and Kopka J. The Golm Metabolome Database: a database for GC-MS based metabolite profiling. Metabolomics. Springer; 2007. p. 75-95.

166. Skogerson K, Wohlgemuth G, Barupal DK and Fiehn O. The volatile compound BinBase mass spectral database. BMC Bioinformatics. 2011;12 1:321.

167. Horai H, Arita M, Kanaya S, Nihei Y, Ikeda T, Suwa K, et al. MassBank: a public repository for sharing mass spectral data for life sciences. Journal of Mass Spectrometry. 2010;45 7:703-14.

168. Smith CA, O'Maille G, Want EJ, Qin C, Trauger SA, Brandon TR, et al. METLIN: a metabolite mass spectral database. Therapeutic drug monitoring. 2005;27 6:747-51.

169. Cho K, Mahieu N, Ivanisevic J, Uritboonthai W, Chen Y-J, Siuzdak G, et al. isoMETLIN: a database for isotope-based metabolomics. Analytical Chemistry. 2014;86 19:9358-61.

170. Wishart D, Arndt D, Pon A, Sajed T, Guo AC, Djoumbou Y, et al. T3DB: the toxic exposome database. Nucleic acids research. 2015;43 D1:D928-D34.

171. Cuthbertson DJ, Johnson SR, Piljac-Žegarac J, Kappel J, Schäfer S, Wüst M, et al. Accurate mass–time tag library for LC/MS-based metabolite profiling of medicinal plants. Phytochemistry. 2013;91:187-97.

172. Sawada Y, Nakabayashi R, Yamada Y, Suzuki M, Sato M, Sakata A, et al. RIKEN tandem mass spectral database (ReSpect) for phytochemicals: a plant-specific MS/MS-based data resource and database. Phytochemistry. 2012;82:38-45.

173. Shahaf N, Rogachev I, Heinig U, Meir S, Malitsky S, Battat M, et al. The WEIZMASS spectral library for high-confidence metabolite identification. Nature communications. 2016;7.

174. Weber RJM, Li E, Bruty J, He S and Viant MR. MaConDa: a publicly accessible mass spectrometry contaminants database. Bioinformatics. 2012;28 21:2856-7. doi:10.1093/bioinformatics/bts527.

175. Matsuda F, Hirai MY, Sasaki E, Akiyama K, Yonekura-Sakakibara K, Provart NJ, et al. AtMetExpress development: a phytochemical atlas of Arabidopsis development. Plant Physiology. 2010;152 2:566-78.

176. Fukushima A, Kusano M, Mejia RF, Iwasa M, Kobayashi M, Hayashi N, et al. Metabolomic characterization of knockout mutants in Arabidopsis: development of a metabolite profiling database for knockout mutants in Arabidopsis. Plant Physiology. 2014;165 3:948-61.

177. Moco S, Bino RJ, Vorst O, Verhoeven HA, de Groot J, van Beek TA, et al. A liquid chromatography-mass spectrometry-based metabolome database for tomato. Plant Physiology. 2006;141 4:1205-18.

178. Brockmöller T, Ling Z, Li D, Gaquerel E, Baldwin IT and Xu S. Nicotiana attenuata Data Hub (Na DH): an integrative platform for exploring genomic, transcriptomic and metabolomic data in wild tobacco. BMC genomics. 2017;18 1:79.

179. Colmsee C, Mascher M, Czauderna T, Hartmann A, Schlüter U, Zellerhoff N, et al. OPTIMAS-DW: a comprehensive transcriptomics, metabolomics, ionomics, proteomics and phenomics data resource for maize. BMC plant biology. 2012;12 1:245.

180. Joshi T, Yao Q, Levi DF, Brechenmacher L, Valliyodan B, Stacey G, et al. SoyMetDB: the soybean metabolome database. In: *Bioinformatics and Biomedicine (BIBM), 2010 IEEE International Conference on* 2010, pp.203-8. IEEE.

181. Iijima Y, Nakamura Y, Ogata Y, Tanaka Ki, Sakurai N, Suda K, et al. Metabolite annotations based on the integration of mass spectral information. The Plant Journal. 2008;54 5:949-62.

182. Matsuda F, Yonekura-Sakakibara K, Niida R, Kuromori T, Shinozaki K and Saito K. MS/MS spectral tag-based annotation of non-targeted profile of plant secondary metabolites. The Plant Journal. 2009;57 3:555-77.

183. Hur M, Campbell AA, Almeida-de-Macedo M, Li L, Ransom N, Jose A, et al. A global approach to analysis and interpretation of metabolic data for plant natural product discovery. Natural product reports. 2013;30 4:565-83.

184. Wurtele ES, Chappell J, Jones AD, Celiz MD, Ransom N, Hur M, et al. Medicinal plants: a public resource for metabolomics and hypothesis development. Metabolites. 2012;2 4:1031-59.

185. Sud M, Fahy E, Cotter D, Azam K, Vadivelu I, Burant C, et al. Metabolomics Workbench: An international repository for metabolomics data and metadata, metabolite standards, protocols, tutorials and training, and analysis tools. Nucleic acids research. 2015:gkv1042.

186. Haug K, Salek RM, Conesa P, Hastings J, de Matos P, Rijnbeek M, et al. MetaboLights—an open-access general-purpose repository for metabolomics studies and associated meta-data. Nucleic acids research. 2012:gks1004.

187. Cook CE, Bergman MT, Finn RD, Cochrane G, Birney E and Apweiler R. The European Bioinformatics Institute in 2016: data growth and integration. Nucleic acids research. 2016;44 D1:D20-D6.

188. Wang M, Carver JJ, Phelan VV, Sanchez LM, Garg N, Peng Y, et al. Sharing and community curation of mass spectrometry data with Global Natural Products Social Molecular Networking. Nat Biotechnol. 2016;34 8:828-37.

189. Kanehisa M and Goto S. KEGG: kyoto encyclopedia of genes and genomes. Nucleic acids research. 2000;28 1:27-30.

190. Kelder T, Pico AR, Hanspers K, Van Iersel MP, Evelo C and Conklin BR. Mining biological pathways using WikiPathways web services. PLoS One. 2009;4 7:e6447.

191. Navas-Delgado I, García-Godoy MJ, López-Camacho E, Rybinski M, Reyes-Palomares A, Medina MÁ, et al. kpath: integration of metabolic pathway linked data. Database. 2015;2015:bav053.

192. Caspi R, Foerster H, Fulcher CA, Kaipa P, Krummenacker M, Latendresse M, et al. The MetaCyc Database of metabolic pathways and enzymes and the BioCyc collection of Pathway/Genome Databases. Nucleic acids research. 2008;36 suppl 1:D623-D31.

193. Arkin AP, Stevens RL, Cottingham RW, Maslov S, Henry CS, Dehal P, et al. The DOE Systems Biology Knowledgebase (KBase). bioRxiv. 2016:096354.

194. Schreiber F, Colmsee C, Czauderna T, Grafahrend-Belau E, Hartmann A, Junker A, et al. MetaCrop 2.0: managing and exploring information about crop plant metabolism. Nucleic acids research. 2011:gkr1004.

195. Sucaet Y, Wang Y, Li J and Wurtele ES. MetNet Online: a novel integrated resource for plant systems biology. BMC Bioinformatics. 2012;13 1:267.

196. Tello-Ruiz MK, Stein J, Wei S, Preece J, Olson A, Naithani S, et al. Gramene 2016: comparative plant genomics and pathway resources. Nucleic acids research. 2015:gkv1179.

197. Ott MA and Vriend G. Correcting ligands, metabolites, and pathways. BMC Bioinformatics. 2006;7 1:517.

198. Lang M, Stelzer M and Schomburg D. BKM-react, an integrated biochemical reaction database. BMC biochemistry. 2011;12 1:42.

199. Kumar A, Suthers PF and Maranas CD. MetRxn: a knowledgebase of metabolites and reactions spanning metabolic models and databases. BMC Bioinformatics. 2012;13 1:6.

200. Jeffryes JG, Colastani RL, Elbadawi-Sidhu M, Kind T, Niehaus TD, Broadbelt LJ, et al. MINEs: open access databases of computationally predicted enzyme promiscuity products for untargeted metabolomics. Journal of cheminformatics. 2015;7 1:44.

201. Zhou Z, Shen X, Tu J and Zhu Z-J. Large-Scale Prediction of Collision Cross-Section Values for Metabolites in Ion Mobility-Mass Spectrometry. Analytical Chemistry. 2016;88 22:11084-91.

202. Allard P-M, Péresse T, Bisson J, Gindro K, Marcourt L, Pham VC, et al. Integration of molecular networking and in-silico MS/MS fragmentation for natural products dereplication. Analytical Chemistry. 2016;88 6:3317-23.
203. Palla P, Frau G, Vargiu L and Rodriguez-Tomé P. QTREDS: a Ruby on Rails-based platform for omics laboratories. BMC Bioinformatics. 2014;15 1:S13.
204. Hunter A, Dayalan S, De Souza D, Power B, Lorrimar R, Szabo T, et al. MASTR-MS: a web-based collaborative laboratory information management system (LIMS) for metabolomics. Metabolomics. 2017;13 2:14.
205. Franceschi P, Mylonas R, Shahaf N, Scholz M, Arapitsas P, Masuero D, et al. MetaDB a data processing workflow in untargeted MS-based metabolomics experiments. Frontiers in bioengineering and biotechnology. 2014;2:72.
206. Ara T, Enomoto M, Arita M, Ikeda C, Kera K, Yamada M, et al. Metabolonote: a wiki-based database for managing hierarchical metadata of metabolome analyses. Frontiers in bioengineering and biotechnology. 2015;3:38.
207. Wohlgemuth G, Mehta SS, Mejia RF, Neumann S, Pedrosa D, Pluskal T, et al. SPLASH, a hashed identifier for mass spectra. Nat Biotechnol. 2016;34 11:1099-101.
208. Redestig H, Kusano M, Fukushima A, Matsuda F, Saito K and Arita M. Consolidating metabolite identifiers to enable contextual and multi-platform metabolomics data analysis. BMC Bioinformatics. 2010;11 1:214.
209. Wohlgemuth G, Haldiya PK, Willighagen E, Kind T and Fiehn O. The Chemical Translation Service—a web-based tool to improve standardization of metabolomic reports. Bioinformatics. 2010;26 20:2647-8.
210. Carroll AJ, Zhang P, Whitehead L, Kaines S, Tcherkez G and Badger MR. PhenoMeter: a metabolome database search tool using statistical similarity matching of metabolic phenotypes for high-confidence detection of functional links. Frontiers in bioengineering and biotechnology. 2015;3.
211. Sartor MA, Ade A, Wright Z, Omenn GS, Athey B and Karnovsky A. Metab2MeSH: annotating compounds with medical subject headings. Bioinformatics. 2012;28 10:1408-10.
212. Xu QW, Griss J, Wang R, Jones AR, Hermjakob H and Vizcaíno JA. jmzTab: A Java interface to the mzTab data standard. Proteomics. 2014;14 11:1328-32.
213. Scheltema RA, Jankevics A, Jansen RC, Swertz MA and Breitling R. PeakML/mzMatch: a file format, Java library, R library, and tool-chain for mass spectrometry data analysis. Analytical Chemistry. 2011;83 7:2786-93.
214. Avtonomov DM, Raskind A and Nesvizhskii AI. BatMass: a Java Software Platform for LC–MS Data Visualization in Proteomics and Metabolomics. Journal of Proteome Research. 2016;15 8:2500-9.
215. Tanaka S, Fujita Y, Parry HE, Yoshizawa AC, Morimoto K, Murase M, et al. Mass++: A visualization and analysis tool for mass spectrometry. Journal of Proteome Research. 2014;13 8:3846-53.
216. Beisken S, Conesa P, Haug K, Salek RM and Steinbeck C. SpeckTackle: JavaScript charts for spectroscopy. Journal of cheminformatics. 2015;7 1:17.
217. Stravs MA, Schymanski EL, Singer HP and Hollender J. Automatic recalibration and processing of tandem mass spectra using formula annotation. Journal of Mass Spectrometry. 2013;48 1:89-99.
218. Dong Y, Li B and Aharoni A. More than Pictures: When MS Imaging Meets Histology. Trends in plant science. 2016;21 8:686-98.
219. Wijetunge CD, Saeed I, Boughton BA, Spraggins JM, Caprioli RM, Bacic A, et al. EXIMS: an improved data analysis pipeline based on a new peak picking method for EXploring Imaging Mass Spectrometry data. Bioinformatics. 2015;31 19:3198-206.

220. Rübel O, Greiner A, Cholia S, Louie K, Bethel EW, Northen TR, et al. OpenMSI: a high-performance web-based platform for mass spectrometry imaging. Analytical Chemistry. 2013;85 21:10354-61.

221. Husen P, Tarasov K, Katafiasz M, Sokol E, Vogt J, Baumgart J, et al. Analysis of lipid experiments (ALEX): a software framework for analysis of high-resolution shotgun lipidomics data. PLoS One. 2013;8 11:e79736.

222. Tsugawa H, Ohta E, Izumi Y, Ogiwara A, Yukihira D, Bamba T, et al. MRM-DIFF: data processing strategy for differential analysis in large scale MRM-based lipidomics studies. Frontiers in genetics. 2014;5.

223. Wong G, Chan J, Kingwell BA, Leckie C and Meikle PJ. LICRE: unsupervised feature correlation reduction for lipidomics. Bioinformatics. 2014:btu381.

224. Herzog R, Schuhmann K, Schwudke D, Sampaio JL, Bornstein SR, Schroeder M, et al. LipidXplorer: a software for consensual cross-platform lipidomics. PLoS One. 2012;7 1:e29851.

225. Haimi P, Uphoff A, Hermansson M and Somerharju P. Software tools for analysis of mass spectrometric lipidome data. Analytical Chemistry. 2006;78 24:8324-31.

226. Blanchard AP, McDowell GS, Valenzuela N, Xu H, Gelbard S, Bertrand M, et al. Visualization and Phospholipid Identification (VaLID): online integrated search engine capable of identifying and visualizing glycerophospholipids with given mass. Bioinformatics. 2013;29 2:284-5.

227. Collins JR, Edwards BR, Fredricks HF and Van Mooy BA. LOBSTAHS: an adduct-based lipidomics strategy for discovery and identification of oxidative stress biomarkers. Analytical Chemistry. 2016;88 14:7154-62.

228. Ahmed Z, Mayr M, Zeeshan S, Dandekar T, Mueller MJ and Fekete A. Lipid-Pro: a computational lipid identification solution for untargeted lipidomics on data-independent acquisition tandem mass spectrometry platforms. Bioinformatics. 2015;31 7:1150-3.

229. Hartler J, Trötzmüller M, Chitraju C, Spener F, Köfeler HC and Thallinger GG. Lipid Data Analyzer: unattended identification and quantitation of lipids in LC-MS data. Bioinformatics. 2011;27 4:572-7.

230. Song H, Hsu F-F, Ladenson J and Turk J. Algorithm for processing raw mass spectrometric data to identify and quantitate complex lipid molecular species in mixtures by data-dependent scanning and fragment ion database searching. Journal of the American Society for Mass Spectrometry. 2007;18 10:1848-58.

231. Sud M, Fahy E, Cotter D, Brown A, Dennis EA, Glass CK, et al. Lmsd: lipid maps structure database. Nucleic acids research. 2007;35 suppl 1:D527-D32.

232. Watanabe K, Yasugi E and Oshima M. How to search the glycolipid data in “LIPIDBANK for Web”, the newly developed lipid database in Japan. Trends in Glycoscience and Glycotechnology. 2000;12 65:175-84.

233. Kind T, Liu K-H, Lee DY, DeFelice B, Meissen JK and Fiehn O. LipidBlast in silico tandem mass spectrometry database for lipid identification. Nature methods. 2013;10 8:755-8.

234. Foster JM, Moreno P, Fabregat A, Hermjakob H, Steinbeck C, Apweiler R, et al. LipidHome: a database of theoretical lipids optimized for high throughput mass spectrometry lipidomics. PLoS One. 2013;8 5:e61951.

235. Aimo L, Liechti R, Nouspikel N, Niknejad A, Gleizes A, Götz L, et al. The SwissLipids knowledgebase for lipid biology. Bioinformatics. 2015:btv285.

236. Tautenhahn R, Patti GJ, Rinehart D and Siuzdak G. XCMS Online: a web-based platform to process untargeted metabolomic data. Analytical Chemistry. 2012;84 11:5035-9.

237. Grace SC, Embry S and Luo H. Haystack, a web-based tool for metabolomics research. BMC Bioinformatics. 2014;15 11:S12.

238. Liang Y-J, Lin Y-T, Chen C-W, Lin C-W, Chao K-M, Pan W-H, et al. SMART: Statistical Metabolomics Analysis� An R Tool. Analytical Chemistry. 2016;88 12:6334-41.

239. Pluskal T, Castillo S, Villar-Briones A and Orešič M. MZmine 2: modular framework for processing, visualizing, and analyzing mass spectrometry-based molecular profile data. BMC Bioinformatics. 2010;11 1:395.
240. Wei X, Sun W, Shi X, Koo I, Wang B, Zhang J, et al. MetSign: a computational platform for high-resolution mass spectrometry-based metabolomics. Analytical Chemistry. 2011;83 20:7668-75.
241. LaMarche BL, Crowell KL, Jaitly N, Petyuk VA, Shah AR, Polpitiya AD, et al. MultiAlign: a multiple LC-MS analysis tool for targeted omics analysis. BMC Bioinformatics. 2013;14 1:49.
242. Carroll AJ, Badger MR and Millar AH. The MetabolomeExpress Project: enabling web-based processing, analysis and transparent dissemination of GC/MS metabolomics datasets. BMC Bioinformatics. 2010;11 1:376.
243. Fernández-Albert F, Llorach R, Andrés-Lacueva C and Perera A. An R package to analyse LC/MS metabolomic data: MAIT (Metabolite Automatic Identification Toolkit). Bioinformatics. 2014;30 13:1937-9.
244. Melamud E, Vastag L and Rabinowitz JD. Metabolomic analysis and visualization engine for LC− MS data. Analytical Chemistry. 2010;82 23:9818-26.
245. Neuweger H, Albaum SP, Dondrup M, Persicke M, Watt T, Niehaus K, et al. MeltDB: a software platform for the analysis and integration of metabolomics experiment data. Bioinformatics. 2008;24 23:2726-32.
246. Xia J, Sinelnikov IV, Han B and Wishart DS. MetaboAnalyst 3.0—making metabolomics more meaningful. Nucleic acids research. 2015;43 W1:W251-W7.
247. Kaever A, Landesfeind M, Feussner K, Mosblech A, Heilmann I, Morgenstern B, et al. MarVis-Pathway: integrative and exploratory pathway analysis of non-targeted metabolomics data. Metabolomics. 2015;11 3:764-77.
248. Edmands WM, Barupal DK and Scalbert A. MetMSLine: an automated and fully integrated pipeline for rapid processing of high-resolution LC-MS metabolomic datasets. Bioinformatics. 2014:btu705.
249. Beisken S, Earll M, Portwood D, Seymour M and Steinbeck C. MassCascade: Visual Programming for LC-MS Data Processing in Metabolomics. Molecular informatics. 2014;33 4:307-10.
250. Winkler R. MASSyPup—an 'Out of the Box'solution for the analysis of mass spectrometry data. Journal of Mass Spectrometry. 2014;49 1:37-42.
251. Sakurai N, Ara T, Enomoto M, Motegi T, Morishita Y, Kurabayashi A, et al. Tools and databases of the KOMICS web portal for preprocessing, mining, and dissemination of metabolomics data. BioMed Research International. 2014;2014.
252. Sakurai T, Yamada Y, Sawada Y, Matsuda F, Akiyama K, Shinozaki K, et al. PRIMe update: innovative content for plant metabolomics and integration of gene expression and metabolite accumulation. Plant and Cell Physiology. 2013;54 2:e5-e.
253. Henry VJ, Bandrowski AE, Pepin A-S, Gonzalez BJ and Desfeux A. OMICtools: an informative directory for multi-omic data analysis. Database. 2014;2014:bau069.
254. Sumner LW, Amberg A, Barrett D, Beale MH, Beger R, Daykin CA, et al. Proposed minimum reporting standards for chemical analysis. Metabolomics. 2007;3 3:211-21. doi:10.1007/s11306-007-0082-2.
255. Gago J, Daloso DdM, Figueroa CM, Flexas J, Fernie AR and Nikoloski Z. Relationships of Leaf Net Photosynthesis, Stomatal Conductance, and Mesophyll Conductance to Primary Metabolism: A Multispecies Meta-Analysis Approach. Plant Physiology. 2016;171 1:265-79. doi:10.1104/pp.15.01660.

Figure 1 Typical mass spectrometry based metabolomics workflow.

Additional file 1.xls Summary of resources for mass spectrometry based metabolomics.

Figure
Click here to download Figure GigaScienceReview_Figure_Submit.pptx

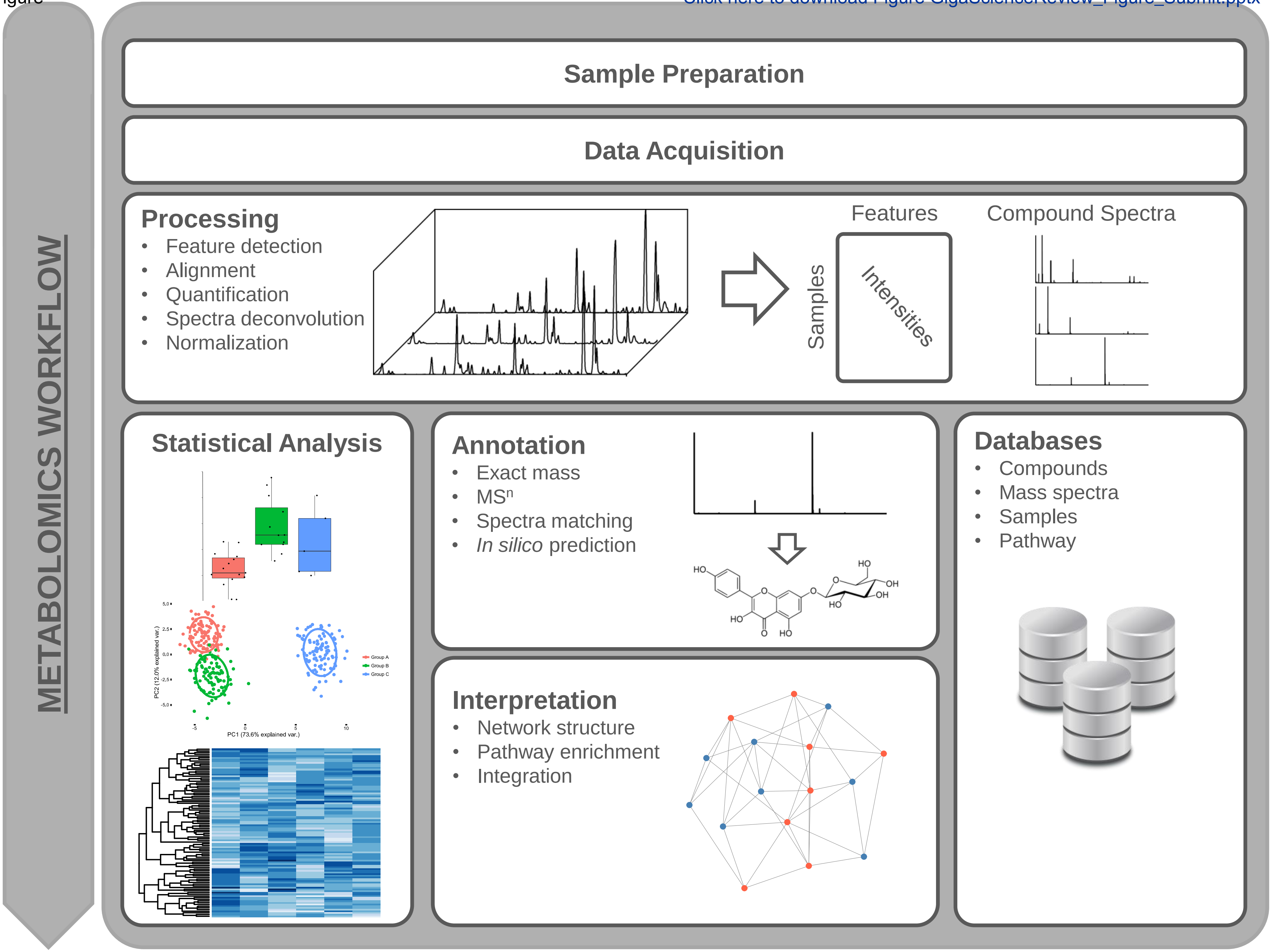

Click here to access/download
Supplementary Material
Databases_Submit.xls
