# Supplementary material for: From chromatogram to analyte to metabolite. How to pick horses for courses from the massive web resources for mass spectral plant metabolomics
Source: Gigascience. 2017 May 17;6(7):1–20. doi: 10.1093/gigascience/gix037 (PMC5499862; doi:10.1093/gigascience/gix037)
Supplement: Response_to_Reviewer_Comments_Original-Submission.pdf [file gix037_response_to_reviewer_comments_original-submission.pdf]

Author's Response To Reviewer Comments

Close

Reviewer reports:
Reviewer #1: This review has been well-written already, but I have some comments as listed below which should be considered by authors.

1. The paper is too long. Should all of the local- or web applications that you introduced be highlighted in this paper? As you mentioned in the future perspective, many of the tools are already 'out of dates', never updated for a long time, and never used for metabolomics research anymore. But I really feel a 'value' in this paper especially for an 'education' purpose too. Therefore, I highly would like authors to add 'the date of last update' for each tool (or as much as possible) cited in this manuscript. As you know, the evaluation of GO analysis tools is now performed like that:
http://www.nature.com/nmeth/journal/v13/n9/full/nmeth.3963.html?WT.ec_id=NMETH-201609&spMailingID=52180959&spUserID=MzcwMzk3NDY5OTES1&spJobID=985584826&spReportId=OTg1NTg0ODI2S0
Reply: As suggested by both reviewers, the problem of outdated tools available online is a major one. To highlight this in the paper we included a sentence in the background pointing out the importance of evaluating the current state of each resource and referred to the "last updated" dates included in supplementary table 1.
Regarding the extension of the manuscript, we briefly described even outdated tools so that the reader can have an idea of the previous developments leading to the current state-of-the-art in each respective step of the metabolomics pipeline.

I know your review is not for the evaluation. But you have to add the information of 'recommended'-, 'activity-', 'special interest' or 'outstanding interest' as a lot of reviews do. See like COCB reviews:
http://www.sciencedirect.com/science/journal/13675931/36/supp/C.
Reply: Included in supplementary table.

2. Please transfer ms2lda and ms2analyzer to 'annotation' section.
Reply: Transfered

3. I think MS-DIAL is not only for DIA-MS, but also all other techniques such as GC/MS and DDA.
Reply: Yes, indeed it is. We added a sentence to highlight this point.

4. Please transfer mathdamp and spectconnect to data processing section.
Reply: Transfered

5. In metabolite annotation section, cite CASMI, and see MS-FINDER and CSI-IOKR are also interesting tools which have been recently developed.
Reply: Added to annotation section, thanks for the suggestion!

6. UNPD database should be cited as natural product database.
Reply: Added to database section.

7. You said 'Metline currently contains 961,829 molecules'. Ok my question is: how many records do contain MS/MS information?
Reply: Included in text: "METLIN currently contains 961,829 molecules from which 200,000 have in silico MS/MS data. Additionally over 14,000 metabolites were analyzed and mass spectra at multiple collision energies in positive and negative ionization mode obtained".

I am looking forward to seeing your improved manuscript.
Thanks,

Reviewer #2: This is a very comprehensive and complete review of available tools and databases available to perform plant metabolomic analysis.

My only concern is that it may daunting for the reader to grasp the breadth and depth of all the possibilities available for her/him in the current format. The figure helps to get a broad view of the different steps required to perform this type of analysis. I suggest to include a table with available tools for the different steps in the data analysis pipeline and indicating the type of tool (GUI, command line) language (R, Java etc).
Reply: A table with the relevant description of all tools mentioned in the text was provided in supplementary data.

Other than that I only have some minors comments/corrections.

Reviewer #2:

l 38: add full stop or semicolon after Arabidopsis Thaliana.
Reply: Done
Reviewer #2:
l 78: change to: plant metabolic responses will be best exploited in the future
Reply: Done
Reviewer #2:
l 231 to 236: Break down this sentence in two. Too long to follow properly.
Reply: Done
Reviewer #2:
l 308: iterates instead of iterating
Reply: Done
Reviewer #2:
l 440: full stop after metabolites
Reply: Done
Reviewer #2:
l 463: describe SDF files.
Reply: Done
Reviewer #2:
l 575: Also include http://fiehnlab.ucdavis.edu/projects/fiehnlib
Reply: We previously did not include fiehnLib since we could not get access to the spectral data from the library. However we have added a comment to that effect in the revised manuscript.
Reviewer #2:
l 731: PlantCyc only has 22 species.
Reply: The plant metabolic network (PMN) includes single-species/taxon databases on 20 individual species, but at the center of PMN is PlantCyc with over 800 pathways of 350 plant species (http://www.plantcyc.org/about/plantcyc-species).
Reviewer #2:
l 743: Brachypodium instead of Bracypodium.
Reply: Done
Reviewer #2:
l 766: maybe cite here services that allow conversion between different types of metabolite chemical information like the chemical translation service: http://cts.fiehnlab.ucdavis.edu/
Reply: Fixed. It was only cited with the acronym CTS.
l 818: worth commenting here on persistence of web services and algorithms over time. Is very common that tools are made and then no longer maintained and supported. As an example, the muscleproject.org website, published in 2015, is not available. R packages in this regard do provide a better way to curate software through bioconductor and CRAN. (Nice review about this here: http://www.sciencedirect.com/science/article/pii/S1360138516301996)
Reply: We added a small section in the background pointing the issue and referring to the importance of the "update dates" provided in supplementary table. And briefly discussed the importance of repositories to keep these tools.
--
Please also take a moment to check our website at http://giga.edmgr.com/l.asp?i=8865&l=1KH5AEGW for any additional comments that were saved as attachments. Please note that as GigaScience has a policy of open peer review, you will be able to see the names of the reviewers.

Close
