# Supplementary material for: From chromatogram to analyte to metabolite. How to pick horses for courses from the massive web resources for mass spectral plant metabolomics
Source: Gigascience. 2017 May 17;6(7):1–20. doi: 10.1093/gigascience/gix037 (PMC5499862; doi:10.1093/gigascience/gix037)
Supplement: Reviewer_1_Report_(Original-Submission).pdf [file gix037_reviewer_1_report_(original-submission).pdf]

Cancel Save and Close

GIGA-D-17-00039
"From chromatogram to analyte to metabolite. How to pick horses for courses from the massive web-resources for mass spectral plant metabolomics"
Original Submission

Hiroshi Tsugawa (Reviewer 1)

**Reviewer Recommendation Term:**	Major Revision
**Rate Review:**	Please enter a number from 1-100 to rate this review (100 is best)

Custom Review Question(s)	Response
**Level of interest**<br>Please indicate how interesting you found the manuscript:	An article of importance in its field
**Quality of written English**<br>Please indicate the quality of language in the manuscript:	Acceptable
**Declaration of competing interests**<br>Please complete a declaration of competing interests, considering the following questions:<br>1. Have you in the past five years received reimbursements, fees, funding, or salary from an organisation that may in any way gain or lose financially from the publication of this manuscript, either now or in the future?<br>2. Do you hold any stocks or shares in an organisation that may in any way gain or lose financially from the publication of this manuscript, either now or in the future?<br>3. Do you hold or are you currently applying for any patents relating to the content of the manuscript?<br>4. Have you received reimbursements, fees, funding, or salary from an organization that holds or has applied for patents relating to the content of the manuscript?<br>5. Do you have any other financial competing interests?<br>6. Do you have any non-financial competing interests in relation to this paper?<br>If you can answer no to all of the above, write 'I declare that I have no competing interests' below. If your reply is yes to any, please give details below.	I declare that I have no competing interests.
I agree to the open peer review policy of the journal. I understand that my name will be included on my report to the authors and, if the manuscript is accepted for publication, my named report including any attachments I upload will be posted on the website along with the authors' responses. I agree for my report to be made available under an Open Access Creative Commons CC-BY license (http://creativecommons.org/licenses/by/4.0/). I understand that any comments which I do not wish to be included in my named report can be included as confidential comments to the editors, which will not be published.	☐ I agree to the open peer review policy of the journal
To further support our reviewers, we have joined with Publons, where you can gain additional credit to further highlight your hard work (see: https://publons.com/journal/530/gigascience). On publication of this paper, your review will be automatically added to Publons, you can then choose whether or not to claim your Publons credit. I understand this statement.	Yes

Comments to Editor:

Comments to Author:

This review has been well-written already, but I have some comments as listed below which should be considered by authors.

1. The paper is too long. Should all of the local- or web applications that you introduced be highlighted in this paper? As you mentioned in the future perspective, many of the tools are already 'out of dates', never updated for a long time, and never used for metabolomics research anymore. But I really feel a 'value' in this paper especially for an 'education' purpose too. Therefore, I highly would like authors to add 'the date of last update' for each tool (or as much as possible) cited in this manuscript. As you know, the evaluation of GO analysis tools is now performed like that:
http://www.nature.com/nmeth/journal/v13/n9/full/nmeth.3963.html?WT.ec_id=NMETH-201609&spMailingID=52180959&spUserID=MzcwMzk3NDY5OTES1&spJobID=985584826&spReportId=OTg1NTg0ODI2S0

I know your review is not for the evaluation. But you have to add the information of 'recommended'-, 'activity-', 'special interest' or 'outstanding interest' as a lot of reviews do. See like COCB reviews:
http://www.sciencedirect.com/science/journal/13675931/36/supp/C.

2. Please transfer ms2lda and ms2analyzer to 'annotation' section.
3. I think MS-DIAL is not only for DIA-MS, but also all other techniques such as GC/MS and DDA.
4. Please transfer mathdamp and spectconnect to data processing section.
5. In metabolite annotation section, cite CASMI, and see MS-FINDER and CSI-IOKR are also interesting tools which have been recently developed.
6. UNPD database should be cited as natural product database.
7. You said 'Metline currently contains 961,829 molecules'. Ok my question is: how many records do contain MS/MS information?

I am looking forward to seeing your improved manuscript.
Thanks,

More Reviewer Details

Cancel Save and Close
