# Supplementary material for: From chromatogram to analyte to metabolite. How to pick horses for courses from the massive web resources for mass spectral plant metabolomics
Source: Gigascience. 2017 May 17;6(7):1–20. doi: 10.1093/gigascience/gix037 (PMC5499862; doi:10.1093/gigascience/gix037)
Supplement: Reviewer_2_Report_(Original_Submission).pdf [file gix037_reviewer_2_report_(original_submission).pdf]

Cancel Save and Close

GIGA-D-17-00039
"From chromatogram to analyte to metabolite. How to pick horses for courses from the massive web-resources for mass spectral plant metabolomics"
Original Submission

Ruben Rellan (Reviewer 2)

**Reviewer Recommendation Term:**	Minor Revision
**Rate Review:**	Please enter a number from 1-100 to rate this review (100 is best)

Custom Review Question(s)	Response
**Level of interest**<br>Please indicate how interesting you found the manuscript:	An article of importance in its field
**Quality of written English**<br>Please indicate the quality of language in the manuscript:	Acceptable
**Declaration of competing interests**<br>Please complete a declaration of competing interests, considering the following questions:<br><br>1. Have you in the past five years received reimbursements, fees, funding, or salary from an organisation that may in any way gain or lose financially from the publication of this manuscript, either now or in the future?<br>2. Do you hold any stocks or shares in an organisation that may in any way gain or lose financially from the publication of this manuscript, either now or in the future?<br>3. Do you hold or are you currently applying for any patents relating to the content of the manuscript?<br>4. Have you received reimbursements, fees, funding, or salary from an organization that holds or has applied for patents relating to the content of the manuscript?<br>5. Do you have any other financial competing interests?<br>6. Do you have any non-financial competing interests in relation to this paper?<br><br>If you can answer no to all of the above, write 'I declare that I have no competing interests' below. If your reply is yes to any, please give details below.	I declare that I have no competing interests
I agree to the open peer review policy of the journal. I understand that my name will be included on my report to the authors and, if the manuscript is accepted for publication, my named report including any attachments I upload will be posted on the website along with the authors' responses. I agree for my report to be made available under an Open Access Creative Commons CC-BY license (http://creativecommons.org/licenses/by/4.0/). I understand that any comments which I do not wish to be included in my named report can be included as confidential comments to the editors, which will not be published.	☐ I agree to the open peer review policy of the journal
To further support our reviewers, we have joined with Publons, where you can gain additional credit to further highlight your hard work (see: https://publons.com/journal/530/gigascience). On publication of this paper, your review will be automatically	Yes

added to Publons, you can then choose whether or not to claim your Publons credit. I understand this statement.

Comments to Editor:

One wonders if there could be other types of literature review of software/databases that would be more helpful for the reader/user. This review is perfectly fine and comprehensive covering all the software that is available for the different steps of metabolomics data analysis and available databases. But, it´s difficult for the reader to get a grasp of what it would be the best way to analyze his/her own data. I would personally favor the idea of using a dataset and guide the reader trough the multiple options that would be available for him/her. Since a great deal of the tools presented are in R a tutorial like R-markdown document would be a very helpful for the reader to have. At the end of the day most people will read the review to figure out how the can analyze their own data.

Maybe it would be a good idea for a journal like Gigascience to consider this type of articles? Something like Nature Protocols but for code?

See for example:
https://link.springer.com/protocol/10.1007/978-1-62703-580-4_5

Comments to Author:

This is a very comprehensive and complete review of available tools and databases available to perform plant metabolomic analysis.

My only concern is that it may daunting for the reader to grasp the breadth and depth of all the possibilities available for her/him in the current format. The figure helps to get a broad view of the different steps required to perform this type of analysis. I suggest to include a table with available tools for the different steps in the data analysis pipeline and indicating the type of tool (GUI, command line) language (R, Java etc).

Other than that I only have some minors comments/corrections.

l 38: add full stop or semicolon after Arabidopsis Thaliana.
l 78: change to: plant metabolic responses will be best exploited in the future
l 231 to 236: Break down this sentence in two. Too long to follow properly.
l 308: iterates instead of iterating
l 440: full stop after metabolites
l 463: describe SDF files.
l 575: Also include http://fiehnlab.ucdavis.edu/projects/fiehnlib
l 731: PlantCyc only has 22 species.
l 743: Brachypodium instead of Bracypodium.
l 766: maybe cite here services that allow conversion between different types of metabolite chemical information like the chemical translation service: http://cts.fiehnlab.ucdavis.edu/
l 818: worth commenting here on persistence of web services and algorithms over time. Is very common that tools are made and then no longer maintained and supported. As an example, the muscleproject.org website, published in 2015, is not available. R packages in this regard do provide a better way to curate software through bioconductor and CRAN. (Nice review about this here:
http://www.sciencedirect.com/science/article/pii/S1360138516301996)

More Reviewer Details

Cancel Save and Close
